# Dispersal diversity buffers species vulnerability to local extinction

**DOI:** 10.1038/s41559-026-03195-y

**Published:** 2026-10-09

**Authors:** Davide Bernardi, Giorgio Nicoletti, Prajwal Padmanabha, Samir Suweis, Sandro Azaele, Simon A. Levin, Andrea Rinaldo, Amos Maritan

**Affiliations:** 1 Laboratory of Interdisciplinary Physics, Department of Physics and Astronomy, University of Padova, Padova, Italy; 2 National Biodiversity Future Center, Palermo, Italy; 3 Quantitative Life Sciences, The Abdus Salam International Centre for Theoretical Physics, Trieste, Italy; 4 Department of Fundamental Microbiology, University of Lausanne, Lausanne, Switzerland; 5 Department of Ecology and Evolutionary Biology, Princeton University, Princeton, NJ USA; 6 High Meadows Environmental Institute, Princeton University, Princeton, NJ USA; 7 CMCC Foundation, Euro Mediterranean Center on Climate Change, Lecce, Italy; 8 Faculty ENAC, École Polytechnique Fédérale de Lausanne, Lausanne, Switzerland

**Keywords:** Theoretical ecology, Population dynamics, Community ecology

## Abstract

Predicting species persistence within ecological communities is a fundamental challenge for both empirical and theoretical ecology. Existing methods span from mechanistic models, whose parameters are difficult to estimate from data, to statistical tools whose context-specific parameters are less interpretable. Here we present a general framework, grounded in the statistical physics of complex systems, that integrates the key processes governing species survival into a single measurable quantity: the competitive balance. This metric quantifies the vulnerability of a focal species to competitive exclusion beyond what is captured by its abundance alone by incorporating the diversity of dispersal strategies and the structure of interspecific interactions within the community. Crucially, the competitive balance can be inferred from spatial abundance data, thus circumventing the need to estimate species traits or dispersal parameters. Our results reveal that greater heterogeneity in dispersal strategies reduces vulnerability to competitive exclusion for a given abundance. We validate the framework using tropical and temperate forest data and discuss how it can be applied to a range of different ecosystems, providing a systemic and interpretable tool for assessing a context-dependent species vulnerability that accounts for its interactions with the entire community.

## Abstract (web summary)

Outlining an analytical framework for quantifying species vulnerability that captures abundance, dispersal and interspecific interactions, the authors show that dispersal diversity buffers against local extinction.

## Main

Human impacts on biodiversity have prompted concerns about a potential mass extinction^1^. Yet, despite signals such as population declines and range contractions, the magnitude and the trajectory of ongoing extinctions remain debated^2^. Resolving these uncertainties requires theoretical frameworks that link local population dynamics to spatial community structure across scales^3^ and that can be ultimately validated with empirical data^4^.

Ecological theories have made substantial progress on different aspects of this problem. Coexistence theory details broad mechanisms by which species persist together^5–8^, with recent work extending these ideas to empirical measurement^9^, demographic uncertainty^10^ and community-level characterizations of the feasibility regions^11,12^. Metapopulation^13^ and metacommunity^14^ frameworks have shown how spatial structure and dispersal shape diversity^15^, while broader theoretical syntheses have connected biodiversity to ecosystem stability^16^, life-history variation^17^ and macroecological patterns^18^. Yet, these advances have largely proceeded in parallel: analytical tractability for species-rich communities has been achieved mainly in non-spatial settings^11,19^, while spatially explicit models remain difficult to scale to many interacting species.

Spatial heterogeneity shapes coexistence in several ways: through stabilizing mechanisms such as the storage effect and dispersal limitation^20,21^, by giving rise to interspecific spatial associations^22^ and by modulating coexistence–area relationships through the configuration of species interactions^23^. Empirical tools for quantifying these patterns include spatial point pattern analysis^24^ and joint species distribution models^25^. Recent work has begun to integrate spatial patterns with interaction-based descriptions of communities^26–28^. Yet, integrating spatial structure into mechanistic models of interacting communities remains challenging. Analytical results typically become intractable, whereas numerical simulations depend sensitively on model assumptions and parameterization and offer limited scalability^29^.

On the empirical side, data-driven methods (such as species distribution models^30^, occupancy scaling methods^31,32^, population viability analysis^33^ or matrix population models^34^) combine statistical inference with some mechanistic elements, but often focus on single-species dynamics and typically require detailed demographic and environmental data^35,36^. Hence, there is a need for frameworks that combine spatial structure with multispecies interactions in a way that is analytically tractable and compatible with the coarse-grained data available for most species.

Here we introduce an analytical framework to quantify the proximity of each species to its competitive exclusion boundary in metacommunities. Our approach uses a parsimonious ecological model explicitly incorporating basic processes taking place in a heterogeneous spatial environment. Leveraging analytical methods from the physics of disordered systems, we derive a set of ecologically interpretable metrics that quantify how species exploit locally available space and how they are affected by community-mediated interactions. These metrics define species vulnerability as their proximity to the competitive-exclusion threshold, explicitly accounting for the ecological context in which they are embedded. Importantly, our approach does not require previous knowledge of species-specific traits, demographic parameters or interaction coefficients, and can be applied directly to empirical data. This is especially valuable in studying species-rich communities, where detailed species-level information is often unavailable and might be strongly context-dependent. A summary of the main metrics and definitions used throughout the study is provided in Table 1.

**Table 1:** Metrics and definitions introduced in this study

Description	Symbol/mathematical definition
Space-use fraction: fraction of the total effective space available in site (or subplot) *i* occupied by species *α*. For forest data, the effective space is estimated from the allometric relation between trunk diameter and crown area^44,45^. If diameter data are not available, this can be approximated using stem counts.	${p}_{\alpha i}={\sum }_{j}{d}_{i\alpha ,\,j}^{4/3}/M$, where *d*_*iα*,*j*_ is the trunk diameter of the *j*th individual belonging to species *α* at site *i* and *M* is the total effective space per site. It is estimated from the data from a global fitting procedure (Methods)
Total fraction of occupied space: average space-use fraction or total fraction of effective available space occupied by species *α* (across the entire sampled area).	〈*p*_*α*_〉, where 〈 〉 indicates average over subplots (sites)
VAA: space-use of species *α* in subplot *i*, normalized by the locally available (unoccupied) space. By accounting for local space saturation, the VAA rescales relative space occupation to reflect the effective colonization effort. As a result, identical relative abundances yield higher VAA values in space-limited sites and lower values in less saturated sites. The dynamical mean-field calculation provides an analytical expression for the VAA distribution that can be fitted to spatial census data.	${x}_{\alpha i}=\frac{{p}_{\alpha i}}{1-{\sum }_{\beta }{p}_{\beta i}}$
Interaction-dispersal kernel: species-specific matrix describing interaction and dispersal processes of species *α*. The kernel matrix *K*_*α*_ bundles into a single quantity local birth and growth rates, colonization-mediated dispersal and local interactions of species *α* with other species, such as competition and facilitation (in the form of effective competitive suppression). Its spatial distribution is assumed to be a species-specific gamma distribution. It is not directly measurable, but its parameters are inferred from the data by fitting the analytical theory for the VAA to the empirical VAA distribution.	*K*_*α*_
Dispersal heterogeneity: spatial variability of the dispersal strategy. The dispersal heterogeneity of species *α* is defined as the inverse shape parameter of the VAA distribution, ${\delta }_{\alpha }^{-1}$, which is equal to the squared coefficient of variation of the VAA distribution. High ${\delta }_{\alpha }^{-1}$ reflects patchy, spatially aggregated abundance; low ${\delta }_{\alpha }^{-1}$ reflects a more spatially homogeneous distribution.	${\delta }_{\alpha }^{-1}$
Effective dispersal strength: scale (or inverse rate) parameter of the VAA distribution. The theory links it to the average of the *K*_*α*_ distribution, adjusted by the average abundance and local death rate (Methods). It is related to the effective dispersal strength of the species, relative to the community level.	${\bar{\beta }}_{\alpha }^{-1}$
Competitive balance: ratio between community-wide factors that increase and decrease abundance. *B*_*α*_ > 1 is necessary for persistence in isolation; persistence of species *α* in the community requires *B*_*α*_ to be not too far below the equal-balance condition. The competitive balance is inferred indirectly from data via a self-consistent integral equation coupling VAA parameters of all species. This equation does not additively sum the competitive balances of all species, but it determines the metacommunity composition through an implicit nonlinear equation.	${B}_{\alpha }=\left(\int_{0}^{\infty }d\lambda \frac{{e}^{-\lambda -{\sum }_{\gamma }{\delta }_{\gamma }{\ln}\left(1+\lambda /({\bar{\beta }}_{\gamma })\right)}}{1+\lambda /{\bar{\beta }}_{\alpha }}\right)^{-1}$
Species vulnerability: rescaled distance to the persistence threshold of each species. The critical value is zero. Species with negative vulnerability persist in the long term; thus, a more negative vulnerability (larger ∣*W*_*α*_∣) indicates lower susceptibility to competitive exclusion. Species vulnerability is computed from the competitive balances and the kernel parameters of all species, so it depends on the ecological context. The same species can have a different vulnerability in different communities, depending on their composition.	${W}_{\alpha }=\frac{1}{| {\Delta }_{\alpha }| }\left(\left({\left[{B}_{\alpha }\right]}_{\alpha }-{B}_{\alpha }\right)-{\Delta }_{\alpha }\right)$, where ${\left[.\right]}_{\alpha }$ indicates average over species; *Δ*_*α*_ is the rescaled distance of the community average competitive balance to the persistence threshold of species *α*. See Methods, equation (24) for its explicit expression.

Backed by comparison to forest datasets, we exploit our framework to show that species vulnerability cannot be fully predicted by abundance alone. Rather, we explicitly quantify how the entanglement among dispersal strategies, spatial distribution patterns and total population size determines the proximity of species to exclusion from a metacommunity, capturing an ecosystem-level perspective on long-term persistence that moves beyond single-species metrics and risk assessments based solely on demographic fluctuations. Further, although our metrics are derived analytically, they remain interpretable without relying on model specifics, suggesting broader ecological relevance.

## Results

### Statistical physics-based theory of spatially explicit ecosystems

We study a general metacommunity model describing the temporal dynamics of species abundances in a habitat composed of *N* colonizable sites, each providing a finite amount of available space or a limiting resource. These sites may correspond to distinct patches, although our framework does not strictly require explicit physical boundaries. The model incorporates basic processes such as death, reproduction, dispersal, colonization and competition for space. Their combined effect is captured by an interaction-dispersal kernel matrix, *K*, whose elements represent a coarse-grained description of how species interact and spread across patches. However, they do not explicitly separate local competitive and facilitative interactions, but capture instead their net effect on dispersal efficacy.

The mathematical structure of the model aligns with the metacommunity framework^13^, widely used to study species survival in fragmented landscapes^37–39^. However, measuring interaction kernels or the corresponding dispersal networks in real ecosystems remains challenging. In light of this experimental inaccessibility, we posit that the intrinsic variability in habitats, species traits and interspecific interactions in large-scale natural ecosystems will lead to entries in the kernel that are effectively random. We model this randomness by drawing kernel elements from a gamma distribution (Methods). The parameters of this distribution are species-specific, meaning that each species has its own parameter pair, that quantitatively captures diverse colonization strategies. These parameters are not prescribed a priori, but inferred directly from observed spatial abundance data.

The link between model and data is provided by a metric that emerges from the analytical solution of the model using generalized dynamical mean-field theory (DMFT)^40^: the vacancy-adjusted abundance (VAA). The VAA of species *α* at a given site is defined as the fraction of space it occupies divided by the locally available (unoccupied) space. This normalization accounts for local space saturation, making the VAA dependent on how empty a local site is relative to its capacity. Therefore, a species with the same relative space-use in different subplots contributes differently to the VAA depending on how much each patch is occupied: a subplot with very low free space amplifies the VAA for species occupying this spot and it reduces it in subplots that have larger free space. Although the VAA arises naturally from the DMFT calculation (Methods), it has a clear ecological interpretation: by upweighting space-limited patches, it rescales local relative space occupation to reflect the effective colonization effort.

**Fig. 1:**
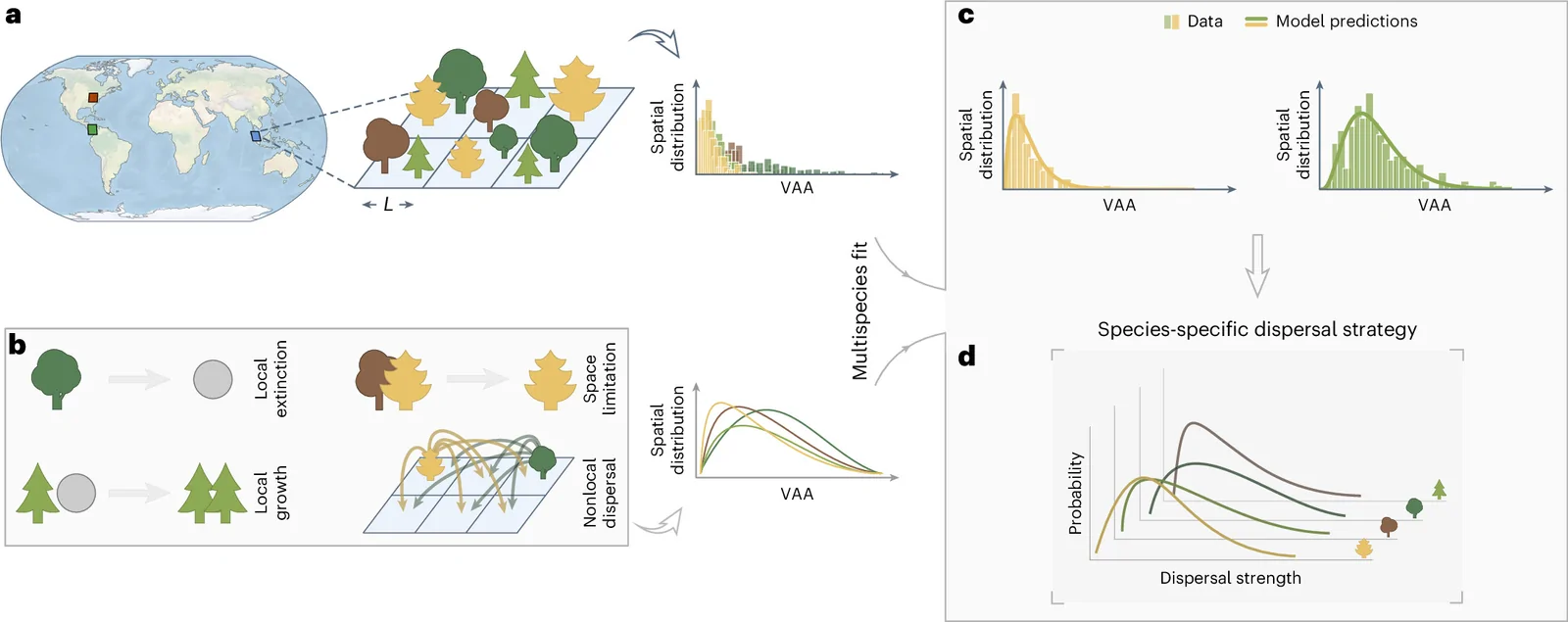
Fitting theory-predicted VAAD to data enables extraction of species-specific interaction-dispersal strategies. **a**, Forest census data: spatial tree census data are aggregated into square subplots of size *L* and converted into species-specific local space-use fractions *p*_*αi*_, where *α* denotes species and *i* spatial location. These are normalized by the locally unoccupied space—estimated during fitting—to obtain the empirical VAAD, which rescales relative abundance to account for local space saturation and capture colonization effort (Table 1). **b**, Dynamical metacommunity model: using methods from statistical physics applied to a parsimonious metacommunity model with growth, death, competition for space and dispersal, we derive an analytical prediction for the VAAD (Methods). **c**, The parameters of the theoretical VAAD are linked to those of the interaction-dispersal kernel through an implicit integral equation. **d**, Fitting the theory to data therefore enables inference of this kernel, which encodes species-specific spatial dispersal strategies. Hence, this inference does not require direct measurements of vital rates, demographic rates or interaction coefficients. Tree icons in **a**, **b** and **d** from SVG Repo (https://www.svgrepo.com) under a Creative Commons license CC0 1.0.

For each species, the parameters of the VAA distribution quantify its dispersal heterogeneity (${\delta }_{\alpha }^{-1}$ in Table 1) and an effective dispersal strength (${\bar{\beta }}_{\alpha }^{-1}$ in Table 1), which depends on the surrounding community. Fitting empirical VAA distributions therefore enables inference of species-specific dispersal strategies without requiring direct measurements of dispersal or interaction parameters. Because the parameters of the theoretical vacancy-adjusted abundance distributions (VAAD) are linked to those of the interaction-dispersal kernel through an implicit integral equation, fitting the theory to data enables inference of this kernel, which encodes species-specific spatial strategies. In this way, the VAA distribution provides a direct link between observable space-use patterns and the underlying interaction–colonization structure, while remaining a model-independent quantity that can be estimated from spatial abundance data. The overall conceptual pipeline is illustrated in Fig. 1.

### Theory predicts species-specific VAA distributions in forest plots

We validate our theory on three forest datasets: the Barro Colorado Island (BCI) tropical forest plot in Panama^41^, the Pasoh Forest Reserve tropical forest plot in Malaysia^42^ and the Michigan Big Woods temperate forest plot in the USA^43^. Each dataset provides a spatially complete census of all trees and thus offers an ideal testbed for our framework, which models spatially explicit abundances of species competing for shared space.

We computed the VAA empirical distributions from data, as described in the Methods. Briefly, we partition each plot into square subplots of side length *L* = 50 m, a spatial aggregation scale that balances sufficient sampling within subplots with the need to retain spatial resolution (Supplementary Information, section 1, and Supplementary Fig. 2, for details of optimal scale selection); we then estimate the space used by each species in each subplot by accounting for the size of each tree, following the established allometric scaling relation between trunk and crown size^44,45^; next, we infer the per-subplot effective total space capacity using a global fitting procedure. Finally, we construct the empirical VAA histogram as per its definition (Table 1).

**Fig. 2:**
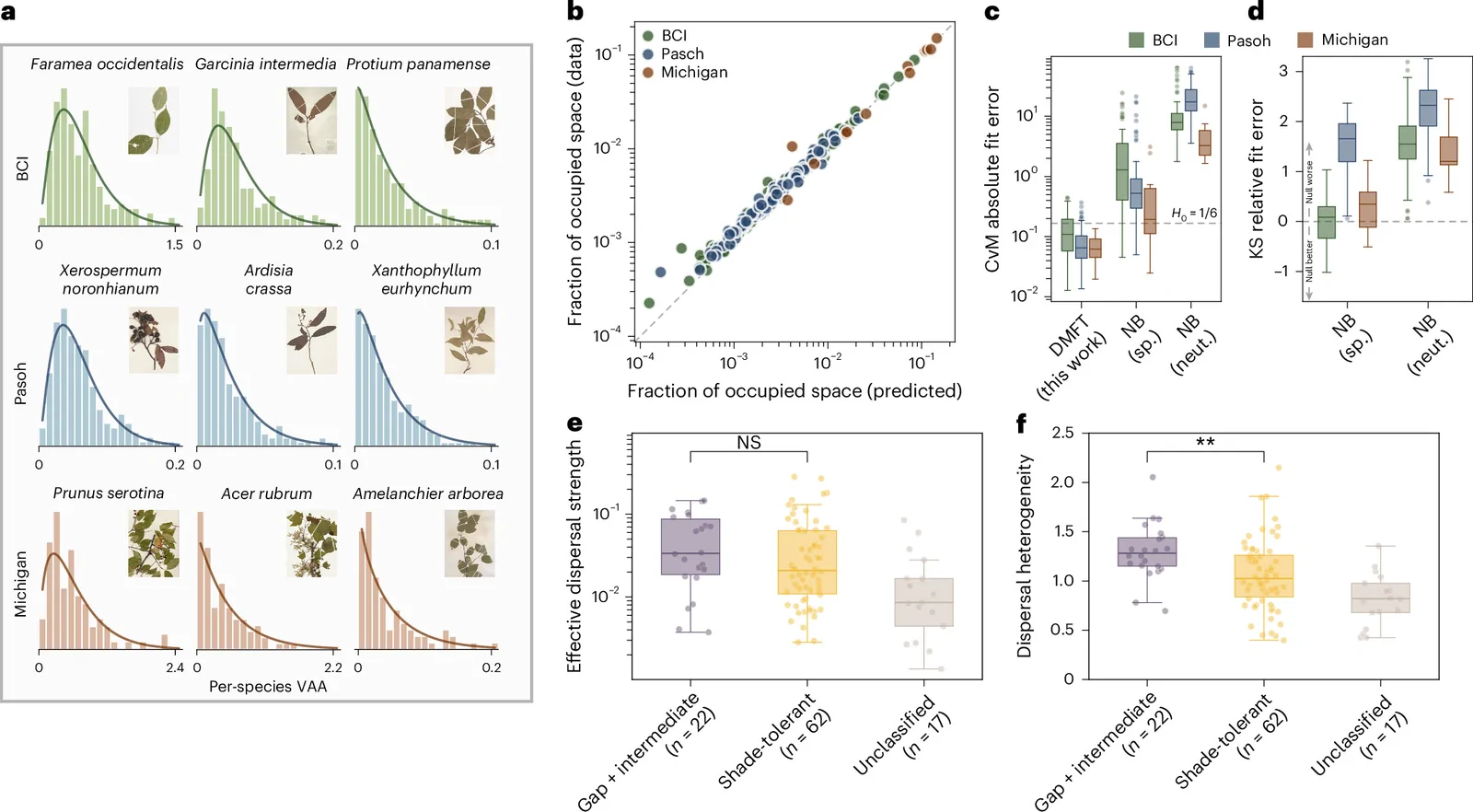
Theory accurately predicts species-specific VAA distributions from forest tree census data. VAA parameters reflect species dispersal and life-history strategies. **a**, Metacommunity model fits: empirical VAA distributions (histograms) with theory (lines) and for representative species from the BCI, Pasoh and Michigan forest plots (full report in Supplementary Information). **b**, Out-of-sample theoretical prediction for mean fraction of space-use by species *α*, 〈*p*_*α*_〉, versus observed 〈*p*_*α*_〉. Points closely follow the identity line across all three datasets (for BCI, Pasoh and Michigan: *R*^2^ = 0.97, 0.93, 0.95; *n* = 100, 100, 12 number of species, respectively). **c**,**d**, Comparison with non-interacting overdispersed null models based on negative binomial fitting of species counts (Methods and Supplementary Information, section 6). Two negative-binomial (NB) null model variants are considered: a neutral variant (NB neut.) with the same fit parameters for all species, and a variant with species-specific fit parameters (NB sp.). Our model not only strongly outperforms the neutral model, but also performs better than a species-specific model where the number of parameters are matched. The absolute fit error (**c**) is based on a Cramér–von Mises (CvM) proxy (dashed line indicates expectation for a correctly specified model) and the relative fit error (**d**) measures the log-ratio of the Kolmogorov–Smirnov statistic (positive ratio means that the null model is worse; Methods and Supplementary Information, section 6). In **c** and **d**, each box summarizes the per-species distribution of the fit-error metric over the *n* = 100, 100 and 12 most-abundant analysed species (BCI, Pasoh and Michigan, respectively; the same species shown in **b**), coloured by dataset (colour key in panel). **e**,**f**, Effective dispersal strength (${\bar{\beta }}_{\alpha }^{-1}$) (**e**) and dispersal heterogeneity (${\delta }_{\alpha }^{-1}$) (**f**) by ecological guild for BCI data (*n* = 22 gap + intermediate, 62 shade-tolerant and 17 unclassified species; guild assignments from ref. ^49^). Guilds were compared with two-sided Mann–Whitney *U*-tests, Holm-corrected for several comparisons; unclassified species are shown but excluded from testing. Gap and intermediate species have significantly higher dispersal heterogeneity ${\delta }_{\alpha }^{-1}$ than do shade-tolerant species (**f**, *U* = 969, **P = 0.004), indicating that shade-tolerant species show more spatially homogeneous abundance distributions, whereas effective dispersal strength ${\bar{\beta }}_{\alpha }^{-1}$ does not differ significantly between the two groups (**e**, *U* = 815, *P* = 0.18, not significant (NS)). Significance markers: NS *P* > 0.05, *P ≤ 0.05, **P ≤ 0.01. Box plots (**c**–**f**): the centre line is the median, the box spans the interquartile range (25th–75th percentiles) and whiskers extend to the most extreme value within 1.5× interquartile range of the box; in **c** and **d** points beyond the whiskers are drawn as faded dots (outliers) and in **e** and **f** all individual species are overlaid as jittered dots. Credit: Photos in **a** reproduced under a Creative Commons license: *Faramea occidentalis*, Intermountain Herbarium, Utah State University^75^; *Garcinia* *intermedia* and *Amelanchier* *arborea*, Smithsonian Institution^76^; *Protium* *panamense* and *Acer* *rubrum*, Naturalis Biodiversity Center^77^; and *Prunus* *serotina*, University of New Hampshire^78^ (CC0 1.0); *Xerospermum* *noronhianum*, *Ardisia* *crassa* and *Xanthophyllum* *eurhynchum*, Royal Botanic Gardens, Kew^79^ (CC BY 4.0).

Figure 2a compares the theoretical VAA predictions, fitted to the empirical distributions, for three representative species per dataset. We observe excellent agreement, with the model accurately capturing the diversity of distributional shapes across species. These differences are naturally accounted for by species-specific dispersal strategies (Methods). Goodness-of-fit analyses for all analysed species, along with explicit visualizations for 212 species (Supplementary Figs. 3–5), are reported in Supplementary Information, section 1.

Next, we performed a series of model robustness checks. First, because the net effect of space limitation is competitive, we tested whether explicitly incorporating facilitation^8,46^—beyond the competition buffering implicitly encoded in the inferred parameters of *K*—could improve model performance. This extension resulted in only marginal changes in model fit across the three forest datasets (Supplementary Information, section 5). This suggests that, at the spatial and temporal scales considered here, the dominant effects of facilitation may already be effectively captured by the inferred kernel parameters.

Then, we investigated whether our model generalizes to unseen data. Specifically, we used the parameters inferred on half the forest plot (with different random partitions) to predict the mean fraction of space occupied by each species in the remaining half. This out-of-sample validation of our theory resulted in a remarkable agreement for all three datasets (Fig. 2b), showing that our analytical predictions are well-aligned with empirical observations.

As a final check, we compared our framework against ecological null models in which species are independent and overdispersed, leading naturally to a negative binomial description of abundances^47^. We considered two variants (Methods): a neutral model parameterized at the community level, closely related to neutral theories of biodiversity that reproduce several large-scale ecological patterns in species-rich systems^48^; and a species-specific model fitted independently for each species, matching the total number of parameters of our framework. We evaluated model performance by fitting the VAA distributions of all species across the three datasets (Methods). For each dataset, the absolute goodness-of-fit of our model is consistent with the reference level expected for a correctly specified model (Fig. 2c, dashed line). By contrast, the species-specific model provides an adequate fit only for the Michigan dataset, whereas the neutral model exhibits substantially larger errors across all datasets, suggesting model misspecification. In a relative goodness-of-fit comparison, our framework consistently outperforms the neutral model across all datasets (positive Kolmogorov–Smirnov (KS) log-ratio in Fig. 2d). Relative to the species-specific model, it yields a marked improvement in Pasoh and Michigan and a comparable, slightly better performance in BCI. Full details are provided in the Supplementary Information, section 6, in which an intermediate variant with partially shared parameters is also analysed. Additional analyses of data requirements (Supplementary Information, section 7) show that reliable parameter estimation is achievable even for smaller datasets and provide lower-bound estimates on data requirements.

Crucially, besides achieving a better fit to the VAA distributions, our model also yields two species-specific parameters that carry clear ecological information: dispersal heterogeneity and effective dispersal strength (Table 1). On the basis of the meaning of the VAA, we hypothesize that VAA heterogeneity also reflects differences in life-history strategies. To test this, we compared parameter values across functional guilds in the BCI dataset (shade-tolerant, gap-exploiting and intermediate; classification from ref. ^49^), focusing on a grouped comparison of gap-exploiting and intermediate species versus shade-tolerant species. We found no statistically significant difference between guilds in the effective dispersal strength (Fig. 2e; see caption for details on the statistical test). By contrast, gap-exploiting and intermediate species exhibit significantly larger dispersal heterogeneity than do shade-tolerant species (Fig. 2f). This difference between gap exploiters and shade-tolerant species remains significant when treating all three guilds separately (Supplementary Information, section 3). These results are consistent with the role of canopy gap dynamics in promoting episodic local dominance of light-demanding species^50,51^ and with recent work highlighting the role of life-history strategies in shaping the responses of species to environmental variability and enabling coexistence^17,52^.

### Competitive balance as the keystone of species coexistence

Our theoretical predictions go beyond capturing the shape of VAA distributions and connecting their parameters to the dispersal strategies of species; they also reveal how these parameters relate to the long-term persistence of species within the community. In classic metapopulation models that focus on a single species and do not model others explicitly, persistence occurs when the local extinction rate is lower than the so-called metapopulation capacity, typically quantified by the largest eigenvalue of the kernel matrix^37,53^. However, when several competing species are considered, the metapopulation capacity of each species provides a necessary but not sufficient condition for persistence^39^. In our case, we show that species persistence within an interacting community is determined by how a specific combination of single-species traits, which we term competitive balance, compares to the competitive balance of all other species (Methods).

Our calculations provide an explicit integral equation that connects the competitive balance of each species to the VAA distribution parameters of all species within the community (Table 1). The structure of this equation has two important consequences: first, competitive balances are not simply additive, but coupled nonlinearly through a shared term that makes community-level coexistence emerge as a collective property rather than a sum of individual contributions; second, it enables the inference of all competitive balances from data. Intuitively, competitive balance reflects how community-wide processes influence each species-specific ratio between factors that increase abundance and those that decrease it. A species can persist if its competitive balance is both greater than one, indicating that it could at least sustain itself in the absence of competition and not too far below an equal-balance condition, a hypothetical scenario in which all species share identical balance values.

**Fig. 3:**
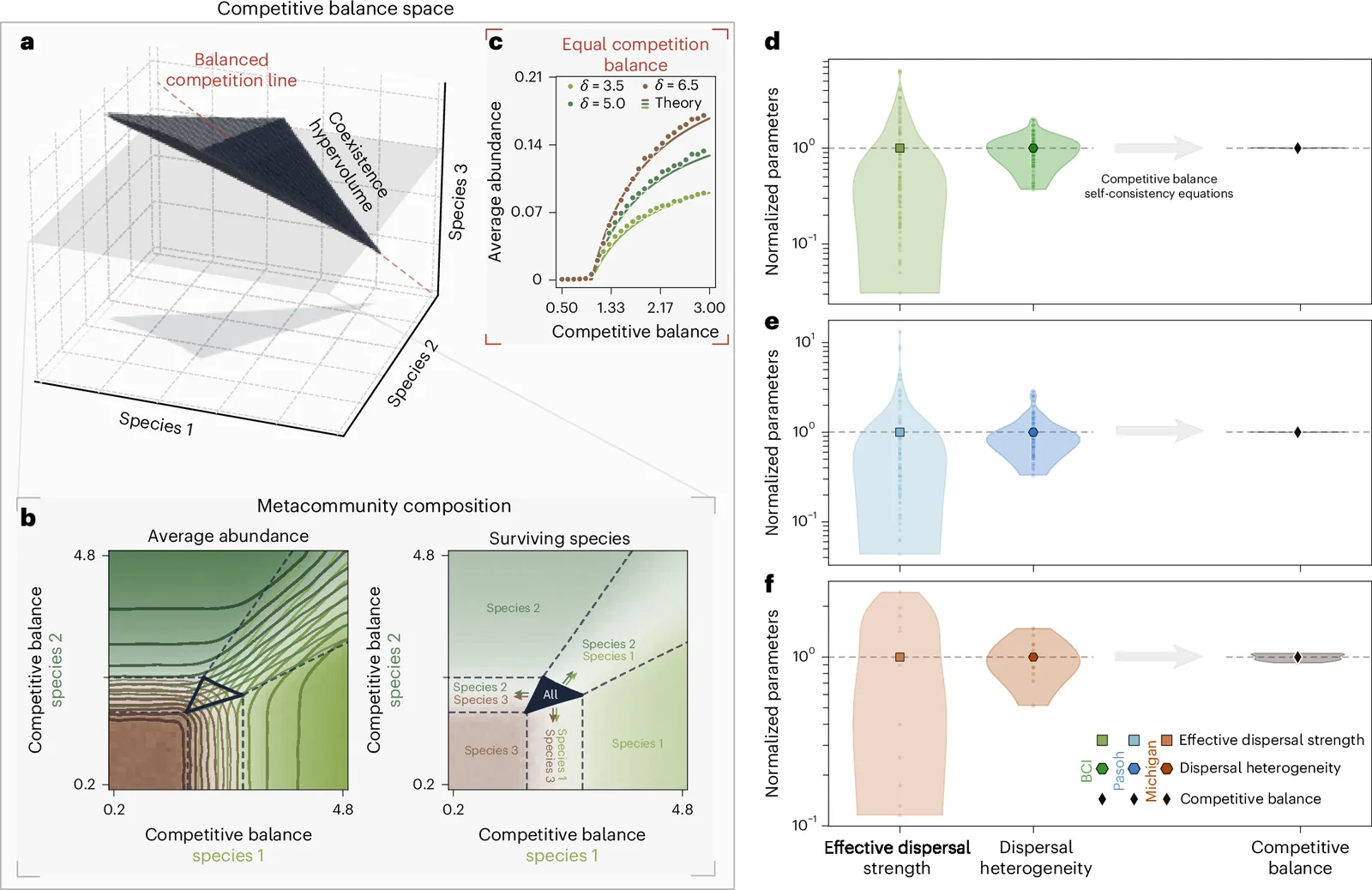
Theory reveals emergent colonization–extinction trade-off and identifies parameters governing survival and coexistence in species-rich communities. **a**, In the space defined by competitive balance values, coexistence of *S* species is possible only in a region where the balances of species are sufficiently similar. Here *S* = 3 for visualization. **b**, A two-dimensional slice of this space shows species abundances (colour intensity and contour lines) from numerical simulations as a function of the competitive balance of species 1 and 2, with species 3 held fixed. Theoretical predictions (critical boundaries) partition this space into distinct coexistence regimes. **c**, Simulations (circles) and theory (lines) show that species with identical competitive balances can have different abundances depending on community context (here the species have different dispersal heterogeneity; Table 1). **d**, Species-specific parameters—effective dispersal strength and dispersal heterogeneity (from VAA fits)—and competitive balances (computed from fitting parameters using the theory) are plotted relative to their means for BCI data. Each dot represents a species, with distributions visualized via kernel density estimates. Although dispersal-related parameters vary widely, competitive balances are tightly clustered around their mean, consistent with theoretical expectations. **e**, Same as **d** but for Pasoh data. **f**, Same as **d** and **e** for Michigan forest data.

This concept is illustrated in Fig. 3a using the example of three interacting species. In this representation, an ecological community corresponds to a point in a coordinate space defined by the competitive balance of each species. Our theory predicts the hypervolume in the competitive balance space where all species coexist, as well as the regions where all possible subsets of species coexist. Moreover, the theory predicts the species abundance distribution across sites, which depends on the competitive balance of all species (Methods). To visualize these predictions, we keep the competitive balance of species 3 fixed and vary the ones of the first two (Fig. 3b) and identify critical boundaries in competitive-balance space, partitioning it into distinct regions where only a given subset of species can survive. Fig. 3b shows that full coexistence is possible only within a limited region that encloses the equal-balance condition.

A key insight is that equal competitive balance does not imply that species have identical traits. Rather, it reflects a global trade-off at the community level. Indeed, even in a simulation where species are assigned identical competitive balances but differ in their traits, they exhibit distinct abundance distributions and, in particular, mean abundances (Fig. 3c). This demonstrates that competitive balance is not trivially linked to mean abundance and it captures a fundamental ecological constraint instead.

In real metacommunities, a perfect match of competitive balances is neither expected to be possible nor is it required for coexistence. Our theory predicts that a group of competing species can coexist within the same metacommunity as long as their competitive balances are not too dissimilar. Remarkably, this prediction aligns closely with observations from empirical data (Fig. 3d–f). Although the empirical effective dispersal strength and dispersal heterogeneity extracted from the VAA distributions have a large spread—reflecting the high variability in the parameters describing effective dispersal strategies among species—the situation is strikingly different for their competitive balances. Their values are tightly clustered around the mean, despite the broad range of individual species parameters. In other words, the competitive balances inferred for all species in the forest communities we analyse are closely aligned—and it is this narrow distribution that enables their widespread coexistence.

### Species vulnerability as a distance in competitive balance space

**Fig. 4:**
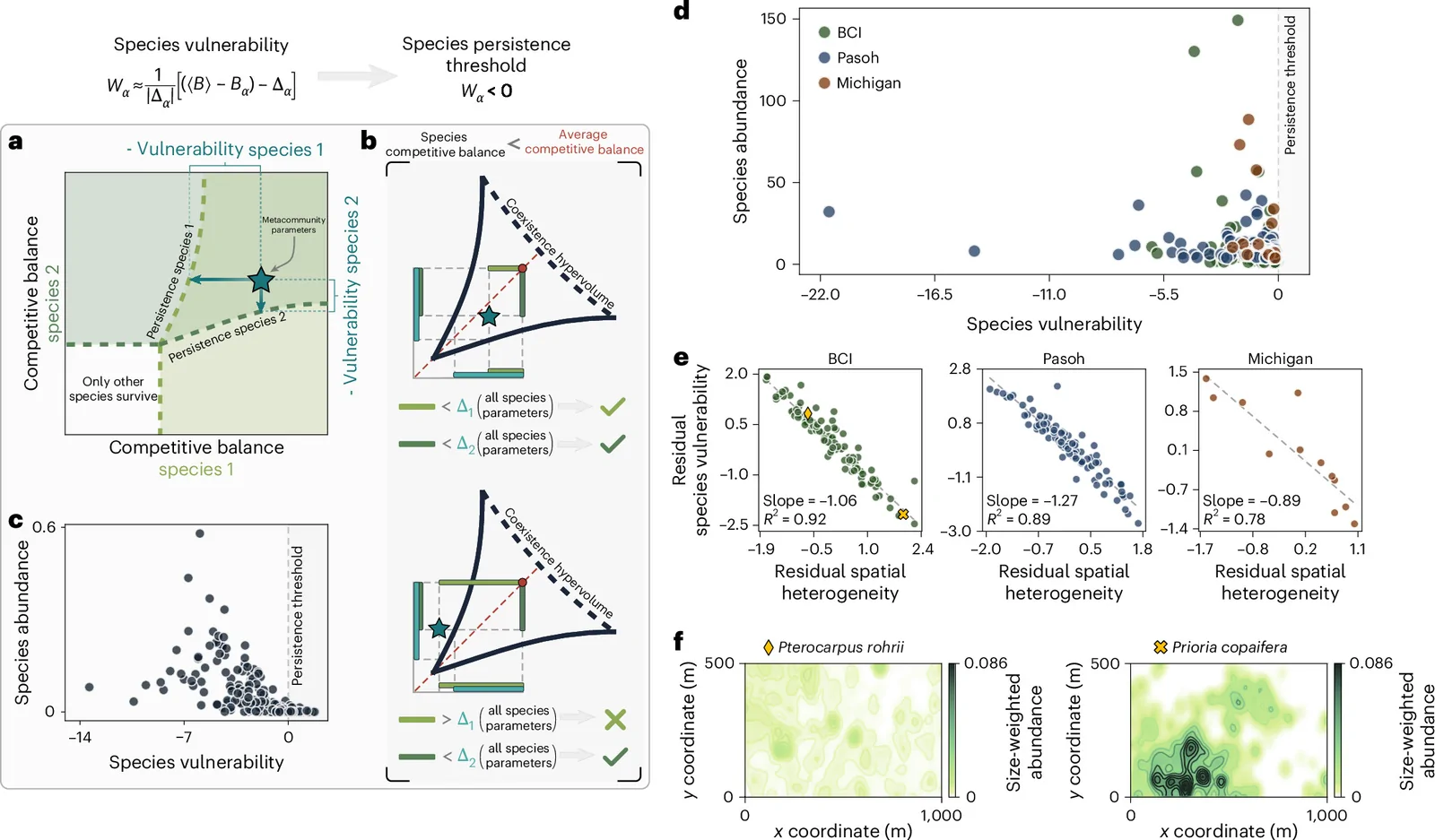
Distance to persistence threshold within the competitive balance space defines vulnerability. The vulnerability found has no trivial relation to abundance and is strongly influenced by dispersal heterogeneity. **a**, The star marks the position of the community in competitive balance space, here projected onto a two-species subspace. Dashed lines indicate species-specific critical boundaries, below which that species cannot persist in the community. The distance of a species from the boundary quantifies its vulnerability taken with a negative sign if above the persistence threshold, as indicated by the minus sign in ‘vulnerability species 1/2’. **b**, Schematic of practical vulnerability computation. The red circle marks the community average along the equal-balance line, serving as a baseline for perturbation analysis. The projection of the distance from the position of the community to the red circle onto the axis (green line) of species 1 determines persistence: if this projection is smaller than Δ_*α*_, the approximate distance from the circle to the critical boundary (cyan line), the species survives. In the top panel, both species persist in the larger metacommunity; in the bottom panel, species 1 is below the persistence threshold. **c**, Simulated metacommunity: stationary average abundance versus species vulnerability for randomly parameterized communities (Methods). **d**, Scatter plot of species vulnerability versus abundance for the three forest datasets. All species exhibit negative vulnerability (that is, persistence) and abundances span a broad range except near threshold. **e**, Partial correlation (controlling for shared correlations with mean abundance, Methods) between ${\sigma }_{{p}_{\alpha }}$, the spatial heterogeneity of the space-use distribution, and *W*_*α*_ of all forest datasets at *L* = 50 m. The significant negative correlation between residuals indicates that, at fixed mean abundance, greater spatial variability reduces the vulnerability of a species to competition. The two yellow markers indicate two representative species that show comparable total abundances but different spatial heterogeneity. **f**, Spatial size-weighted distribution for the two representative species of the BCI forest. For *Prioria copaifera*, the spatial heterogeneity is high (${\sigma }_{{p}_{\alpha }}=0.038$), whereas for *Pterocarpus rohrii* the spatial heterogeneity is lower (${\sigma }_{{p}_{\alpha }}=0.003$). Their total stem-count is similar (1,348 versus 1,380).

When the competitive balances of species in a metacommunity diverge too far from each other, species with lower competitive balance may not survive long-term competition. Our theory predicts that the vulnerability of a species can be quantified in terms of how close it is to its competitive-exclusion threshold, which depends on the competitive balance of all other species. In principle, our theory allows to compute the extinction boundaries for each species as the competitive balance below which the species cannot survive in the community. Then, the distance from the position of the metacommunity in the competitive balance space to its own persistence boundary provides a metric of how vulnerable a species is to competitive exclusion. We denote the vulnerability of species *α* with *W*_*α*_, defined such that *W*_*α*_ < 0 indicates that the species can persist in the metacommunity, whereas a positive *W*_*α*_ signals that it will eventually be outcompeted. This idea is illustrated in Fig. 4a for two species.

In practice, however, the computation of vulnerability in this way becomes infeasible in species-rich communities, as the extinction boundaries depend not only on the exact values of the competitive balances of all other species, but also iteratively on whether those species are above or below their own persistence thresholds in subcommunities—resulting in a combinatorial explosion of interdependent critical boundaries. To work around this difficulty, we developed an approximate measure which becomes increasingly accurate as the number of interacting species grows (Methods). We estimate the distance to the persistence boundary of a given species based on a perturbative expansion of the competitive balance using the equal-balance condition as a reference point. The equal-balance condition provides a baseline reference point from which the community competitive balance deviates. If the projection of this deviation onto the axis of a species is greater than Δ_*α*_, the distance between the baseline point and the critical boundary of species *α*, the species cannot survive long-term competition. The core idea is represented graphically in Fig. 4b, where two possible cases are shown—one in which both species can coexist with the community and one in which one of them cannot survive. The explicit expression for the vulnerability of species *α* is given in the Methods and Table 1. The definition explicitly shows that the vulnerability is not determined solely by the traits of a species, rather it depends on the composition of the entire community.

Numerical simulations of the model with randomly chosen species parameters show that species with positive vulnerabilities have a stationary abundance that approaches zero, consistent with the theory (Fig. 4c). Interestingly, we observe that, away from the persistence threshold, there is a substantial spread in the species abundances, suggesting that low abundance does not necessarily imply high vulnerability.

### Less abundant does not always imply more vulnerable

We can now apply our theory to calculate the vulnerability of species in the three forest plots. Consistent with our theoretical predictions, the results for all three datasets show that all species have vulnerability values below the persistence threshold—*W*_*α*_ < 0. This reinforces the conclusion that the observed communities lie within the coexistence region predicted by our theoretical approach and are stable (Fig. 4d). Remarkably, we find that abundance and vulnerability are strongly correlated only for species with vulnerability close to zero, that is, close to the extinction threshold. Far from this threshold, instead, we find a large range for the average abundance of each species, signalling that species with low average abundance are not necessarily at risk of competitive suppression, consistent with the observation made above that competitive balance depends on the entire community and it is thus not necessarily associated to low average abundance. These findings are consistent across all three datasets and are supported by numerical simulations of the model.

Away from the threshold, since average abundance is not a strong predictor of species vulnerability, we ask whether there are systematic relationships between the dispersal strategy of a species, its vulnerability and its mean abundance. Our theoretical analysis (Methods) informs us that species with the same vulnerability and more evenly distributed dispersal kernels have higher average abundances, because the risk of oversaturation is locally reduced. However, for two species with the comparable average abundance, this does not indicate what can explain differences in vulnerabilities. Although our theoretical framework does not allow for directly inverting vulnerability as an explicit function of dispersal heterogeneity, we can analyse this relationship in more detail. Specifically, we examine the partial correlation between *W*_*α*_, the vulnerability of a species and ${\sigma }_{{p}_{\alpha }}$, the standard deviation of the spatial space-use fraction distribution, quantifying the heterogeneity of its spatial distribution. This partial correlation measures the association between these two variables after accounting for their shared dependence on mean abundance (Methods). The results reveal a clear and significant negative correlation between ${\sigma }_{{p}_{\alpha }}$ and *W*_*α*_ in all three datasets (Fig. 4e; detailed statistical analysis is reported in the Supplementary Information, section 8). This indicates that even if two species were to have the same average abundances but different spatial variability, the one with more heterogeneous dispersal would tend to be a superior competitor. Intuitively, heterogeneous dispersal means that—given a fixed colonization budget—there will be habitat regions where a locally strong competitor is difficult to exclude, despite a reduction in overall abundance due to local saturation. This effect is visualized for two example species from the BCI dataset (Fig. 4f) that have comparable total abundance (~2% difference) but exhibit rather different spatial distributions and hence different vulnerabilities. This trade-off between total abundance, abundance variability and vulnerability may represent a key mechanism underlying the coexistence of many species.

## Discussion

We validated a theoretical framework against three forest communities. Using an underlying dynamical metacommunity model, we introduced a set of metrics that can be efficiently inferred from large-scale ecological data. While derived in a specific mathematical framework, these metrics admit model-independent interpretations, indicating that their relevance goes beyond the assumptions of the model. In particular, our work enables intuitive interpretation of the relationships between vulnerability, abundance and dispersal strategies in the metacommunity. These relationships represent non-trivial trade-offs. On the one hand, at a given competitive balance species with a high abundance tend to have less spatially variable dispersal, allowing them to colonize more patches effectively, whereas species with more heterogeneous dispersal patterns (broader kernel distributions) are more subject to crowding effects. On the other hand, at a given abundance, a species with a more spatially variable colonization strategy is less vulnerable, because it can create a spatial niche, from which it is harder to push out, buffering competitive suppression. These insights provide a mechanistic link between dispersal heterogeneity, abundance and survival, illustrating how metacommunity structure emerges from individual-level dispersal strategies.

The idea that trait-derived quantities govern competitive outcomes has a long theoretical history, from resource-ratio theory^54^ to modern trait-based coexistence frameworks^55,56^. Our approach extends this by inferring the relevant trade-off directly from spatial abundance data, without requiring previous trait measurements. In this sense, our measure of competitive balance can be viewed as a community-wide generalization of the competition–colonization trade-off^38,57–60^. Although community-level manifestations of this trade-off have been reported^21,61,62^, their implications for coexistence in heterogeneous landscapes have remained unclear^63^. Here we make this link explicit and show how such trade-offs shape the risk of competitive exclusion for species. Frameworks that characterize coexistence as a feasibility domain in parameter space have enabled empirical applications, but rely on estimates of interaction coefficients or trait-based proxies^12^. In species-rich systems, such information is typically unavailable and incomplete knowledge of niche dimensions can substantially affect the inference of interaction structure^64^. By contrast, the analytical solution of our spatial model integrates dispersal and interaction effects at the community level, requiring only spatial census data for its application. Our vulnerability index therefore provides a flexible, context-dependent measure that accounts for the community composition. That is, a species with low vulnerability in one metacommunity may be at risk in another, depending on both species composition and spatial structure.

This context-dependent nature of our metrics could make our work suitable for several extensions. For instance, the introduction of an alien species with a specific competitive balance would lead to different coexistence or extinction scenarios of the entire community, depending on its relative position in the trade-off spectrum compared with the native community. This dynamic reconfiguration of species persistence underscores the interaction-dependent nature of the stability of a community. More generally, the framework could be extended to investigate hypothetical ecological disturbances, such as the selective suppression of a single species, tracking how the vulnerability of the remaining species is altered and whether cascading exclusions analogous to a domino effect would emerge. These applications indicate how the framework may be used to probe the resilience and fragility of ecological networks under perturbations. The potential to extract information on temporal dynamics of biodiversity from spatial distributions of terrestrial species was already observed empirically^32^. Our work provides a solid theoretical foundation for this type of inference.

Our results have limitations. One is that our vulnerability metric does not provide information on the timescales over which different species approach their steady-state, but only predicts the final outcome. In our numerical simulations, we observed that these timescales are highly dependent on species-specific parameters, with some species stabilizing faster than others. This discrepancy suggests that transient dynamics, which are not captured by the vulnerability index, are crucial in shaping short- and medium-term ecological interactions. Understanding the factors that drive these variations in transient behaviour is needed^65,66^.

Closely related to this, at very low abundances, demographic fluctuations will become important. Recent work has highlighted the need to introduce probabilistic frameworks into coexistence theory^10^. In this context, future work could include incorporating stochastic effects and accounting for temporal fluctuations^17,52^ to move beyond deterministic proximity to exclusion, and also exploring whether these can be quantitatively linked to the spatial buffering effect described here.

A further limitation is that distinct mechanisms of local competition and facilitation cannot be disentangled in our framework. At the level of description provided by our model, both contribute by amplifying or buffering the inferred kernel parameters. As recent works have highlighted the role of facilitation in driving coexistence^8,46,56,67^, extending the framework to resolve facilitative interactions through pairwise or trait-mediated effects^68,69^ is also needed. This extension is particularly relevant in systems where such interactions have a stronger or more structured role and would probably require going beyond the analytically tractable formulation of the model.

Overall, our study provides a tractable approach for studying metacommunity dynamics by bridging ecological theory and statistical physics and provides a new perspective on the fundamental principles governing biodiversity patterns and their resilience to perturbations.

## Methods

### Model

Our theory is based on a mathematical model describing the temporal dynamics of *S* species inhabiting a landscape of *N* sites, each with a finite amount of available space. The model tracks how the fraction of site *i* = 1…*N* occupied by species *α* changes with time. We denote this fraction as *p*_*αi*_. Being defined as a fraction, $0\le \mathop{\sum }\nolimits_{\alpha = 1}^{S}{p}_{\alpha i}\le 1$ for all sites, which also implies 0 ≤ *p*_*αi*_ ≤ 1 for all *i* and *α*. The model dynamics are governed by the following set of differential equations:

1 $$\frac{{\rm{d}}{p}_{\alpha i}(t)}{{\rm{d}}t}=-{e}_{\alpha }{p}_{\alpha i}(t)+\left(1-\mathop{\sum }\limits_{\beta =1}^{S}{p}_{\beta i}(t)\right)\mathop{\sum }\limits_{j=1}^{N}{K}_{\alpha ,ij}\,{p}_{\alpha j}(t).$$

Here *K*_*α*,*ij*_ is a species-specific interaction–colonization kernel of species *α*. Its diagonal elements capture the strength of local growth and competition processes, whereas the off-diagonal kernel elements encode the overall influence of all possible colonization processes through which patch *j* can affect patch *i*, that is the rate at which individuals of species *α* generated in patch *j* can disperse through the environment and eventually colonize patch *i*. The term $(1-\mathop{\sum }\nolimits_{\beta = 1}^{S}{p}_{\beta i})$ represents the free space in patch *i*, enforcing saturation of available space.

The kernel matrix *K*_*α*_ is an effective species-specific interaction–colonization kernel. It quantifies both how species interact and how they disperse between patches. The kernel is not measured directly from field data, nor do we attempt to reconstruct each individual kernel entry *K*_*α*,*ij*_ separately. Doing so would require detailed knowledge of local demographic rates, dispersal between all pairs of patches and interactions in every local environment, which is generally infeasible in species-rich and spatially heterogeneous systems. Instead, we assume that, for each species, the entries of *K*_*α*_ are drawn from a statistical distribution ${{\mathcal{Q}}}_{N}^{(\alpha )}$ characterized by a few species-specific parameters. These parameters capture the variability in interactions and dispersal strategies among species. Although the kernel elements are random, we are able to infer the parameters of ${{\mathcal{Q}}}_{N}^{(\alpha )}$ indirectly from spatial abundance patterns for each species, by exploiting an explicit solution of the model.

At the level of mathematical structure, our equations align with classic metapopulation and metacommunity frameworks^37,38^ and previous work has shown that such dispersal kernels can emerge as deterministic limits of stochastic models describing local birth, death, exploration and colonization processes^39^. To gain insight about the possible ecological processes at play, one can rewrite equation (1) in the following form:

2 $$\frac{{\rm{d}}{p}_{\alpha i}(t)}{{\rm{d}}t}={K}_{\alpha ,ii}{p}_{\alpha i}(t)\left(1-{p}_{\alpha i}(t)\right)$$

3 $$-{e}_{\alpha i}{p}_{\alpha i}(t)$$

4 $$-\mathop{\sum }\limits_{{\beta =1}\atop{ \beta \ne \alpha}}^{S}{p}_{\beta i}(t){K}_{\alpha \beta ,ii}^{{\prime} }\,{p}_{\alpha i}(t)$$

5 $$+\left(1-\mathop{\sum }\limits_{\beta =1}^{S}{p}_{\beta i}(t)\right)\mathop{\sum }\limits_{{j=1}\atop{ j\ne i}}^{N}{K}_{\alpha ,ij}{p}_{\alpha j}(t).$$

Each term on the right side of the equation corresponds to a different process: local logistic growth with rate *K*_*α*,*ii*_ (equation (2)); linear death with rate *e*_*αi*_ (equation (3)); local effective competition term proportional to ${K}_{\alpha \beta ,ii}^{{\prime} }$, which should be understood as the difference between competition and reduction of competition due to facilitation between species (equation (4)); and colonization, which depends on local space availability and dispersal from other sites with rate *K*_*α*,*ij*_ (equation (5)). Under two simplifying assumptions—(1) the local death rate is patch-independent (*e*_*αi*_ ≡ *e*_*α*_) and (2) the local competition term is independent of the invading species and equals the local growth rate ${K}_{\alpha \beta ,ii}^{{\prime} }\equiv {K}_{\alpha ,ii}$ —the equations reduce to equation (1).

We also note in passing that recasting our model in terms of a spatial mean-field analysis would yield a mathematical structure that has some resemblance to that of classical hierarchical competition models^70^. In our model, however, we do not assume a competition hierarchy a priori. Instead, coexistence trade-offs emerge from the interplay between model dynamics and quenched randomness. To uncover such emergent trade-offs, a mean-field analysis is not adequate. Instead, we resort to a dynamical mean-field approach, as explained next.

#### DMFT

In real ecosystems, estimating the colonization kernel directly is challenging. One simplification is to assume that in large, heterogeneous environments lacking macroscopic barriers, the kernel elements are randomly distributed, mirroring the underlying landscape heterogeneity. We adopt the assumption of ‘homogeneous inhomogeneity’, for which all patches are statistically equivalent. Hence, we assume that the kernel elements of each species are realizations of a random variable *K*^(*α*)^ with probability distribution ${{\mathcal{Q}}}_{N}^{(\alpha )}$. Under this framework, we make the following scaling assumptions for the characteristic function of the kernel distribution in the limit of a large number of sites *N* → *∞*:

6 $$\int\,{\rm{d}}K{e}^{-iKz}{{\mathcal{Q}}}_{N}^{(\alpha )}(K)={e}^{{F}^{(\alpha )}(z)/N}.$$

With this assumption, the full system reduces to a set of *S* effective stochastic equations, one for each species^40^:

7 $$\frac{{\rm{d}}{p}_{\alpha }(t)}{{\rm{d}}t}=-{e}_{\alpha }{p}_{\alpha }(t)+\left(1-\mathop{\sum }\limits_{\beta =1}^{S}{p}_{\beta }(t)\right){\eta }_{\alpha }(t),$$

where *η*_*α*_(*t*) is a stochastic process, the properties of which summarize the dynamics of the coupled system in one representative patch. This approach represents a non-Gaussian generalization of the usual DMFT^40^. The generalized DMFT yields the following equations relating the statistical properties of *η*_*α*_(*t*) to those of *p*_*α*_(*t*) and the colonization kernel *K*:

8 $${\left\langle \mathop{\prod }\limits_{l = 1}^{L}{\eta }_{\alpha }({t}_{l})\right\rangle }_{C}=N{\left\langle {K}_{\alpha }^{L}\right\rangle }_{C}\left\langle \mathop{\prod }\limits_{l=1}^{L}{p}_{\alpha }({t}_{l})\right\rangle ,$$

where 〈 〉_*C*_ indicates an *L*th order cumulant and 〈 〉 on the right-hand side indicates a moment of order *L*. Because the moments of *p*_*α*_ and the cumulants of *η*_*α*_ are interdependent, equation (8) needs to be solved self-consistently, which is in general a challenging problem.

#### Approximate stationary solution defines VAA

We focus on the long-term behaviour of equation (7) and ask which species will persist. The stationary state $\left\{{p}_{\alpha }^{* }\right\}$ obeys the equation

9 $$0=-{e}_{\alpha }{p}_{\alpha }^{* }+\left(1-\mathop{\sum }\limits_{\beta =1}^{S}{p}_{\beta }^{* }\right){\eta }_{\alpha }^{* },$$

where $\left\{{\eta }_{\alpha }^{* }\right\}$ is the stationary state of *η*_*α*_(*t*). From now on, we will drop the asterisks to reduce notation clutter. We define the VAA discussed in the main text as *x*_*α*_ = *η*_*α*_/*e*_*α*_ and solve equation (9) for it:

10 $${x}_{\alpha }=\frac{{p}_{\alpha }}{1-{\sum }_{\beta }{p}_{\beta }},$$

which captures the ratio of abundance to the amount of free space available. An important property of the VAA derived from the DMFT is that, in the limit of large-system size, its joint distribution is a product of individual distributions across different species^40^, which greatly facilitates its comparison to empirical data.

Assuming that the cumulant generating function of the kernel distribution, *F*^(*α*)^(*z*), is analytic, the definition of *η*_*α*_, the self-consistent noise process in equation (8) is equivalent to the following equation

11 $${F}_{{\eta }_{\alpha }}(z)=\left\langle {F}^{(\alpha )}\left(z{p}_{\alpha }\right)\right\rangle ,$$

where ${F}_{{\eta }_{\alpha }}(z)$ is the cumulant generating function of *η*_*α*_ and angular brackets indicate averaging over realizations of the noise process *η*_*α*_. To make analytical progress, we further assume that the statistical distribution of the kernel *K*_*α*_ can be well approximated by a gamma distribution with species-specific parameters *δ*_*α*_ and *β*_*α*_, as discussed in the main text,

12 $${K}_{\alpha ,ij} \sim {{\mathcal{Q}}}_{N}^{(\alpha )}(K)\equiv \frac{{\beta }_{\alpha }^{\frac{{\delta }_{\alpha }}{N}}}{\Gamma \left(\frac{{\delta }_{\alpha }}{N}\right)}{K}^{-1+\frac{{\delta }_{\alpha }}{N}}{e}^{-{\beta }_{\alpha }K}\Theta (K),$$

where the shape parameters of the gamma distributions, *δ*_*α*_, are scaled with *N* to have a consistent large-system-size limit. Then, assuming that the intensity of the self-consistent noise is not overwhelmingly strong, we can make the approximation 〈*F*^(*α*)^(*zp*_*α*_)〉 ≈ *F*^(*α*)^(*z*〈*p*_*α*_〉), which leads to an explicit solution for the distribution of *x*_*α*_ = *η*_*α*_/*e*_*α*_

13 $${x}_{\alpha ,ij} \sim {{\mathcal{L}}}_{\alpha }(x)\equiv \frac{{\bar{\beta }}_{\alpha }^{{\delta }_{\alpha }}}{\Gamma ({\delta }_{\alpha })}{x}^{-1+{\delta }_{\alpha }}{e}^{-{\bar{\beta }}_{\alpha }x}\Theta (x),$$

where ${\bar{\beta }}_{\alpha }={\beta }_{\alpha }{e}_{\alpha }/\langle {p}_{\alpha }\rangle$ and 〈*p*_*α*_〉 denotes the abundance of species *α* averaged across sites. Consequently, the VAA itself follows a gamma distribution, with parameters distinct from—but related to—those of the colonization kernel via the above relation. We can now explicitly relate ${\bar{\beta }}_{\alpha }$ to the kernel parameters by starting from the following equation for 〈*p*_*α*_〉, obtained by inverting equation (10) and applying the definition of the spatial average:

14 $$\langle {p}_{\alpha }\rangle =\int\,\mathop{\prod }\limits_{\mu =1}^{S}\left[d{x}_{\mu }\,{{\mathcal{L}}}^{(\mu )}({x}_{\mu })\right]\frac{{x}_{\alpha }}{1+{\sum }_{\gamma }{x}_{\gamma }}.$$

Manipulating this last equation yields a system of integral equations that links the set of all ${\{{\bar{\beta }}_{\alpha }\}}_{\alpha = 1\ldots S}$ to the inverse of the competitive balance of species *α*:

15 $${B}_{\alpha }^{-1}=\int_{0}^{\infty }d\lambda \,\frac{{e}^{-\lambda -{\sum }_{\gamma }{\delta }_{\gamma }{\ln}\left(1+\lambda /({\bar{\beta }}_{\gamma })\right)}}{1+\lambda /({\bar{\beta }}_{\alpha })},$$

where the competitive balance itself is defined as

16 $${B}_{\alpha }\equiv \frac{{\delta }_{\alpha }}{{\beta }_{\alpha }{e}_{\alpha }}.$$

We now consider the case in which only one species is present. We set in equation (14) the abundances of all species except *α* to zero and analyse the integral on the right-hand side in the limit 〈*p*_*α*_〉 → 0^+^. We posit that the transition between persistence and extinction is smooth. Through this approach, we find that the transition occurs, that is 〈*p*_*α*_〉 = 0^+^, when the ratio *β*_*α*_*e*_*α*_/*δ*_*α*_ equals unity. In other words, the species can survive in isolation when *B*_*α*_ > 1.

#### (Co)existence transition boundaries

As mentioned above, the persistence condition *B*_*α*_ > 1 holds for a species in isolation. When other species are present, this critical transition to persistence depends on the competitive balance of all species. Specifically, the critical values of ${\{{B}_{{\rm{crit}},\alpha \varphi }\}}_{\phi = 1\ldots S}$ marking the transition of species *α* from local extinction to survival when all other *S* − 1 are present form an *S* − 1 dimensional manifold described by the following parametric equations

17 $${B}_{{\rm{crit}},\alpha \varphi }^{-1}=\int_{0}^{\infty }d\lambda \,{e}^{-\lambda }\mathop{\prod }\limits_{\gamma \ne \alpha }^{S}{\left(1+{t}_{\gamma }\lambda \right)}^{-{\epsilon }_{\gamma \varphi }}$$

18 $${\epsilon }_{\gamma \varphi }=\left\{\begin{array}{ll}{\delta }_{\varphi }+1,\quad &\gamma =\varphi \\ {\delta }_{\gamma },\quad &\gamma \ne \varphi \end{array}\right.$$

where 0 < *t*_*γ*_ < *∞* are the parametric descriptors of the manifold and *γ* = 1, 2…*α* − 1, *α* + 1, …, *S*. These critical boundaries are obtained from equation (15) evaluating the limit $1/{\bar{\beta }}_{\alpha }\to {0}^{+}$, which corresponds to 〈*p*_*α*_〉 → 0. Boundaries in Fig. 3a,b were plotted using equations (17) and (18) in the case *S* = 3, with *B*_3_ = 2, *δ*_1_ = 1.5, *δ*_2_ = 3, *δ*_3_ = 2.

If one or more species are extinct, then they need to be excluded from the product term. If one species goes extinct, there are *S* new possible transition manifolds (each of dimension *S* − 1). Similarly, if two species are extinct, one obtains a further transition manifold for each of the $$\left(\begin{array}{c}S\\ 2\end{array}\right) = \frac{S(S-1)}{2}$$ possible pairs of extinct species. This pattern continues, leading to a combinatorial explosion of possible transitions to track. We note that, in principle, recomputing these transition manifolds iteratively could be used to predict how the removal or introduction of species affects community stability, as discussed in the outlook (‘Discussion’). However, the number of such boundaries grows combinatorially with the number of species *S*, rendering this approach impractical in species-rich ecosystems and motivating the use of the vulnerability index as a scalable alternative.

#### Perturbative expansion leads to vulnerability definition

To circumvent the problem of the combinatorial explosion of transition boundaries, we start from the point in the competitive balance space defined by the condition *B*_*α*_ = *B*_00_, that is the scenario in which the competitive balance of all species are equal to the same value. Then, we consider the case of many species *S* ≫ 1. We define *Z*_*α*_ ≡ 1/*B*_*α*_ for convenience and the rescaled abundance *y*_*α*_ = 〈*p*_*α*_〉*S*, which stays finite in the limit *S* → *∞* as opposed to 〈*p*_*α*_〉, which goes to zero because of the condition $\mathop{\sum }\nolimits_{\beta = 1}^{S}{p}_{\beta }\le 1$. We now make the following expansion in powers of 1/*S*

19 $${Z}_{\alpha }={Z}_{\alpha 0}\left(1-\frac{{Z}_{\alpha 1}}{S}+\ldots \,\right),$$

where *Z*_*α*0_ has been allowed to depend on *α* for generality, but will end up being the equal-balance condition discussed in the main text. Given this ansatz, we also expect the rescaled average abundances to scale as

20 $${y}_{\alpha }={y}_{\alpha 0}\left(1+\frac{{y}_{\alpha 0}}{S}+\ldots \,\right).$$

Then, we substitute equation (19) into the left-hand side of equation (15) and 〈*p*_*α*_〉 = *y*_*α*_/*S* into its right-hand side, which we then expand in powers of 1/*S*. Matching leading order terms yields the following consistency condition for *Z*_*α*0_

21 $${Z}_{\alpha 0}={Z}_{00} < 1,$$

and the following expression for the zero order of *y*_*α*_:

22 $${y}_{\alpha 0}=\frac{{\beta }_{\alpha }{e}_{\alpha }({Z}_{\alpha 1}-\Delta )}{{Z}_{00}};\quad \Delta =\frac{{\left[{\beta }_{\alpha }{e}_{\alpha }{Z}_{\alpha 1}\right]}_{\alpha }-{Z}_{00}\left(1-{Z}_{00}\right)}{{\left[{\beta }_{\alpha }{e}_{\alpha }\right]}_{\alpha }},$$

where ${\left[\, \right]}_{\alpha }$ indicates an average over species. The persistence condition for species *α* is *y*_*α*0_ > 0, which, using equation (19), leads to

23 $${y}_{\alpha 0} > 0\,\iff \,\frac{1}{| \Delta | }\left(\Delta -S\frac{{\left[{Z}_{\alpha }\right]}_{\alpha }-{Z}_{\alpha }}{{\left[{Z}_{\alpha }\right]}_{\alpha }}\right) < 0.$$

In equation (23) we identify ${Z}_{00}={[{Z}_{\alpha }]}_{\alpha }$ as the baseline point for the expansion, as it minimizes the distances from it and is most consistent with the ansatz the expansion is based on. The inequality in equation (23) corresponds to the definition of the vulnerability *W*_*α*_.

To remain consistent with species-specific parameters, we rewrite the vulnerability in terms of the competitive balances of species ${B}_{\alpha }={Z}_{\alpha }^{-1}$ to obtain the intuitive form of the vulnerability discussed in the main text. To this end, we make the approximation ${[1/{B}_{\alpha }]}_{\alpha }\approx 1/{[{B}_{\alpha }]}_{\alpha }$, which holds when *B*_*α*_ are close to each other, as required for coexistence when *S* is large. In this way, we can rearrange equation (23) obtaining

24 $${W}_{\alpha }\approx \frac{1}{| {\Delta }_{\alpha }| }\left(\left({\left[{B}_{\alpha }\right]}_{\alpha }-{B}_{\alpha }\right)-{\Delta }_{\alpha }\right) < 0,$$

where *B*_*α*_ = *δ*_*α*_/*β*_*α*_*e*_*α*_ and Δ_*α*_ = − Δ *B*_*α*_/*S*, thereby connecting vulnerability and species-specific dispersal parameters.

### Numerical simulations

Numerical simulations of the model were carried out by integrating equation (1) using an adaptive Runge–Kutta scheme implemented in custom C++ code based on GSL scientific libraries. Simulations were run until the system reached a stationary state, which was assessed by checking that the maximum change (across species and sites) in local abundance ∣*p*_*αi*_(*t* + 1) − *p*_*αi*_(*t*)∣ fell below a precision threshold *θ* = 10^−5^.

The simulation results shown in Fig. 3a (inset) were obtained by simulating a metacommunity of five species with equal *B*_*α*_ but with *δ*_*α*_ values evenly spaced in the interval (2, 8), progressing to stationarity in a landscape of *N* = 2,000 sites. Only three of the five species are shown for visualization clarity. Results were averaged over 1,000 realizations of the interaction-dispersal kernel *K*. The results shown in Fig. 3b are based on simulations of three species with *δ*_1_ = 1.5, *δ*_2_ = 3, *δ*_3_ = 2 and *B*_3_ = 2, over *N* = 800 sites. The competitive balance between the first two species was systematically varied by changing *β*_1_ and *β*_2_. For each grid point, results were averaged over ten realizations of *K*. Finally, the results shown in Fig. 4c were obtained from 40 realizations of a community of *S* = 6 species distributed over *N* = 1,200 sites, with each species assigned randomly chosen values of *B*_*α*_ and *β*_*α*_. The values of *β*_*α*_ were uniformly distributed in the interval (0.5, 3.0), while *B*_*α*_ values were randomly drawn from intervals centred around either *B*_00_ = 1.25 or *B*_00_ = 1.66, with varying spreads in the range (0.5, 1.0)/*S* to mimic variability in natural communities.

### Forest data analysis

We partition each forest plot into square subplots of side length *L*. For the results presented in the main text, we use *L* = 50 m, selected from a range of spatial scales (10–125 m for BCI and Pasoh and 10–100 m for Michigan, the latter limited by the need to tile its non-rectangular geometry).

For a given spatial scale *L*, we estimate the effective space within patch *i* occupied by species *α*, denoted *a*_*αi*_, using two alternative metrics. The simpler stem-count metric sets *a*_*αi*_ = *n*_*αi*_, the number of individuals, implicitly assuming equal space occupancy. The metric weighted by diameter at breast height (DBH) instead defines ${a}_{\alpha i}={\sum }_{j}{d}_{\alpha i,j}^{4/3},$ where *d*_*αi*,*j*_ is the DBH in centimetres of the *j*th individual of species *α* in subplot *i*. The exponent 4/3 follows from the allometric scaling between trunk diameter and projected crown area, which is well-established both theoretically and empirically^44,45^, so that *a*_*αi*_ approximates the effective canopy space occupied by species *α*. We verified that both metrics yield consistent estimates of the effective per-patch carrying capacity and that converting the DBH-weighted carrying capacity to actual canopy area using allometric scaling coefficients for BCI^45^ yields an estimated number of canopy layers consistent with those reported for humid forests^71,72^ (Supplementary Information, section 1). On the basis of a quantitative goodness-of-fit comparison across spatial scales and metrics (Supplementary Information, section 1.1), we adopt the DBH-weighted metric as the primary abundance measure and *L* = 50 m as the standard spatial scale for all main-text analyses. For model comparison, however, we use the stem-count metric. Incorporating DBH-weighting in the null models would require additional assumptions on the sampling of individual sizes and introduce further arbitrariness, while also increasing the number of parameters. Using stem counts therefore ensures a consistent and fair comparison across models.

Species selection for the analysis was based on two criteria, balancing sufficient abundance and adequate spatial sampling. Starting from all species with at least one recorded alive stem, we retained the most abundant species until their cumulative stem-count abundance reached at least 95% of the total abundance in the plot. We then excluded species present in fewer than 10% of subplots, as their VAA distributions cannot be reliably estimated because of insufficient spatial sampling. This procedure yielded 101 (BCI), 396 (Pasoh) and 12 (Michigan) species at *L* = 50 m.

We define the per-patch space use as *p*_*αi*_ = *a*_*αi*_/*M*, where *M* is a free parameter representing the total carrying capacity of each subplot, assumed to be spatially homogeneous (we investigated the robustness of this assumption in Supplementary Information, section 4). To determine *M*, we scan a coarse grid of candidate values, starting from ${M}_{0}={\mathop{\max }\limits_{i}}\,\{{\sum }_{\beta }{a}_{\beta i}\}+1$, the minimum value ensuring a positive denominator in the VAA and incrementing upwards. We then refine the search around the best coarse candidate using a finer grid. For each candidate *M*, we compute the VAA for all species and patches using

25 $${x}_{\alpha i}=\frac{{p}_{\alpha i}}{1-{\sum }_{\beta }{p}_{\beta i}},$$

and fit, independently for each species, the empirical cumulative distribution of non-zero VAA values to the theoretical gamma distribution, equation (13), via least squares. The optimal *M* is selected by minimizing the total CDF mean-squared error across all selected species. This procedure is applied for both the stem-count and DBH-weighted metrics.

#### Species-specific dispersal parameters

After determining the optimal value of *M*, we fitted the VAA distributions of each species at all levels of coarse-graining using equation (13). This allowed us to obtain the values of *δ*_*α*_ and ${\bar{\beta }}_{\alpha }$. A detailed goodness-of-fit analysis for all >500 species at all spatial scales is presented in the Supplementary Information, together with visualization for 212 species (100 + 100 + 12 from the three datasets). In the main text and in Fig. 3, we refer to ${\delta }_{\alpha }^{-1}$ as dispersal heterogeneity because the shape parameter of the kernel distribution determines the heterogeneity of the dispersal matrix—the coefficient of variation of the gamma distribution is $\sqrt{{\delta }^{-1}}$. In the same figure and context, we refer to ${\bar{\beta }}_{\alpha }^{-1}$ as effective dispersal strength reflecting the relation ${\bar{\beta }}_{\alpha }^{-1}=\langle {p}_{\alpha }\rangle {\beta }_{\alpha }^{-1}$, which shows that ${\bar{\beta }}_{\alpha }^{-1}$ is proportional to the scale of the interaction-dispersal kernel distribution, multiplied by the species average abundance, thus motivating its name. Finally, we used equation (15) to compute the competitive balance of each species, from which the value of *β*_*α*_ was obtained. Predictive accuracy was assessed by out-of-sample cross-validation, as described in the next subsection. We used equation (24) to compute the species vulnerability shown in Fig. 4c,d.

#### Out-of-sample predictive accuracy

To assess whether the theoretical prediction for the mean patch occupancy 〈*p*_*α*_〉 generalizes to unseen data, we performed a spatial cross-validation across all three datasets. For each of 20 independent replicates, we randomly split the subplots of each plot into two equal halves. We fitted the model on the training half, obtaining the species-specific parameters ${\delta }_{\alpha }^{{\rm{tr}}}$, ${\bar{\beta }}_{\alpha }^{{\rm{tr}}}$ and the global carrying capacity ${M}^{{\rm{tr}}}$, as described above. Using these parameters, we computed the theoretical mean patch occupancy as ${\langle {p}_{\alpha }\rangle }^{{\rm{th}}}=({\delta }_{\alpha }^{{\rm{tr}}}\,{B}_{\alpha }/{\bar{\beta }}_{\alpha }^{{\rm{tr}}})\,(1-{\pi }_{0,\alpha }),$ where *B*_*α*_ is the competitive balance integral equation (15), evaluated using the training-half parameters for all selected species and (1 − *π*_0,*α*_) is the fraction of non-zero patches, accounting for the absence of probability mass at zero in the gamma distribution. Results are summarized by taking the median across the 20 splits. The comparison is shown in Fig. 2b. Additional out-of-sample validation analyses of the inferred parameters are reported in the Supplementary Information, section  2.

#### Model comparison with negative binomial nulls

We benchmarked our framework against a family of negative binomial (NegBin) null models that reproduce overdispersed abundance fluctuations but assume species independence and no spatial coupling^47,48,73^. We considered three variants of increasing parsimony: (1) a species-specific NegBin, with independent mean and dispersion parameters (*μ*_*α*_, *r*_*α*_) for each species; (2) an intermediate model with species-specific means *μ*_*α*_ and a shared dispersion parameter *r*; and (3) a fully neutral model with both *μ* and *r* shared across species. Including the effective community capacity parameter, these models comprise 2*S* + 1, *S* + 2 and three parameters, respectively, compared with 2*S* + 1 parameters in our DMFT framework. Parameters were estimated by maximum likelihood (Supplementary Information, section 6).

To ensure a consistent comparison, all models were evaluated in a common rescaled abundance space *x*_*s*_. For each fitted model, we generated synthetic datasets matching the empirical dimensions and computed the KS statistic between the observed and simulated *x*_*s*_ distributions (restricted to positive values). Predictions from our model were sampled directly in *x*_*s*_, whereas NegBin samples were generated in count space and transformed using the same rescaling as the data. KS statistics were obtained over repeated synthetic realizations and summarized across species, providing a relative measure of goodness-of fit that enables direct comparison across model classes. Because the rescaling depends on an effective community capacity, this parameter was estimated independently for each model to avoid circularity. In particular, the NegBin models were assigned their own capacity parameter, inferred by minimizing discrepancies between empirical and model cumulative distributions, ensuring that each model was evaluated under its optimal scaling.

In addition to this relative comparison, we assessed absolute goodness-of fit using a Cramér–von Mises–type statistic computed directly from empirical distributions. For each species, we measured the mean-squared difference between the empirical CDF of positive abundances and the fitted model CDF, scaled by the number of observations; under a well-specified continuous model this quantity has an expected value of 1/6, providing a reference scale for interpretation. This metric does not rely on synthetic sampling and complements the KS-based comparison. All analyses were performed on stem-count data to ensure consistency with the discrete NegBin formulation. Further details are provided in the Supplementary Information.

#### Functional guild analysis of VAA parameters

To assess whether the VAA parameters reflect differences in life-history strategies, we compared species-specific parameter estimates across functional guilds in the BCI dataset. Guild assignments (shade-tolerant, gap-exploiting and intermediate) were taken from ref. ^49^. Statistical comparisons were performed using two-sided Mann–Whitney *U*-tests between guilds. To increase statistical power, we focused on an aggregated comparison between gap-exploiting and intermediate species versus shade-tolerant species, while also reporting the full three-guild analysis in the Supplementary Information. *P* values were corrected for several comparisons using the Holm step-down procedure. Parameter distributions were summarized using medians and interquartile ranges and all analyses were performed at the species level. Further details on sample sizes, full pairwise comparisons and robustness of the results are provided in the Supplementary Information.

#### Statistical analysis of vulnerability versus spatial heterogeneity

In the main text, we present the partial correlation between ${\sigma }_{{p}_{\alpha }}$ and *W*_*α*_, while controlling for the effect of the mean abundance of each species. To this, we first performed a linear regression of the log-transformed (−*W*_*α*_) against 〈*n*_*α*_〉 and another of ${\ln} ({\sigma }_{{p}_{\alpha }})$ against ln(〈*n*_*α*_〉). We then regressed the residuals of these two models, denoted ${e}_{{W}_{\alpha }}$ and ${e}_{{\sigma }_{\alpha }}$, respectively, to quantify the residual correlation between *W*_*α*_ and ${\sigma }_{{p}_{\alpha }}$ not explained by the control variable (the mean abundance). All regressions were conducted using the open-source Python library statsmodels, relevant code is provided in the code capsule^74^. Figures and full details of this statistical analysis, for all three datasets and the model, are provided in the Supplementary Information, section 8.

### Software

All analyses were performed in Python 3.12, using numpy 1.26.4 for array operations, scipy 1.13.1 for numerical integration, optimization and statistical distributions, pandas 2.2.2 for data handling, statsmodels 0.14.2 for the regression and partial-correlation analyses and matplotlib 3.8.4 for figure generation. Version-pinned requirements and the complete analysis pipeline are provided in the Code Ocean capsule^74^.

### Reporting summary

Further information on research design is available in the Nature Portfolio Reporting Summary linked to this article.

## Supplementary information

Supplementary Information Supplementary sections 1–8, Figs. 1–18 and Tables 1 and 2.

Reporting Summary

Peer Review File

## Data Availability

The minimum dataset required to reproduce all results of this study is provided within the Code Ocean capsule^74^.

## Code availability

All code required to reproduce the analyses and figures in this paper is provided in the Code Ocean capsule^74^. A user-friendly interface to the parameter extraction and vulnerability metric computation, implementing the core calculation described here, is additionally available as the open-source vulntool package via GitHub at https://github.com/davideb321/vulntool.
