# Supplementary material for: Dispersal diversity buffers species vulnerability to local extinction
Source: Nat Ecol Evol. 2026 Oct 9;10(11):2060–73. doi: 10.1038/s41559-026-03195-y (PMC13736521; doi:10.1038/s41559-026-03195-y)
Supplement: Supplementary file 1 [file 41559_2026_3195_MOESM1_ESM.pdf]

nature ecology & evolution

Supplementary information https://doi.org/10.1038/s41559-026-03195-y

Dispersal diversity buffers species vulnerability to local extinction

In the format provided by the authors and unedited

Supplementary Information

Dispersal diversity buffers species vulnerability to local extinction

Davide Bernardi[1], Giorgio Nicoletti, Prajwal Padmanabha, Samir Suweis, Sandro Azaele, Simon A. Levin, Andrea Rinaldo, Amos Maritan

The following supplementary analyses collectively extend and support the findings presented in the main text by providing detailed visualizations, model and metric comparisons, robustness checks, and statistical analyses for all analyzed species and datasets at different spatial scales.

1 Vacancy-adjusted abundance fitting and metric selection

We recall here for completeness the definitions and fitting procedures used for the vacancy-adjusted abundance (VAA) explained in the main manuscript.

We partition each forest plot into square subplots of side length L. For the main analysis, we use $L = 50$ m, selected from a range of spatial scales (10–125 m for BCI and Pasoh; 10–100 m for Michigan, limited by the plot geometry) based on a quantitative goodness-of-fit analysis (see sec. 1.1 below). For each patch i and species α, we estimate the effective occupied space $a_{\alpha i}$ using two alternative metrics. The simpler *stem-count* metric sets $a_{\alpha i} = n_{\alpha i}$, the number of alive individuals, implicitly assuming equal space occupancy. The *DBH-weighted* metric instead defines

$$a_{\alpha i} = \sum_j d_{\alpha ij}^{4/3}, \tag{S1}$$

where $d_{\alpha ij}$ is the diameter at breast height (DBH, in mm) of the j-th individual. The exponent 4/3 follows from the allometric scaling between trunk diameter and projected crown area [1, 2], so that $a_{\alpha i}$ approximates the effective canopy space occupied by species α. Based on the goodness-of-fit comparison detailed below, we adopted the DBH-weighted metric as the primary abundance measure, but we also used the stem-count metric where appropriate (for instance, for the results presented in section 6).

We define the relative space-use as $p_{\alpha i} = a_{\alpha i}/M$, where M is a shared carrying capacity representing the total available effective (canopy) space per patch. The vacancy-adjusted abundance (VAA) is then

$$x_{\alpha i} = \frac{p_{\alpha i}}{1 - \sum_\beta p_{\beta i}}, \tag{S2}$$

i.e. the ratio of a species' relative occupation to the remaining free space in the patch.

The carrying capacity M is inferred from the data. We scan a grid of candidate values, starting from $M_0 = \max_i \left\{ \sum_\beta a_{\beta i} \right\} + 1$, which represents the minimum value for the carrying capacity ensuring a physically meaningful solution, and refine the search around the best candidate. For each M, we compute the VAA values and fit, independently for each species, the empirical cumulative distribution of positive VAA values to the theoretical Gamma distribution via least squares. The optimal M is selected by minimizing the total CDF mean-squared error across all species.

We excluded species with extremely low abundance or a very high fraction of zero-count patches, as these indicate insufficient statistical or spatial sampling. Species are selected in two steps. First, species are ranked by total stem abundance, and the most abundant species are retained until their cumulative abundance reaches 95% of the community total. Second, species present in fewer than 10% of patches are excluded, as their VAA distributions cannot be reliably estimated from positive samples. This yields 101, 396, and 12 species for BCI, Pasoh, and Michigan, respectively, at $L = 50$ m.

[1] davide.bernardi@unipd.it

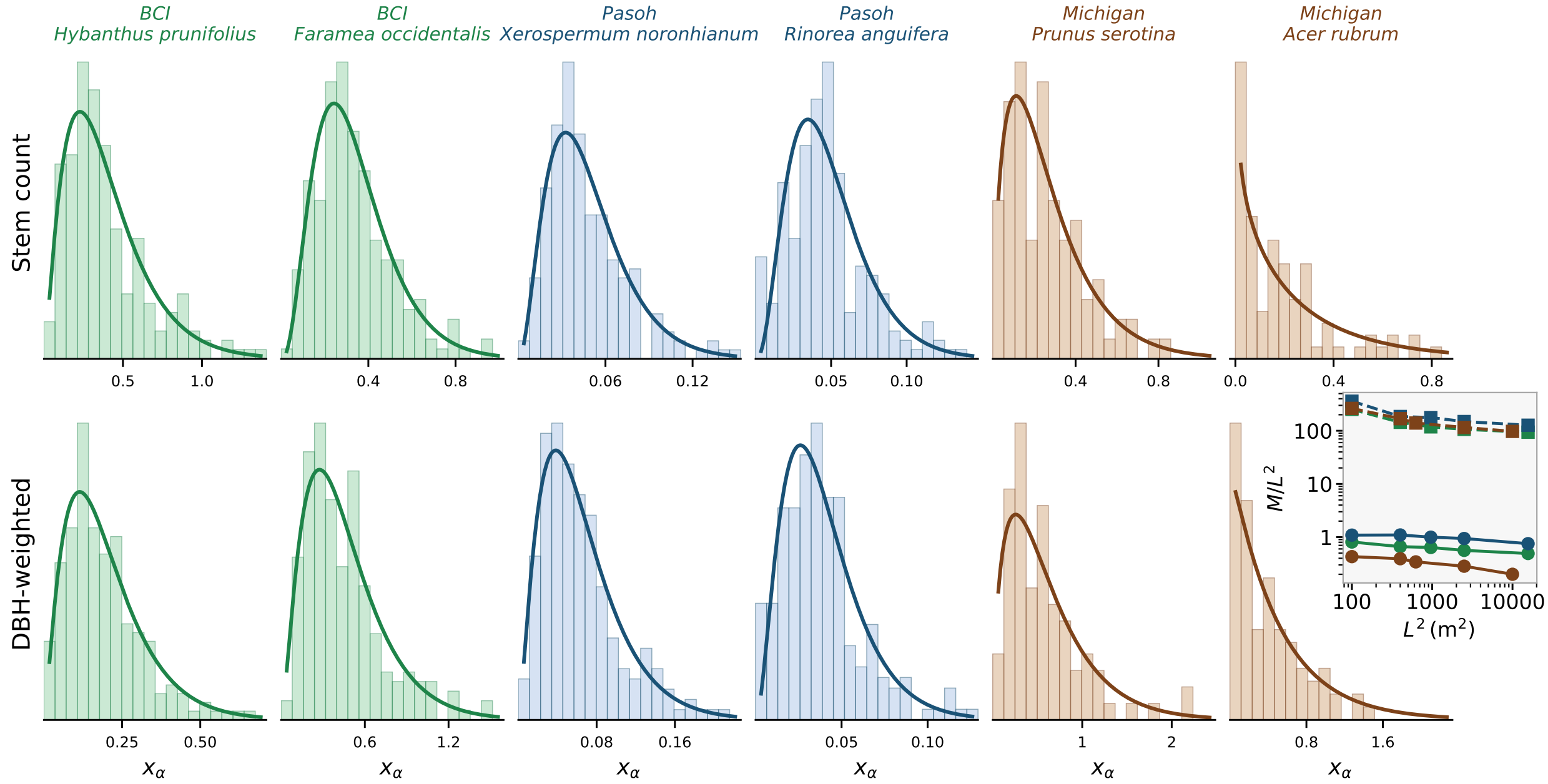

Supplementary Figure 1: **Direct comparison of stem-count and DBH-weighted VAAD fits for representative species at $L = 50\,\text{m}$.** Each column shows one species (two species per dataset); rows correspond to the two abundance metrics. Panel headers give the dataset name and species scientific name. Histograms show the empirical distribution of the vacancy-adjusted abundance x_α across all patches; the curve shows the fitted Gamma distribution. Colours identify datasets: green (BCI), blue (Pasoh), brown (Michigan). Inset: fitted carrying capacity density M/L^2 as a function of patch area L^2 for all three datasets and both metrics (solid circles: stem count; open squares: DBH-weighted); both axes are logarithmic.

We now compare the results obtained with the two metrics, both qualitatively and quantitatively. Supplementary Figure 1 shows a direct comparison of the stem-count and DBH-weighted VAAD fits for the two most abundant species in each dataset at $L = 50\,\text{m}$. Visual inspection shows that both metrics yield good fits across all datasets and species, and also broadly similar shapes.

Before moving on to a more quantitative and comprehensive comparison of the two metrics, we first make a physical consistency check of the DBH-weighting leading to the effective crown area proxy we used for the fits. The allometric relation between trunk size and crown diameter, $A_{\text{crown}} \approx a_0\, d^{4/3}$, involves a forest-specific constant that cannot be determined without independent measurements. However, the total effective crown area should scale as L^2 for all datasets, $M \propto L^2$ (patch capacity should scale with patch area regardless of the forest). Hence, the ratio M/L^2 should be the dataset-specific scaling prefactor. The inset of Supplementary Figure 1 shows that the capacity density M/L^2 is indeed approximately constant within each dataset (flat curves in L), with significant fluctuation only at the smallest spatial scale, which is expected to carry the strongest sampling noise. Note that the two curves have different units, with with the DBH-weighted density M_c/L^2 lying above the stem-count density M_s/L^2. Densities differ across datasets, encoding ecosystem-specific canopy structures and tree-density. The consistent scaling with L^2 of both total per patch carrying capacity means that also the ratio M_c/M_s (effective crown equivalent per stem) is approximately scale-independent within each dataset. The values at $L = 50\,\text{m}$ are 189 (BCI), 158 (Pasoh), and 406 (Michigan) $\text{mm}^{4/3}\,\text{stem}^{-1}$, with similar values for the two tropical forests and with Michigan roughly twice as large, reflecting the substantially larger average tree size in temperate forests.

For completeness, we also report the fitted carrying capacities themselves and an absolute physical consistency check. Supplementary Table 1 lists M_c and M_s at $L = 50\,\text{m}$ for all three datasets, together with the ratio M_s to the stem count of the most densely occupied patch: in all three datasets M_s exceeds this maximum, as required for a physically valid denominator in eq. (S2). To convert the DBH-weighted capacity density M_c into an absolute canopy-area capacity, we use the community-level crown-area allometry relation $A_{\text{crown}} \approx 0.66\, d^{1.34}$ (where $a_0 = 0.66$ is the allometric prefactor when crown area is in m^2, and trunk diameter d in cm) reported for BCI by [2]. Dividing by the patch ground area L^2 gives an estimated number of stacked canopy layers n_{layer} (Supplementary Table 1), which comes out to 3-5 across all three datasets. This is in good agreement with the classical division of humid forests into about four vertical strata (shrub, understory, midstory, canopy), reported by [3] and confirmed quantitatively from height-based clustering of BCI tree species by [4]. Note that the n_{layer} estimates for Pasoh and Michigan (marked † in Supplementary

Table 1) are obtained by reusing the BCI-derived a_0. These two estimates should therefore be read as more qualitative than the BCI one, though the fact that they still come out physically reasonable (matching the same four-layer stratification) is reassuring.

	BCI	Pasoh	Michigan
M_c (crown metric, mm$^{4/3}$)	265,856	372,066	288,468
M_s (stem-count metric, stems)	1,408	2,355	710
M_c/M_s (mm$^{4/3}$ stem^{-1})	189	158	406
M_s / max. patch stem count	1.103	1.004	1.129
Est. canopy layers n_{layer}	3.3	4.6†	3.5†

Supplementary Table 1: **Fitted carrying capacities and physical consistency checks at $L = 50$ m.** M_c and M_s: fitted carrying capacity for the DBH-weighted (crown) and stem-count metrics, respectively. M_s / max. patch stem count: ratio of M_s to the largest total stem count observed in any patch, restricted to the selected species. n_{layer}: estimated number of canopy layers, obtained as $n_{\text{layer}} = a_0 10^{-\frac{4}{3}} M_c/L^2$, where the factor $10^{-\frac{4}{3}}$ is needed to match the units of the allometric prefactor given by [2] (see text). †The allometric prefactor a_0 taken from [2] is for BCI; the Pasoh and Michigan values of n_{layer} reuse this BCI-derived a_0 and are therefore less reliable.

1.1 Selection of the default spatial scale and preferred metric

The choice of the patch side length L used to coarse-grain the data involves a trade-off between two opposing effects. At small L, the total number of patches or subplots used to constructu the VAA, $N = A/L^2$, is large (here A is the total plot area), but finite-size effects and sampling noise are strongest. At large L, each patch aggregates many individuals, smoothing spatial structure and reducing the effective number of independent observations. Hence, the choice of the optimal patch side length L to coarse-grain data and evaluate the VAAD fits involves a trade-off between two opposing effects. To determine both the preferred spatial scale and the preferred abundance metric, we used two complementary statistics: one to assess absolute goodness-of-fit across scales, and one to compare the two metrics directly.

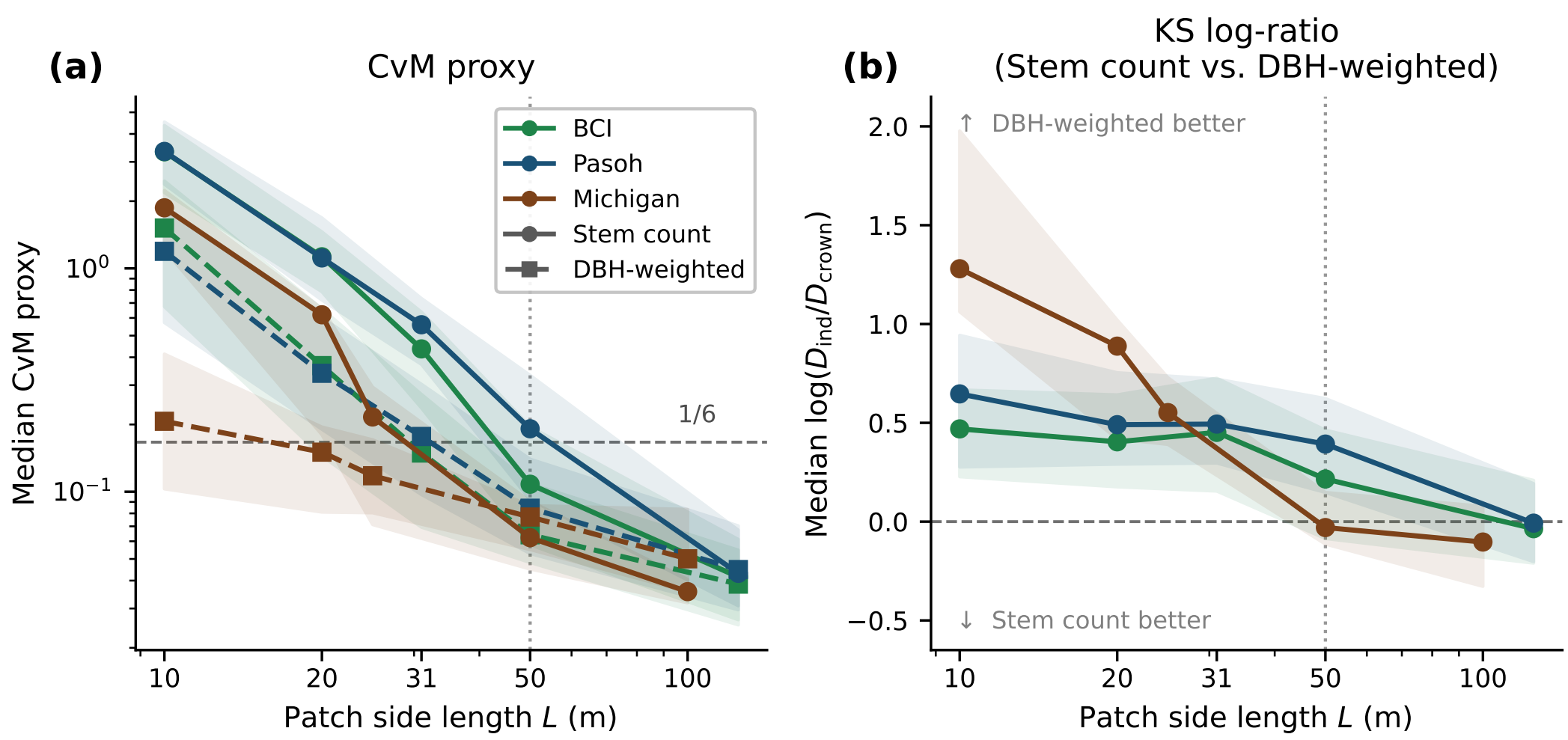

Supplementary Figure 2: **Comparison of fit quality for stem-count vs. DBH-weighted metrics across spatial scales for all three datasets.** Colours identify datasets: green (BCI), blue (Pasoh), brown (Michigan). The dotted vertical line marks $L = 50$ m, selected as primary analysis scale. **(a)**: $n_{\alpha,\text{pos}} \times$ CDF-MSE, a proxy for the Cramér–von Mises criterion (log scale; solid lines with circles: stem-count metric; dashed lines with squares: DBH-weighted metric). Lines show the per-species median; shaded bands show the inter-quartile range (IQR) over retained species. The dashed horizontal line marks the null expectation of 1/6 under a correctly specified model. The CvM proxy drops below 1/6 by $L = 50$ m in all datasets and for both metrics, indicating adequate fit quality at this scale. **(b)**: Per-species median log-ratio of KS statistics, $\log(D_{\text{ind}}/D_{\text{crown}})$, where D_{ind} and D_{crown} are the one-sample KS statistics against the fitted Gamma CDF for the stem-count and DBH-weighted metrics respectively, computed for the same species and same positive-occupancy observations. Positive values indicate better fit for the DBH-weighted metric.

- Cramér–von Mises (CvM)–type statistic. For each species α, we computed the mean squared difference

between the empirical CDF of the positive VAA values and the fitted Gamma CDF (CDF-MSE), and multiplied it by the number of positive observations $n_{\alpha,\text{pos}}$. This quantity is proportional to the Cramér–von Mises statistic and, under a correctly specified continuous model with fixed parameters, has a non-degenerate limiting distribution, making it approximately comparable across sample sizes. Its expected value converges to 1/6, which we use as a reference scale. The unscaled CDF-MSE decreases with $n_{\alpha,\text{pos}}$ even under model misspecification (more patches $\Rightarrow$ smoother empirical CDF $\Rightarrow$ smaller MSE). Multiplying by $n_{\alpha,\text{pos}}$ mitigates this dependence and provides a more appropriate scale-free comparator across spatial resolutions. Because parameters are estimated from the same data, the null distribution is not exactly that of the classical CvM statistic. Accordingly, we use this quantity for comparisons across scales and metrics rather than for formal hypothesis testing.

- Relative goodness-of-fit via KS statistic. To compare the two abundance metrics directly, we computed the log-ratio of one-sample Kolmogorov–Smirnov statistics, $\log(D_{\text{ind}}/D_{\text{crown}})$, where D_{ind} and D_{crown} are the KS statistics for the stem-count and DBH-weighted fits, respectively. Because both statistics are computed from the same number of observations $n_{\alpha,\text{pos}}$, the ratio cancels sample-size dependence. Positive values indicate a better fit for the DBH-weighted metric, while a value of zero indicates equal performance.

We evaluated both the stem-count and DBH-weighted metrics at each L; results for all three datasets (BCI, Pasoh, Michigan) are shown in Supplementary Figure 2.

Scale selection. The CvM proxy decreases with increasing L across all datasets and both metrics (Supplementary Figure 2a). The most pronounced improvement occurs between $L = 10$ and $L = 50\,\text{m}$. At $L = 50\,\text{m}$, the CvM drops below the reference value of 1/6 in all cases, indicating that the model provides an adequate statistical description of the data at this scale. Beyond $L = 50\,\text{m}$, the CvM continues to decrease, but the number of patches falls rapidly ($N \leq 32$ for BCI and Pasoh at $L = 125\,\text{m}$; $N = 23$ for Michigan at $L = 100\,\text{m}$), so low values likely reflect reduced statistical power rather than genuine improvement. We therefore adopt $L = 50\,\text{m}$ as the default spatial scale: it is the smallest scale at which the model reaches the expected goodness-of-fit, while still providing a sufficient number of patches for reliable parameter estimation. From a theoretical perspective, this choice is also well motivated: $L = 50\,\text{m}$ is large enough to sample the local community adequately, yet small enough to retain spatial structure relevant to dispersal processes.

Metric selection. The CvM statistic for the DBH-weighted metric is lower than that of the stem-count metric at all spatial scales for BCI and Pasoh, and at all scales below $L = 50\,\text{m}$ for Michigan. To compare the two metrics more directly, we consider the log KS ratio, $\log(D_{\text{ind}}/D_{\text{crown}})$. This quantity is consistently positive across all datasets and spatial scales where statistical power is sufficient ($L \leq 50\,\text{m}$ for BCI and Pasoh, $L \leq 31\,\text{m}$ for Michigan; Supplementary Figure 2b), indicating that the DBH-weighted metric provides a better fit to the theoretical model. The advantage is strongest at small L, likely because stem counts are most affected by discreteness (a patch containing few trees carries limited information about space occupancy, whereas a patch containing many individuals samples the tree-size distribution more adequately).

In the Michigan dataset, the DBH-weighted CvM proxy is already near or below 1/6 at $L = 10\,\text{m}$, while the stem-count CvM remains elevated. The log-ratio is also largest at small L (median ≈ 1.3 at $L = 10\,\text{m}$), possibly reflecting the larger body size of temperate-forest trees: at fine spatial scales, DBH-weighted abundances seem to provide smoother and more informative measures than stem counts. At larger L, the log-ratio approaches zero as the two metrics become effectively equivalent. This convergence is expected, as discreteness effects diminish when patches aggregate many individuals.

Taken together, these results show that DBH weighting extends the range of spatial scales over which the model provides an accurate description of the data. We therefore adopt the DBH-weighted metric as the primary abundance measure in the main analysis, and $L = 50\,\text{m}$ as the reference spatial scale. The stem-count metric nevertheless provides a reasonable approximation at larger scales, and we use it for comparison with the ecological null model, as it requires fewer assumptions in contstructuing the null models (see section 6).

1.2 Theoretical fits of analyzed species

As explained in the Methods, species were selected by retaining those whose cumulative stem-count abundance reaches 95% of the total, then dropping any with a zero-patch fraction exceeding 90% for adequate spatial sampling. This yields 101 analyzed species for BCI, 396 for Pasoh, and 12 for Michigan at the preferred spatial scale $L = 50$ m. The Pasoh count reflects the high richness of this Malaysian tropical forest: considering all 528 species in Pasoh is difficult because many are too rare to contribute meaningfully to the cumulative abundance threshold, while only 3 additional species are excluded by the zero-fraction criterion. All 396, 101,

and 12 selected species are included in every analysis (vulnerability ranking, regression, neutral comparison, etc.). For visual display only, Supplementary Figures 3 and 5 show the 100 most abundant analyzed species from BCI and Pasoh (ordered by descending total stem-count abundance), and all 12 from Michigan. In each panel, the histogram of observed VAA values is shown alongside the best-fit theory distribution. The species-specific parameters δ_α and $\bar{\beta}_\alpha$ were determined through numerical optimization as described in the Methods. The figures confirm the findings presented in the main text: species-specific VAA distributions are captured well by the theory across a wide range of abundance levels and distributional shapes, with rarer species (later panels) showing sparser histograms as expected.

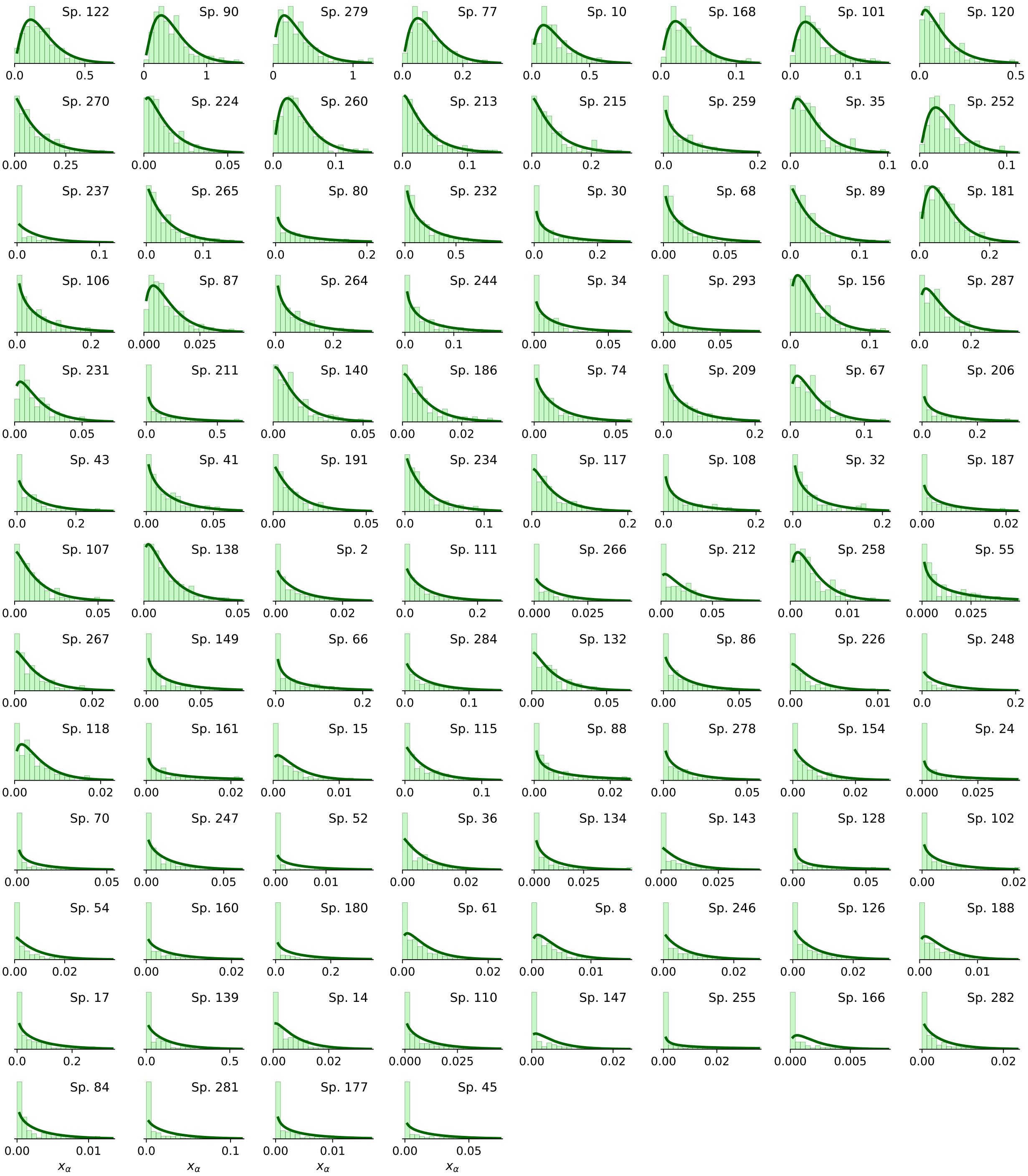

Supplementary Figure 3: **DBH-weighted VAAD fits for the 100 most abundant analyzed species from the BCI dataset ($L = 50$ m; 101 species analyzed in total).** Each panel shows the empirical histogram of x_α (all patches) alongside the theoretical distribution. Species are ordered by descending total stem-count abundance. The species index reported in each panel label is the position of that species in the complete species list as stored in the dataset file: alphabetical order for BCI and Michigan; order of first occurrence among alive trees in the raw census file for Pasoh.

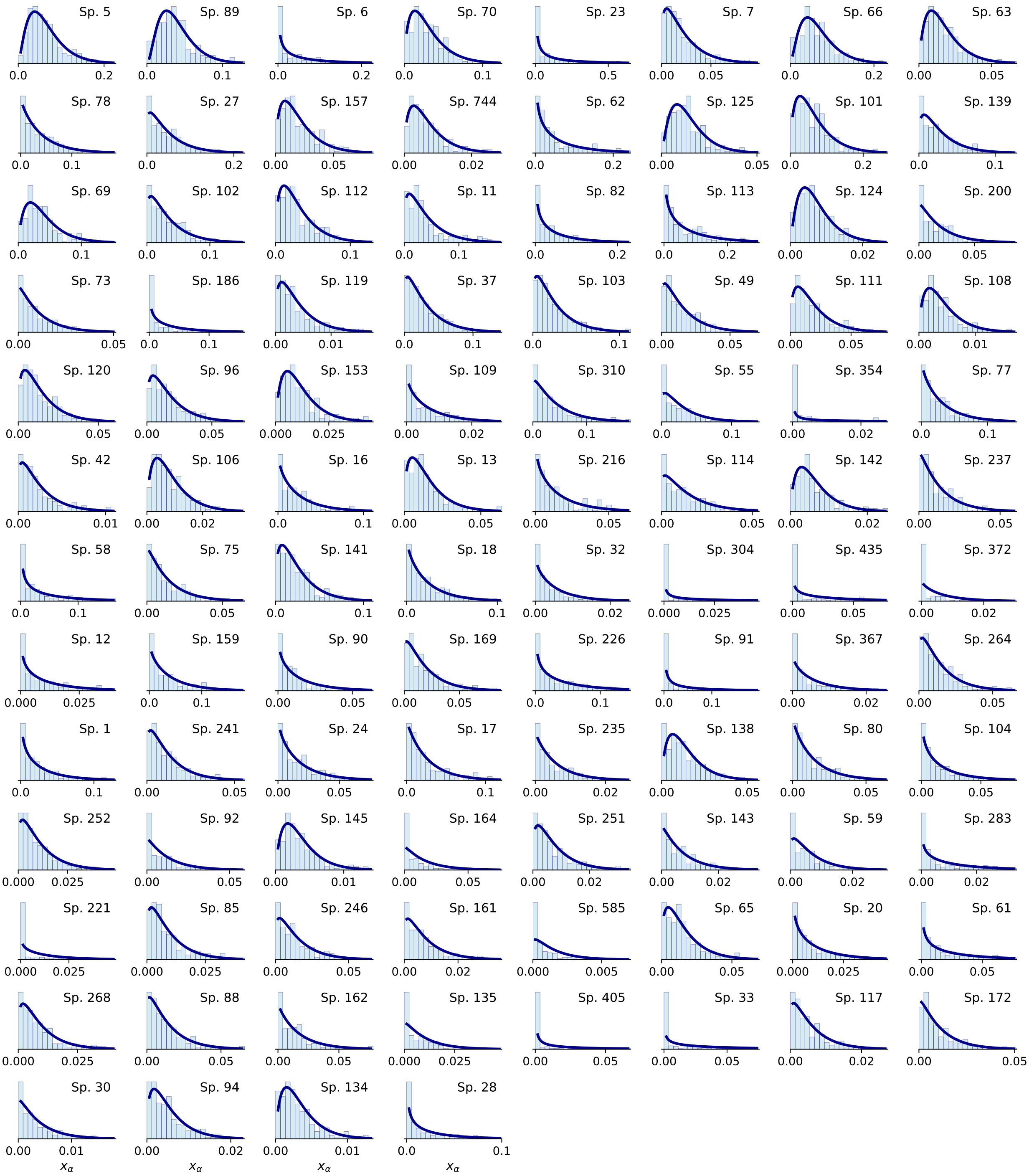

Supplementary Figure 4: **DBH-weighted VAAD fits for the 100 most abundant analyzed species from the Pasoh dataset ($L = 50\,$m; 396 species selected in total).** Same layout as Supplementary Figure 3, with species indexed by position of that species in the complete species list as stored in the dataset file: alphabetical order for BCI and Michigan; order of first occurrence among alive trees in the raw census file for Pasoh.

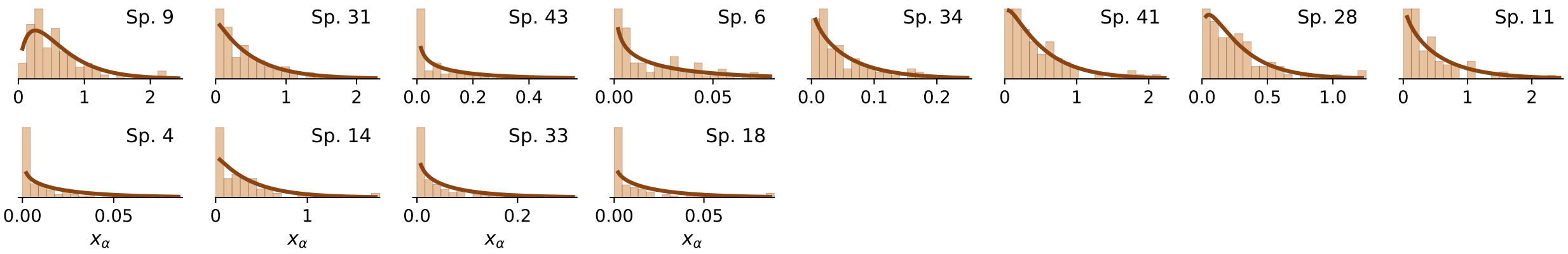

Supplementary Figure 5: **DBH-weighted VAAD fits for all 12 analyzed species from the Michigan dataset ($L = 50\,\mathbf{m}$).** Same layout as Supplementary Figure 3, with species indexed by position of that species in the complete species list as stored in the dataset file: alphabetical order for BCI and Michigan; order of first occurrence among alive trees in the raw census file for Pasoh.

2 Extended spatial cross-validation: out-of-sample predictions

To assess whether the fitted model parameters generalise beyond the patches used for fitting, we performed further spatial cross-validation tests. We considered 20 random 50/50 splits of the N patches and we fitted the model ($\bar{\beta}_\alpha$, δ_α, M) on the training half alone and evaluated predictions on the held-out half. This directly tests independence between fitting and evaluation data. Supplementary Figure 6 summarises all cross-validation results.

Panels **(a)** and **(b)** show the species-specific VAAD parameters $\bar{\beta}_\alpha$ and δ_α estimated from the training half vs. those from the full-data estimates. Points cluster along the diagonal (Pearson correlation $r > 0.97$ in log space for all three datasets).

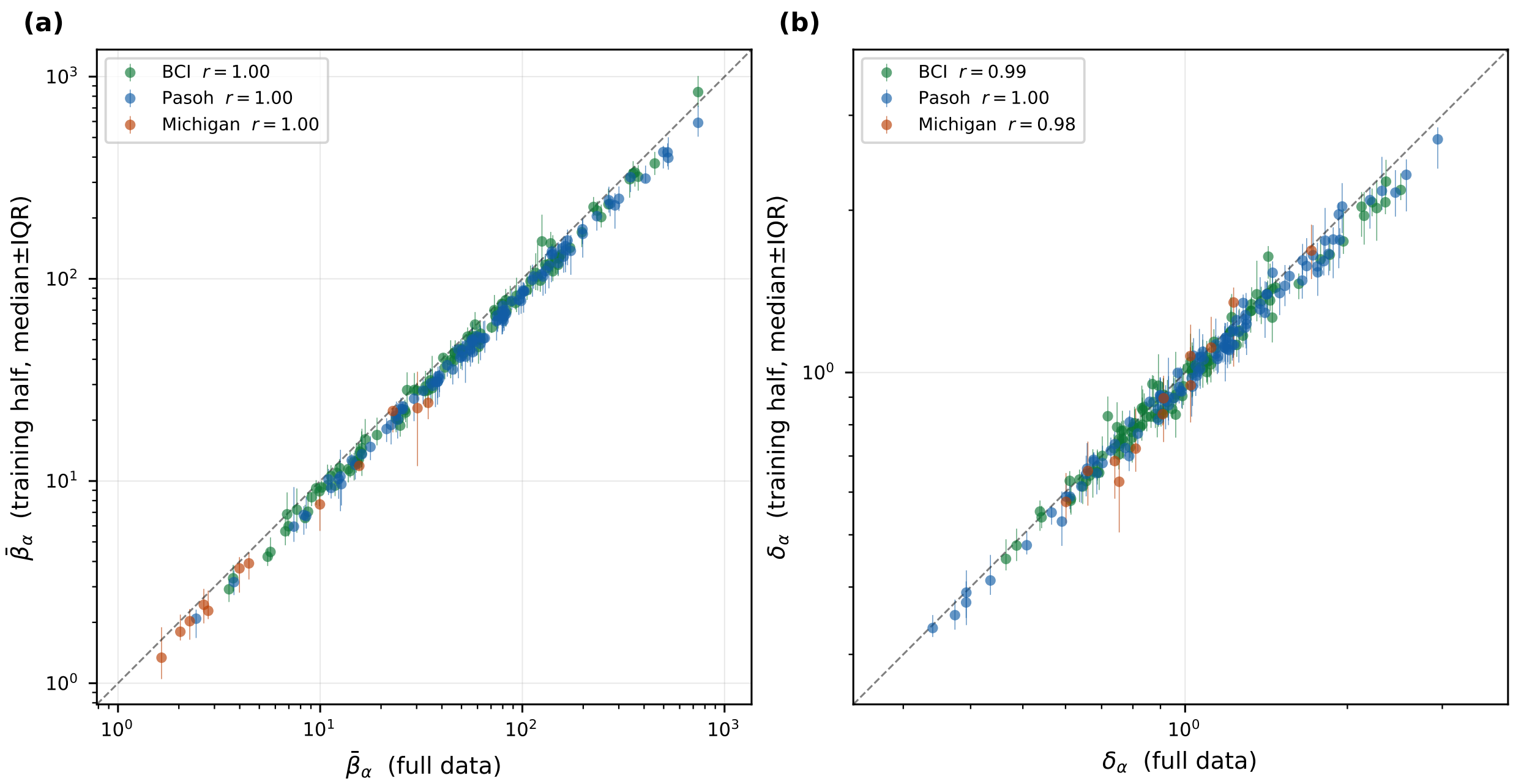

Supplementary Figure 6: **Cross-validation summary.** **(a)** Rate parameter $\bar{\beta}_\alpha$ and **(b)** shape parameter δ_α estimated from a randomly chosen half of the patches (y-axis, median $\pm$ IQR over 20 splits) versus the full-data estimate (x-axis); log–log scale; dashed line: identity; $L = 50\,\text{m}$.

3 Connection between vacancy-adjusted abundance parameters and life history and colonization strategy

To explore whether the VAAD shape parameters carried an ecological interpretation in terms of life-history strategy, we compared VAAD parameter values across functional guilds in the BCI dataset. Guild assignments (shade-tolerant, gap-exploiting, intermediate) were taken from [5]. From the 101 species considered, the split between these groups was 62, 12, and 10, respectively.

The theoretical prediction for the VAAD for species α is a Gamma distribution with shape δ_α and rate $\bar{\beta}_\alpha$. The shape parameter δ_α controls the coefficient of variation of the distribution: $\mathrm{CV}_\alpha = 1/\sqrt{\delta_\alpha}$. Its inverse, δ_α^{-1}, therefore increases monotonically with spatial heterogeneity, and we termed this effective dispersal heterogeneity. Larger δ_α^{-1} (smaller δ_α) corresponds to more right-skewed patch-level abundance patterns, i.e. species that reach high relative abundances in a few patches while being absent from most others, a profile consistent with opportunistic, gap-exploiting colonization. Smaller δ_α^{-1} (larger δ_α) corresponds to a more even spread of the species across patches, consistent with shade-tolerant persistence. The rate parameter $\bar{\beta}_\alpha^{-1}$ is the inverse rate of the Gamma that does not have a direct connection to the spatial concentration structure, but rather to the effective dispersal strength, which is being equal to the mean VAA times its coefficient of variation, thus mixing these aspects. Supplementary Figure 7(a),(b) show the across guilds distribution of $\bar{\beta}_\alpha^{-1}$ and δ_α^{-1}, respectively. Supplementary Figure 7(c),(d) show the aggregated comparison as in the main text (gap-exploiters+intermediate vs. shade tolerant).

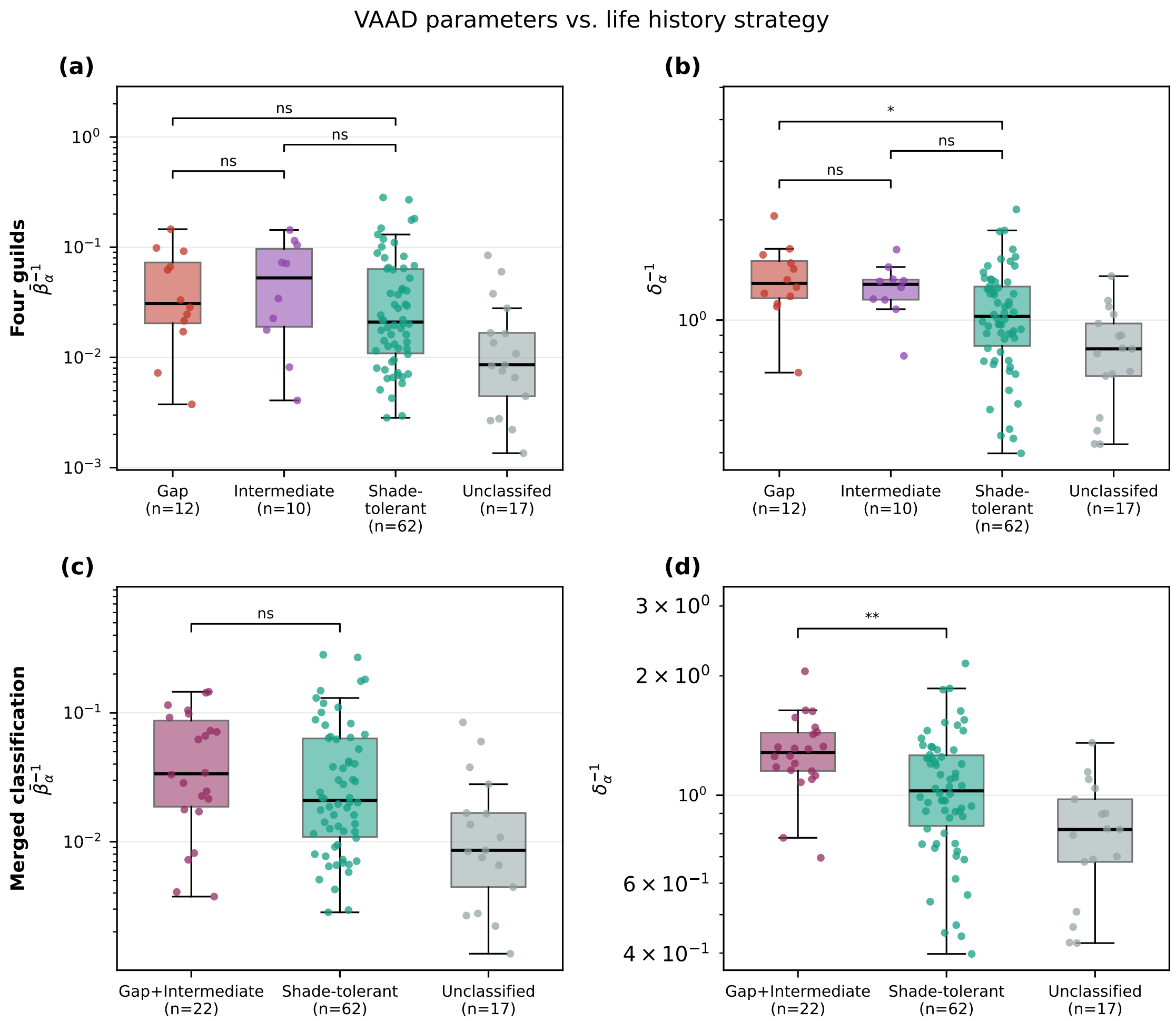

Supplementary Figure 7: **Spatial heterogeneity parameter δ_α^{-1} is higher for gap and intermediate species than for shade-tolerant species.** Panels (a)–(b): four-guild classification (Gap / Intermediate / Shade-tolerant / Unclassified); panels (c)–(d): merged classification (Gap+Intermediate / Shade-tolerant / Unclassified). Left column (a, c): inverse rate parameter $\bar{\beta}_\alpha^{-1}$ (scale of the fitted Gamma, reflecting abundance level); right column (b, d): inverse shape parameter δ_α^{-1} (monotonically related to the coefficient of variation of patch-level relative abundances; higher values indicate greater spatial concentration). Axes are on a log scale. Species are from the BCI dataset ($n_G = 12$, $n_I = 10$, $n_S = 62$, $n_U = 17$; $L = 50\,\mathrm{m}$ patches); guild assignments from [5]. Boxes show median and interquartile range (IQR), with whiskers extending to 1.5×IQR; overlaid points are individual species (jittered horizontally); outliers not shown. Significance brackets show pairwise Mann-Whitney U tests (two-sided) with Holm step-down correction for multiple comparisons; tested groups: G, I, S in panels (a)–(b) and GI, S in panels (c)–(d); Unclassified species are displayed but excluded from testing. Significance thresholds: ns $p > 0.05$, * $p \leq 0.05$, ** $p \leq 0.01$. Exact two-sided Holm-corrected p-values for δ_α^{-1} (panels b,d): G vs. S $p = 0.044$, I vs. S $p = 0.10$, merged GI vs. S $p = 0.004$; all guild comparisons for $\bar{\beta}_\alpha^{-1}$ (panels a,c) give $p > 0.05$. Full p-value table below.

Consistent with this expectation, gap-exploiting and intermediate species tend to have higher δ_α^{-1} than shade-tolerant species: medians are $\delta_G^{-1} \approx 1.29$ (IQR $[1.16, 1.50]$, $n = 12$), $\delta_I^{-1} \approx 1.28$ (IQR $[1.15, 1.32]$, $n = 10$), and $\delta_S^{-1} \approx 1.03$ (IQR $[0.84, 1.26]$, $n = 62$). Pairwise Mann–Whitney U tests with Holm step-down correction yield $p = 0.044$ for G vs. S and $p = 0.10$ for I vs. S, while the merged Gap+Intermediate vs. Shade-tolerant comparison reaches $p = 0.004$. The 17 unclassified species show even lower values ($\delta_U^{-1} \approx 0.82$, IQR $[0.68, 0.98]$), but this group is heterogeneous by construction and is excluded from the formal pairwise tests. Overall, species associated with gap dynamics exhibit higher δ_α^{-1}, consistent with more spatially concentrated abundances, whereas shade-tolerant species show lower δ_α^{-1}, consistent with more uniform spatial distributions.

The rate parameter $\bar{\beta}_\alpha^{-1}$ shows no significant guild-related trend (all pairwise comparisons $p > 0.05$, Holm-corrected). This is consistent with the fact that mean patch-level relative abundance reflects demographic balance (birth, death, competition) rather than dispersal mode: shade-tolerant and gap species can have comparable mean relative abundances while differing substantially in how that abundance is distributed across space. Exact p-values for all pairwise comparisons (both parameters) are reported in the table below.

Mann–Whitney U, Holm-corrected, $\bar{\beta}_\alpha^{-1}$			
Comparison	n_1, n_2	p_{raw}	p_{Holm}
G vs. I	12, 10	0.621	0.773
G vs. S	12, 62	0.359	0.773
I vs. S	10, 62	0.258	0.773

Mann–Whitney U, Holm-corrected, δ_α^{-1}			
Comparison	n_1, n_2	p_{raw}	p_{Holm}
G vs. I	12, 10	0.531	0.531
G vs. S	12, 62	0.015	0.044
I vs. S	10, 62	0.052	0.103

These results are therefore consistent with the interpretation of δ_α^{-1} as encoding aspects of colonization strategy. We note that this conclusion is limited to the BCI dataset, for which the extensive guild classification is available, based on prior field observations [5]. This tripartite scheme is likely an approximation to a continuum of shade tolerance and gap-exploitation tendencies, which would attenuate the statistical signal.

We now show that the VAAD parameters, shown as violin plots in fig. 3D-F of the main text, have a small correlation with the species abundance. To this end, we plot them in a scatter plot Supplementary Figure 8, to investigate visually how these parameters covary with species-level abundance. We see that both effective dispersal strength and heterogeneity are widely spread, and have low correlation with the species abundance, especially at lower abundances. The reduction in parameter spread at higher abundances observed in the BCI and, less apparently, also in the Pasoh datasets arises from the coupled constraints imposed by the model structure. The effective dispersal strength depends on both abundance and the inverse rate parameter, which can compensate at low abundance. At higher values, the inverse rate parameter saturates, causing the increasing trend in $\bar{\beta}_\alpha^{-1}$, the effective dispersal strength. Simultaneously, the local space constraint implies that achieving very high abundance requires increasingly homogeneous spatial distributions, constraining the heterogeneity parameter δ_α^{-1} to lower values. The combination of these effects leads to the observed reduction in spread.

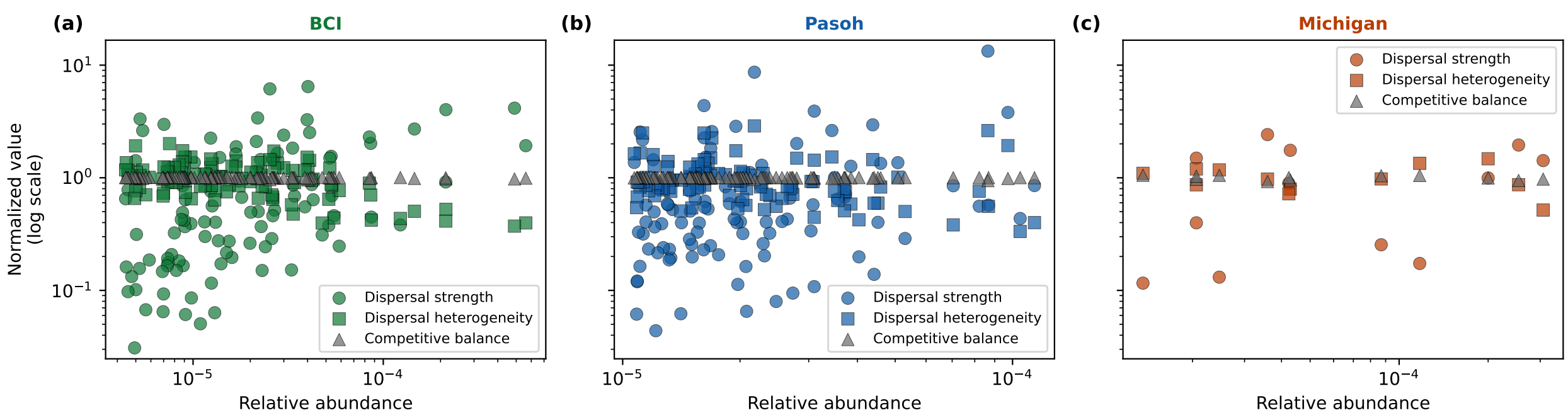

Supplementary Figure 8: **The VAAD parameters do not show strong correlations with species relative abundance.** Scatter plots show the normalised effective dispersal strength ($\bar{\beta}_\alpha^{-1}$, circles), dispersal heterogeneity (δ_α^{-1}, squares), and competitive balance (B_α, triangles) plotted against the mean relative abundance computed from the stem-count metric. are shown for all three datasets (BCI, Pasoh, Michigan) at the canonical spatial scale $L = 50\,\mathrm{m}$. Panels **(a)**–**(c)** correspond to the three datasets in the same order as the main-text figures, showing the 100 most abundant analyzed species per dataset ($n = 100$ for BCI and Pasoh, $n = 12$ for Michigan). All three quantities span roughly an order of magnitude around their mean, and are widely scattered, especially at lower abundances. The parameters exhibit mild trends with increasing abundance: dispersal strength (circles) tends to increase with rarity, while heterogeneity (squares) shows more scatter and a mildly decreasing trend. See text for interpretation.

4 Robustness of the homogeneous capacity assumption

In fitting the model to data, we assumed a constant patch capacity M shared across all patches. To assess whether allowing patch-to-patch heterogeneity in M improves fit quality, we implemented an alternative procedure in which each patch j is assigned its own capacity M_j. This augmented fitting proceeded in two phases. Phase 1 identified the optimal scalar (patch-independent) M_0 as described in the Methods. Phase 2 then optimised each M_j independently via 1D scalar minimisation of the cross-species CDF-MSE computed within each patch, with a regularisation term $\lambda_{\text{homog}} \log(M_j/M_0)^2$ penalising deviations from M_0. Optimisation was terminated when either (i) the relative improvement in total GOF fell below 10^{-4} for 10 consecutive iterations, or (ii) the mean per-patch drift $\langle|M_j^{(t)} - M_j^{(t-1)}|\rangle/M_0$ dropped below 10^{-3} (activated only after the first 10 iterations).

Supplementary Figure 9 summarises the comparison at $L = 50$ m for the DBH-weighted (crown) metric across all three datasets. Panel (a) shows the distribution of fitted M_j/M_0. For BCI ($S = 101$) and Pasoh ($S = 396$), values remain tightly clustered around M_0 (IQR $[0.90, 1.02]$ and $[0.96, 1.07]$, respectively), and both fits converge within ≤ 12 outer iterations. In contrast, for Michigan ($S = 12$), the optimiser drives M_j systematically below M_0 (median 0.72, IQR $[0.66, 0.80]$), with convergence only after ~ 30 iterations.

Panel (b) reports per-species CDF-MSE (baseline on the x-axis, heterogeneous-M fit on the y-axis). For BCI and Pasoh, points cluster near the diagonal: roughly half the species improve (58/101 for BCI, 204/396 for Pasoh), and the median CDF-MSE change is below 2% in either direction, which seems negligible relative to the $\sim$ 200 to 300 additional free parameters. For Michigan, most species lie above the diagonal (9/12 worsen, median CDF-MSE +106%). This degradation is a likely consequence of limited species richness: the cross-species CDF within each patch provides too little information to constrain M_j, leading to a nearly flat objective landscape. The optimiser can therefore drift away from M_0 without genuine improvement, settling in suboptimal local minima.

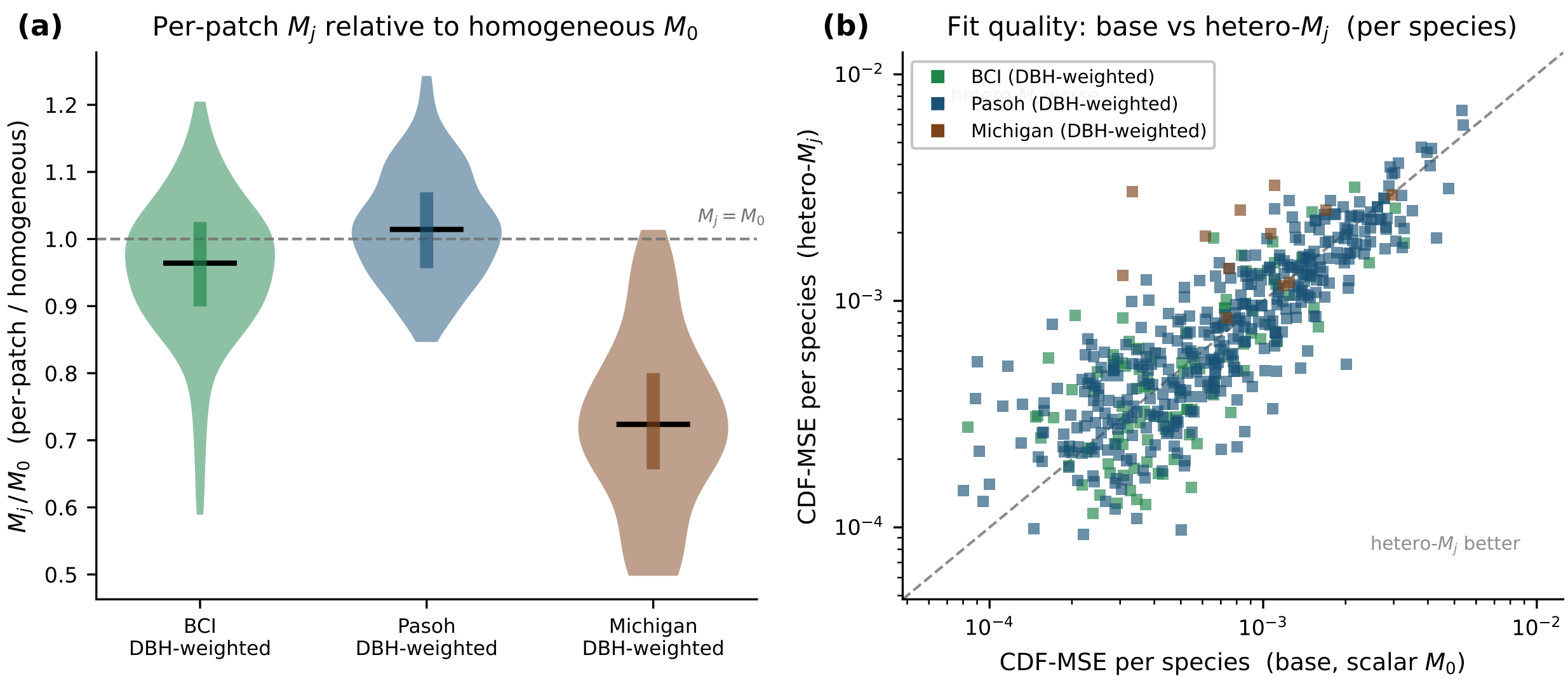

Supplementary Figure 9: **Comparison of homogeneous-M (base) and per-patch-M (heterogeneous carrying capacity M_j) fits at $L = 50$ m, DBH-weighted metric.** **(a)**: violin plots of M_j/M_0 for each dataset (S = number of selected species). The violin outline is a kernel-density estimate of the per-patch M_j/M_0 distribution; the thick vertical bar shows the IQR and the black horizontal line marks the median; dashed line marks $M_j = M_0$; regularisation strength $\lambda_{\text{homog}} = 1$. BCI and Pasoh ($S = 101$, 396) stay close to M_0; Michigan ($S = 12$) drifts to $\approx 0.72\, M_0$ due to the flat per-patch loss landscape. **(b)**: per-species CDF-MSE scatter (base on x, hetero-M_j on y); points below (above) the diagonal indicate better (worse) fit with per-patch M. Michigan points cluster above the diagonal, confirming that per-patch M is ill-constrained when S is small.

Overall, this analysis supports the use of a homogeneous scalar M on both parsimony and robustness grounds: for species-rich datasets (BCI, Pasoh), there is no detectable benefit from introducing patch-level heterogeneity, while for species-poor datasets (Michigan), the heterogeneous model becomes overparameterized and weakly constrained by the available data. It is likely that the natural heterogeneity is already effectively accounted for by the model through the interaction-dispersal kernel heterogeneity.

5 Test for effective inter-species facilitation

Introducing an *explicit* interspecific facilitation term into our model is pracically challenging, as it would require either prior knowledge of *which* species interact positively and with what strength. Alternatively, it would require introducing a large number of additional parameters (of order $S \times S$, i.e. thousands of free parameters).

To test in a parsimonious but principled way whether additional interspecific facilitation is detectable in the data beyond what already accounted for in the DMFT model, we introduce for each species α a correction parameter Ω_α that modifies its effective occupancy and is optimised to minimise the CDF-MSE of the fitted Gamma distribution. A non-negligible Ω_α would indicate that species α effectively occupies a larger share of free space than predicted by the baseline model, consistent with a facilitative effect at the metacommunity level.

In practice, the additional parameter vector $\Omega = (\Omega_\alpha) \in [0,1]^S$ rescales each species' effective patch occupancy as

$$\tilde{p}_{\alpha i} = (1 - \Omega_\alpha)\, p_{\alpha i},$$

and the modified vacancy-adjusted abundance is defined using the same expression as in the main text:

$$\tilde{x}_{\alpha i} = \frac{\tilde{p}_{\alpha i}}{1 - \sum_\beta \tilde{p}_{\beta i}}.$$

Because all species share the free-space denominator, a non-zero Ω_α perturbs $\tilde{x}$ for all species simultaneously. The constraint $\Omega_\alpha \in [0,1]$ ensures that the correction can only reduce species α's effective competitive footprint, consistent with a facilitation scenario in which this species effectively increases available space.

To estimate Ω^*_α, we perform alternating optimisation starting from $\Omega = 0$ and the baseline-fit parameters $(\bar{\beta}_\alpha, \delta_\alpha)$, with M fixed at M_0. An Ω-*step* minimises $\sum_\alpha \text{CDF-MSE}(\tilde{x}_\alpha;\, \text{Gamma}(\delta_\alpha, 1/\bar{\beta}_\alpha)) + \lambda \sum_\alpha \Omega_\alpha$ over $\Omega \in [0,1]^S$ using L-BFGS-B with $(\bar{\beta}, \delta)$ fixed. This is followed by a *parameter-update step*, in which $(\bar{\beta}_\alpha, \delta_\alpha)$ are refitted for each species using $\tilde{x}_\alpha$ with Ω fixed. The regularisation coefficient λ penalises nonzero Ω_α; we consider $\lambda \in \{0,\, 0.001,\, 0.01,\, 0.1\}$ to distinguish robust signals from optimisation artefacts arising from a shallow cost landscape.

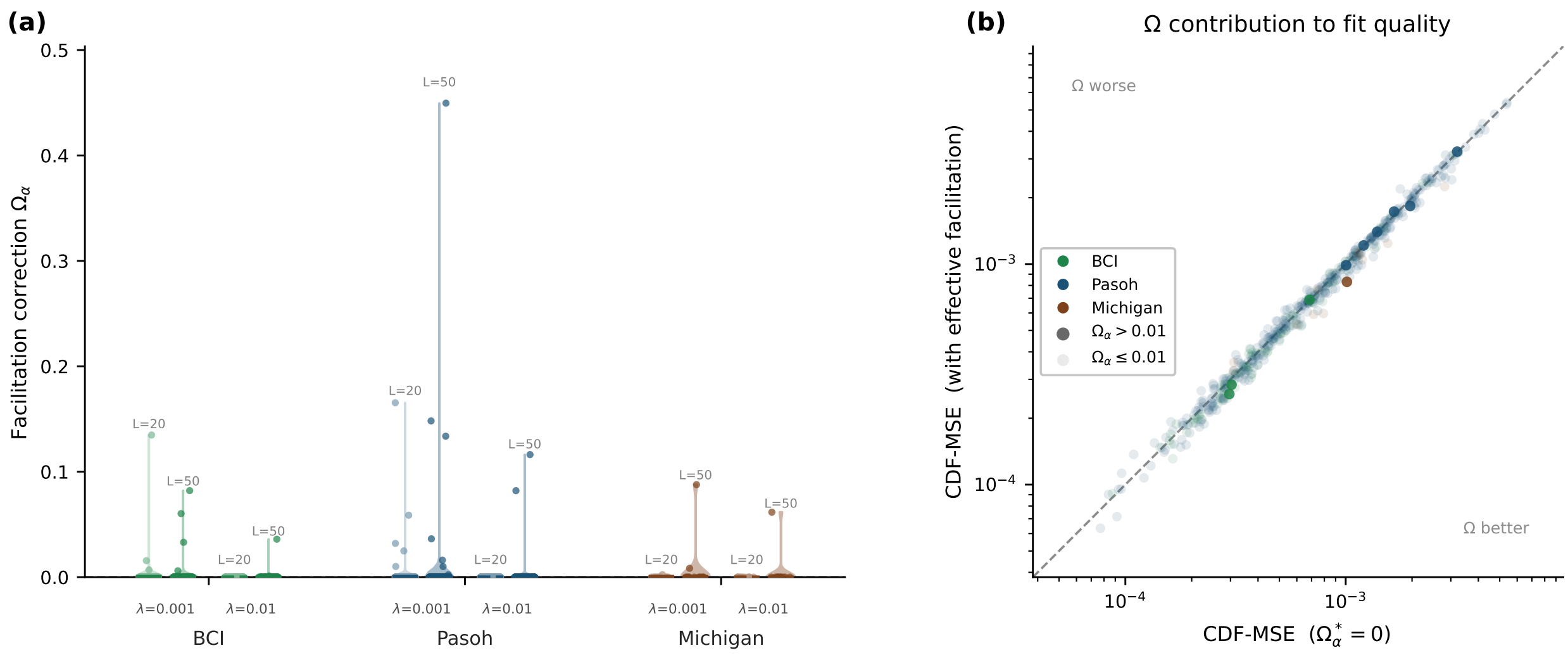

Supplementary Figure 10: **Test for effective inter-species facilitation. (a)**: facilitation correction Ω_α per species shown as violin plots, whose outline is a kernel-density estimate of the per-species Ω_α distribution, with individual-species dots overlaid (horizontal bar = median), for three datasets (all selected species: $n = 101$ for BCI, 396 for Pasoh, 12 for Michigan) at two patch scales ($L = 20\,\text{m}$ lighter shade, $L = 50\,\text{m}$ darker shade) and two regularisation strengths ($\lambda = 0.001$ left, $\lambda = 0.01$ right within each dataset group). All medians are zero across all datasets, scales, and regularisation strengths; one Pasoh species reaches $\Omega_\alpha \approx 0.45$ at $L = 50\,\text{m}$ under $\lambda = 0.001$, but collapses at $\lambda = 0.01$. **(b)**: per-species CDF-MSE scatter at $L = 50\,\text{m}$ isolating the Ω_α contribution: x-axis = CDF-MSE after refitting $(\bar{\beta}_\alpha, \delta_\alpha)$ with $\Omega^*_\alpha = 0$ (*refitted baseline*); y-axis = CDF-MSE with Ω^*_α free ($\lambda = 0.001$); full-colour points have $\Omega_\alpha > 0.01$. Points cluster on the diagonal, confirming no systematic improvement from allowing $\Omega_\alpha \neq 0$.

Supplementary Figure 10 shows the results for all three datasets at two patch scales ($L = 20\,\text{m}$ and $L = 50\,\text{m}$) and two regularisation strengths ($\lambda \in \{0.001,\, 0.01\}$; panel a). In all cases, the distribution of

Ω_α is tightly concentrated near zero. A small number of species exhibit non-negligible values at the weakest regularisation: for example, one species in Pasoh reaches $\Omega_\alpha \approx 0.45$ at $L = 50\,\mathrm{m}$ ($\lambda = 0.001$), but this effect is strongly reduced at $\lambda = 0.01$.

Panel (b) isolates the contribution of Ω_α to fit quality at $L = 50\,\mathrm{m}$. The x-axis shows the CDF-MSE after refitting $(\bar{\beta}_\alpha, \delta_\alpha)$ with $\Omega_\alpha = 0$ (refitted baseline), while the y-axis shows the CDF-MSE with Ω^*_α free at $\lambda = 0.001$. Points cluster tightly along the diagonal: the median improvement in GOF relative to the refitted baseline is +3 % (BCI), +1 % (Pasoh), and +12 % (Michigan, driven by a single species out of 12).

These results suggest a nuanced picture. The marginal improvements and the small number of non-zero Ω_α values, more apparent in species-rich tropical forests, indicate that effective facilitation may leave a detectable signature in the spatial abundance structure of some species. However, the present species-level scalar correction introduces S additional free parameters without yielding a substantial improvement in fit quality, and the signal weakens with increasing regularisation strength. We therefore retain the standard model ($\Omega_\alpha = 0$) in the present work. We conclude that the effective facilitation is possibly already captured as effective buffering of the space limitation in our model. At the same time, incorporating facilitation in a more principled form, for example through pairwise interaction terms constrained by trait or phylogenetic similarity—remains a natural and important direction for future extensions of the framework.

6 Comparison with ecological null models

We compare our framework against three Negative Binomial (NegBin) null models of increasing parameter parsimony. For species α in patch j, we assume that the number of individuals $n_{\alpha j} \sim \mathrm{NegBin}(r_\alpha,, \mu_\alpha)$ independently across species and patches. This defines a phenomenological null model in which abundances are overdispersed but lack interspecific interactions and spatial coupling. The NegBin distribution is widely used to model overdispersed ecological count data [6, 7, 8] and arises naturally from Poisson–Gamma mixtures or simple birth–death processes [9]. Similar overdispersed abundance distributions also emerge in neutral models of biodiversity [10, 11].

The three variants we consider differ in parameter sharing: a fully neutral model (shared r and μ), a species-specific model (independent r_α, μ_α), and an intermediate model with shared dispersion and species-specific means. Parameter counts are summarized in Supplementary Table 2. The probability mass function is

$$P(n_{\alpha j} = n) = \binom{n + r_\alpha - 1}{n} \left(\frac{r_\alpha}{r_\alpha + \mu_\alpha}\right)^{r_\alpha} \left(\frac{\mu_\alpha}{r_\alpha + \mu_\alpha}\right)^{n}, \tag{S3}$$

with mean μ_α and variance $\mu_\alpha + \mu_\alpha^2/r_\alpha$. The dispersion parameter r_α controls aggregation, with $r_\alpha \to \infty$ recovering the Poisson limit. Crucially, all variants assume species independence and lack the shared-denominator coupling present in our framework.

- **Per-species NegBin**. Parameters (r_α, μ_α) are estimated independently per species by maximum likelihood (MLE) on the patch count data $\{n_{\alpha j}\}_{j=1}^N$. The optimiser is seeded with method-of-moments estimates $\hat{\mu}_\alpha = \bar{n}_\alpha$ and $\hat{r}_\alpha = \hat{\mu}_\alpha^2/(\mathrm{Var}(n_\alpha) - \hat{\mu}_\alpha)$; species for which $\mathrm{Var}(n_\alpha) \le \hat{\mu}_\alpha$ (underdispersed) are treated as Poisson ($r_\alpha \to \infty$). Together with M_p (see below), this variant has $2S + 1$ parameters, matching our framework.
- **Shared-r NegBin.** All species share a single dispersion parameter r; per-species means are fixed at $\mu_\alpha = \bar{n}_\alpha$, so only r is estimated numerically per species by maximising the total log-likelihood $\sum_\alpha \log P(\{n_{\alpha j}\} \mid r, \bar{n}_\alpha)$. In this version, species can differ in mean abundance but must share the same aggregation structure. Total parameters: $S + 2$.
- **Fully neutral NegBin.** Both dispersion and mean are shared: $\mu = \bar{n}$ is the grand mean of all counts $\{n_{\alpha j}\}$ over all species and patches, and r is estimated by maximising $\sum_\alpha \log P(\{n_{\alpha j}\} \mid r, \bar{n})$. This is the most stringent null, as all individuals are statistically exchangeable across species and patches, requiring only 3 parameters in total.

Supplementary Table 2: **Parameter count per model.** S = number of selected species. All pipelines include a capacity parameter, M_0 (our model) or M_p (NegBin), independently estimated from the data.

Model	Parameters	Count
Interacting DMFT model (this work)	$(\bar{\beta}_\alpha, \delta_\alpha)$ per species + global M_0	$2S + 1$
NegBin per-species (non-neutral)	(r_α, μ_α) per species + capacity M_p	$2S + 1$
NegBin shared-r (intermediate)	shared r + $\mu_\alpha = \bar{n}_\alpha$ per species + M_p	$S + 2$
NegBin (fully neutral)	shared r + shared $\mu = \bar{n}$ (grand mean) + M_p	3

- **Relative goodness-of-fit comparison in x_s space (KS statistic).** To compare both model families on equal footing, we evaluate them in the common rescaled-abundance space x_s and apply an identical sample-generation procedure to each. For every model, Z independent synthetic datasets of size (S, N) are generated from the fitted parameters, and the Kolmogorov–Smirnov (KS) statistic between the observed and synthetic x_s distributions is computed (restricted to positive x_s). For our DMFT model, samples are drawn directly in x_s space from Gamma$(\delta_\alpha, 1/\bar{\beta}_\alpha)$; NegBin samples are drawn in count space and then transformed to x_s using the same rescaling as the emprical observations, eq. (S2). This common evaluation framework ensures that differences in fit reflect model structure rather than the choice of representation. For the NegBin pipeline, we independently estimate its community capacity M_p to avoid circularity with the DMFT parameter M_0, which depends on inter-species coupling. Using the large-count limit $p_{\alpha j} = n_{\alpha j}/M \sim \text{Gamma}(r_\alpha, \mu_\alpha/M)$, we scan $M > \max_j T_j = M - \sum_\beta n_{\beta j}$ and select
$$M_p = \arg\min_{M>\max_j T_j} \sum_\alpha \text{CDF-MSE}_\alpha(M). \tag{S4}$$
Each model is thus evaluated using its own optimally estimated capacity, ensuring a fully self-contained and fair comparison. The resulting estimates are numerically close but not identical (Supplementary Figure 11, inset). The KS statistic is computed from $Z = 500$ synthetic datasets and summarized as medians and interquartile ranges over the selected species.

- **Absolute fit quality assessment (CvM proxy).** To complement the relative comparison, we assess absolute fit quality using a Cramér–von Mises (CvM) proxy computed directly from the empirical data, following the procedure described in section 1. This metric does not rely on synthetic sampling and provides an independent measure of goodness of fit.

Because the NegBin is defined on non-negative integers, all comparisons are performed on stem-count data (see section 1). Extending the analysis to DBH-weighted data would require an additional model for stem sizes and is therefore not considered here.

Supplementary Figure 11 summarises the KS comparison. Panel (a) shows the log-ratio $\log(D_{\text{null}}/D_\Gamma)$ at the canonical patch size $L = 50$ m for all three datasets; positive values indicate that the neutral model fits *worse* than the Interacting DMFT model. At $L = 50$ m, our model generally outperforms the per-species NegBin—the only comparator with the same parameter count ($2S+1$, Supplementary Table 2). The median log-ratio is 0.09 (BCI), 1.65 (Pasoh), and 0.35 (Michigan), with the advantage most pronounced for Pasoh. One-sided Wilcoxon signed-rank tests (H_0: median log-ratio $= 0$, H_1: > 0) show that for the per-species NegBin the advantage is not statistically significant for BCI ($p = 0.54$), modestly significant for Michigan ($p = 0.046$), and highly significant for Pasoh ($p < 0.001$). The shared-r and fully neutral NegBin variants are significantly worse than the Interacting DMFT model across all datasets (see table below), indicating that species-level variation in spatial aggregation is necessary to describe the data.

One-sided Wilcoxon signed-rank test: $\log(D_{\text{null}}/D_\Gamma) > 0$			
	BCI	Pasoh	Michigan
NB (per-sp.) (W)	2496	5050	61
NB (per-sp.) (p)	0.540	<0.001	0.046
NB (shared-r) (W)	3970	5050	75
NB (shared-r) (p)	<0.001	<0.001	0.001
NB (fully neut.) (W)	5050	5050	78
NB (fully neut.) (p)	<0.001	<0.001	<0.001

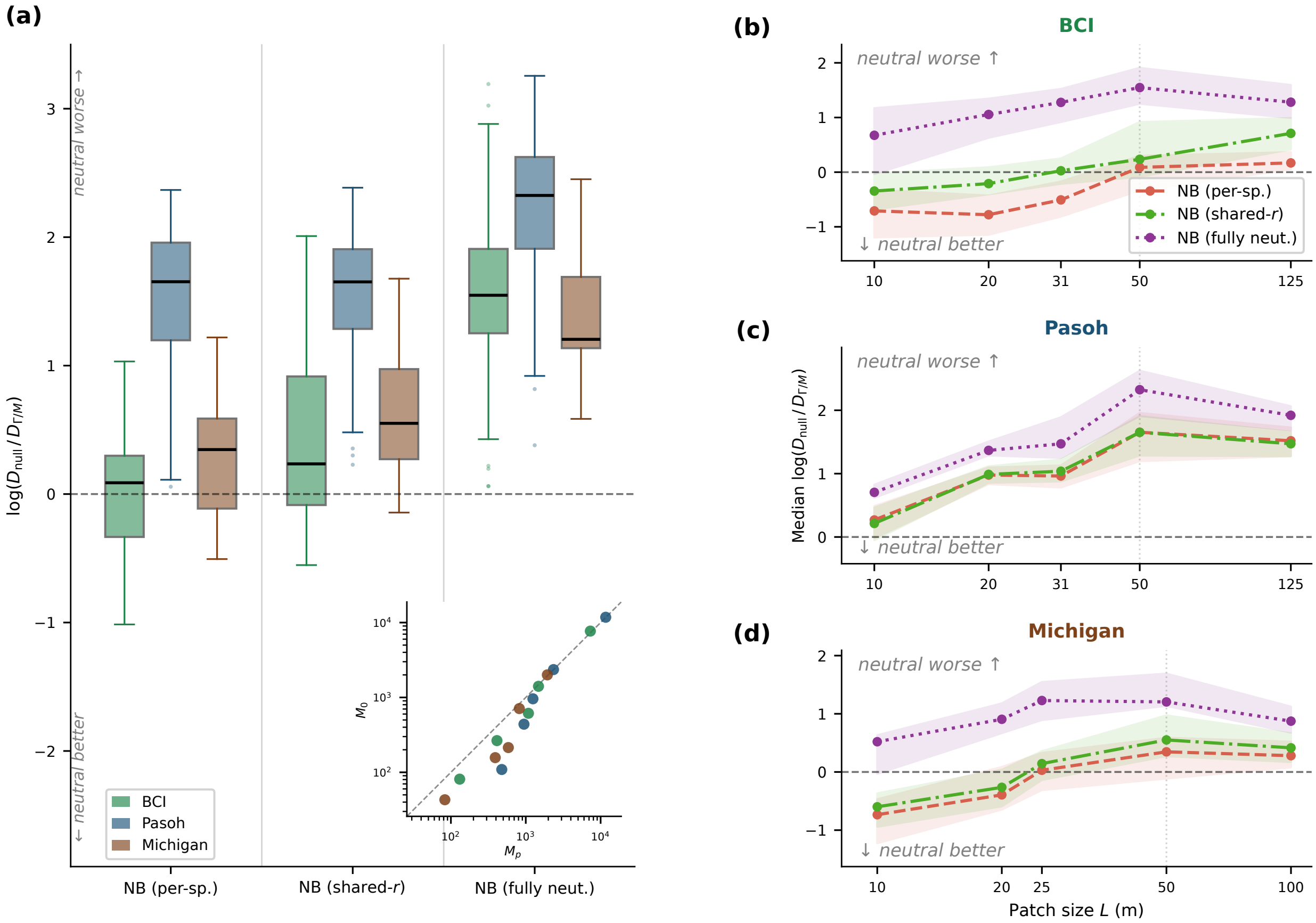

Supplementary Figure 11: **Interacting DMFT model vs. neutral NegBin models: KS statistic comparison.** (a): log-ratio $\log(D_{\text{null}}/D_\Gamma)$ of the KS statistic (positive x_s) at $L = 50$ m, grouped by neutral model variant and coloured by dataset. Boxes: inter-quartile range; bar: median; whiskers: 1.5×IQR; dots: outliers beyond the whiskers. Positive values indicate the neutral model fits *worse* than the Interacting DMFT model; dashed line at zero. One-sided Wilcoxon signed-rank tests (H_0: median log-ratio = 0) against the per-species NegBin give $p = 0.54$ (BCI), $p < 0.001$ (Pasoh), and $p = 0.046$ (Michigan); the shared-r and fully neutral variants are significant across all datasets (full statistics in the table below). Inset: our model's capacity M_0 vs. neutral-pipeline capacity M_p across all (dataset, L) combinations; dashed line: identity; both axes logarithmic. (b)–(d): median log-ratio vs. patch size L for BCI, Pasoh, and Michigan; shaded bands: inter-quartile range across selected species; vertical dotted line: canonical scale $L = 50$ m. Computed from $Z = 500$ synthetic datasets per model; stem-count metric. Error statistics are aggregated over the 100 most abundant species per dataset ($n = 100$ for BCI and Pasoh, $n = 12$ for Michigan); every model is nonetheless fit on the full selected species set. This cap gives each dataset a comparable species count and restricts the comparison to the abundant species, where the null-model fits are well-determined.

The per-species NegBin provides the fairest comparison: both models have $2S + 1$ parameters. However, M_0 in our framework emerges from inter-species coupling, whereas M_p acts as a phenomenological conversion factor without a mechanistic role. That the Interacting DMFT model outperforms the NegBin at ecologically relevant scales on equal parameter footing suggests that inter-species coupling through M carries predictive signal beyond what an independent count model can capture. Moreover, the DMFT shape parameter δ_α has a direct mechanistic interpretation as controlling the degree of spatial aggregation, and we show in section 1 and the guild analysis above that it correlates with ecological strategies (gap-exploitation vs. shade tolerance); a purely phenomenological fit of a NegBin distribution would not provide this inferential link.

Panels (b)–(d) of Supplementary Figure 11 show the full L-dependence across all patch sizes (BCI, Pasoh, and Michigan). At small L (many fine patches, low per-patch counts), the per-species NegBin is competitive with or slightly better than the Interacting DMFT model. This is consistent with section 1.1, as our model does not fit the data well at small spatial scales, whereas a direct count-space fit has an advantage. For $L \geq 31$-50 m, the regime of our main analysis, the Interacting DMFT model matches or outperforms the per-species NegBin. The advantage increases with L and is especially pronounced for Pasoh (log-ratio ≈ 1.5-1.7 at $L = 50$-125 m). The shared-r and fully neutral NegBin variants perform substantially worse at all but the smallest L. The inset comparing M_0 and M_p (lower right of panel (a)) shows that the two estimates

track each other closely (both scale as L^2), confirming that the two pipelines independently recover the same underlying community capacity.

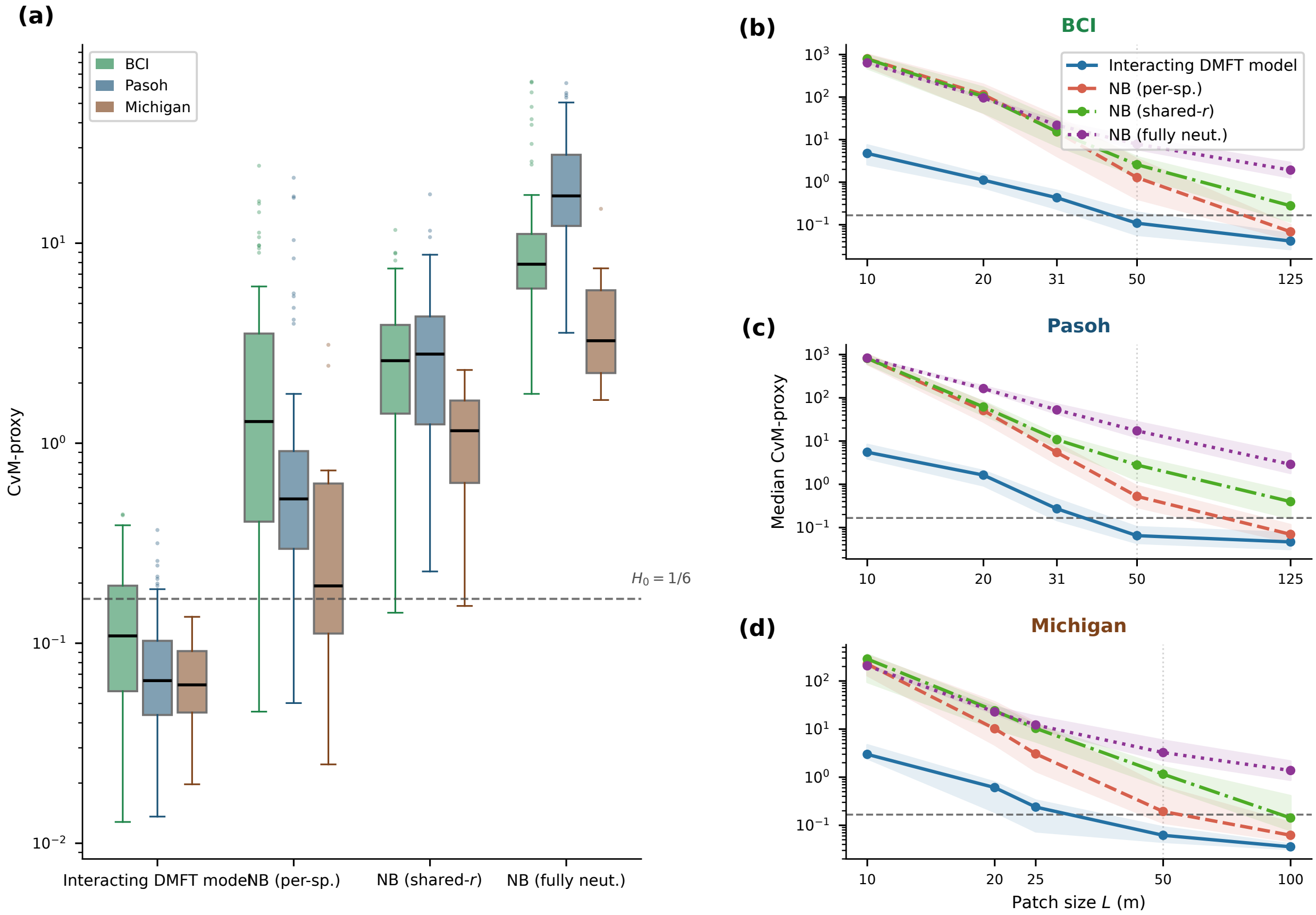

Supplementary Figure 12: **Interacting DMFT model vs. neutral NegBin models: CvM-proxy comparison.** (a): CvM-proxy for all four models at the canonical scale $L = 50$ m, grouped by model and coloured by dataset. Boxes: inter-quartile range; bar: median; whiskers: 1.5×IQR; dots: outliers beyond the whiskers. Dashed line: approximate reference value $\omega^2 = 1/6$ expected under a correctly specified model with converged parameter estimates; values substantially above this line indicate model misspecification. (b)–(d): median CvM-proxy vs. patch size L for BCI, Pasoh, and Michigan; shaded bands: inter-quartile range across selected species; vertical dotted line: canonical scale $L = 50$ m. Interacting DMFT CvM is evaluated in x_s-space (positive observations, continuous Gamma CDF); NegBin CvM is evaluated in count-space (all N patches, discrete NegBin CDF). The two statistics cannot be compared numerically with each other; each is interpreted against the 1/6 reference independently (for discrete distributions this reference is approximate). Same color and line coding as Supplementary Figure 11. As in Supplementary Figure 11, the CvM-proxy is aggregated over the 100 most abundant species per dataset ($n = 100$ for BCI and Pasoh, $n = 12$ for Michigan), while every model is fit on the full selected species set.

Supplementary Figure 12 shows the CvM proxy for all four models. Panel (a) reports values at $L = 50$ m; panels (b)–(d) show the full L-dependence. At $L = 50$ m, the Interacting DMFT model yields median CvM values near or below 1/6 across all datasets (BCI: 0.11, Pasoh: 0.07, Michigan: 0.06), consistent with an adequately specified model (the mild downward deflation relative to 1/6 is expected when parameters are estimated from the same data). All NegBin variants lie substantially above 1/6: the per-species NegBin reaches 0.19 / 0.53 / 1.28, the shared-r variant 1.15 / 2.79 / 2.59, and the fully neutral NegBin 3.25 / 17.23 / 7.85 (Michigan / Pasoh / BCI).

Because these statistics are computed in different spaces (Gamma in x_s; NegBin in count space), their values cannot be compared directly across model families; however, each model can be assessed against its own 1/6 reference. The conclusion is consistent with the KS analysis: the Interacting DMFT model is the only model whose CvM statistic approaches the expected reference value, while all NegBin variants remain substantially above it.

7 Data requirements and parameter convergence

A practical concern when comparing models is data efficiency: how many patches are required for parameter estimates to reliably characterise the model? We address this by subsampling the full set of patches at $L = 50$ m, refitting all four models from scratch on each subsample, and evaluating the CvM proxy on the refitted model. Because the CvM proxy approaches $\sim 1/6$ when a correctly specified model is well estimated (independently of sample size), the resulting learning curves provide a direct diagnostic of parameter convergence: curves that approach 1/6 indicate reliable estimation, whereas curves that plateau above 1/6 for all N' indicate model misspecification rather than insufficient data. Supplementary Figure 13 shows the results. Three features stand out: i) The DMFT model CvM approaches $\approx 1/6$ as N' increases in all three datasets, at approximately $N' \approx$ 100–140 patches for BCI and Pasoh (50–70% of the available data at $L = 50$ m) and $N' \approx$ 30–46 patches for Michigan (30–50%). The mild downward deflation below 1/6 at full N reflects the expected in-sample bias from estimating two parameters per species from the same data. ii) The per-species NegBin converges to approximately 0.19 (Michigan), 0.53 (Pasoh), and 1.28 (BCI) at full N—all well above 1/6—and adding more patches does not reduce this plateau. This shows that the gap between the NegBin and the 1/6 reference is not due to limited data: even with all available patches, the per-species NegBin remains a substantially worse description of the x_s distributions. iii) Shared-r and fully neutral NegBin are flat across N'. Their CvM values are essentially independent of N', confirming that these variants are misspecified in a way that cannot be remedied by collecting more data.

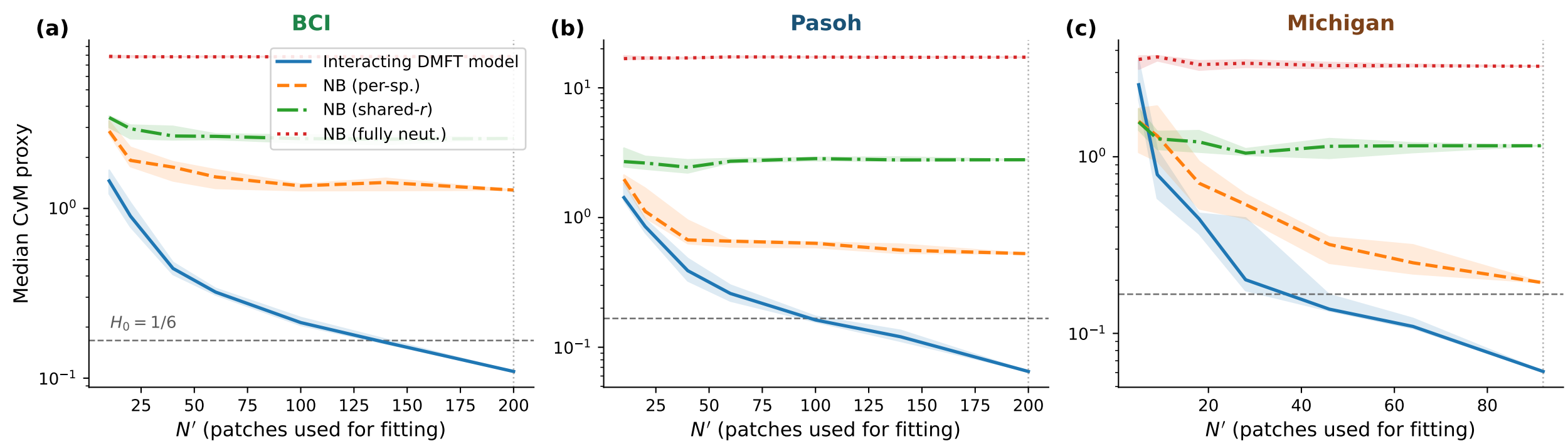

Supplementary Figure 13: **Data overhead: CvM-proxy learning curves at $L = 50$ m.** Each panel shows the median CvM proxy (log scale) vs. the number of patches N' used for fitting, across 10 bootstrap subsamples at $L = 50$ m. Lines: median over selected species; shaded bands: inter-quartile range over bootstrap repetitions. Dashed line: approximate reference value expected under a correctly specified model with converged parameter estimates. Dotted vertical line: full dataset ($N' = N$). The Interacting DMFT model (blue, solid) passes through 1/6 as N' increases, indicating reliable parameter estimation well within the available data range; all NegBin variants plateau above 1/6. As in the main comparison (Supplementary Figure 12), the DMFT and NegBin statistics are evaluated in different spaces (x_s space and count space, respectively) and should therefore be assessed against the 1/6 reference independently, rather than compared numerically across model families.

Practical prescription for new applications

The scale-selection and data-requirements analyses together define a two-step protocol for applying the DMFT model framework to a new forest census. First, determine the optimal patch size L_{opt} by computing the CvM proxy across available spatial scales and identifying the smallest L at which it drops to $\approx 1/6$ (following Supplementary Figure 2). Second, verify that the census contains at least N_{min} patches of size L_{opt}, where N_{min} is the subsample size at which the DMFT learning curve in Supplementary Figure 13 approaches the 1/6 reference.

Based on the present datasets, $N_{\text{min}} \approx$ 100–140 patches for the high-diversity tropical forests (BCI and Pasoh), corresponding to a minimum censused area of $N_{\text{min}} L_{\text{opt}}^2 \approx$ 25–35 ha at $L_{\text{opt}} = 50$ m. For the lower-diversity temperate forest (Michigan), $N_{\text{min}} \approx$ 30–46 patches suffice, corresponding to approximately 7-12 ha. The reduction in N_{min} with decreasing diversity is expected: the model has $2S+1$ free parameters, so species-rich communities require proportionally more patches for reliable parameter estimation.

Finally, these estimates are obtained for complete spatial censuses, in which all individuals within each patch are recorded. For surveys with incomplete detection or systematically missing patches, the effective spatial information is reduced in ways that depend on the spatial structure of the gaps, and a dedicated analysis would be required.

Vulnerability ranking robustness

A central output of the DMFT model is the per-species vulnerability index W_α. We assessed how reliably this ranking can be recovered from subsampled censuses using the same patch-subsampling protocol as in the data-requirements analysis above. For each dataset at $L = 50\,\mathrm{m}$, we drew $n_{\mathrm{rep}} = 10$ random subsamples of N' patches (with N'/N in the range 0.05 to 1.0), refitted the DMFT model from scratch on each subsample, recomputed W_α, and compared the resulting species ranking to the reference ranking obtained from the full dataset.

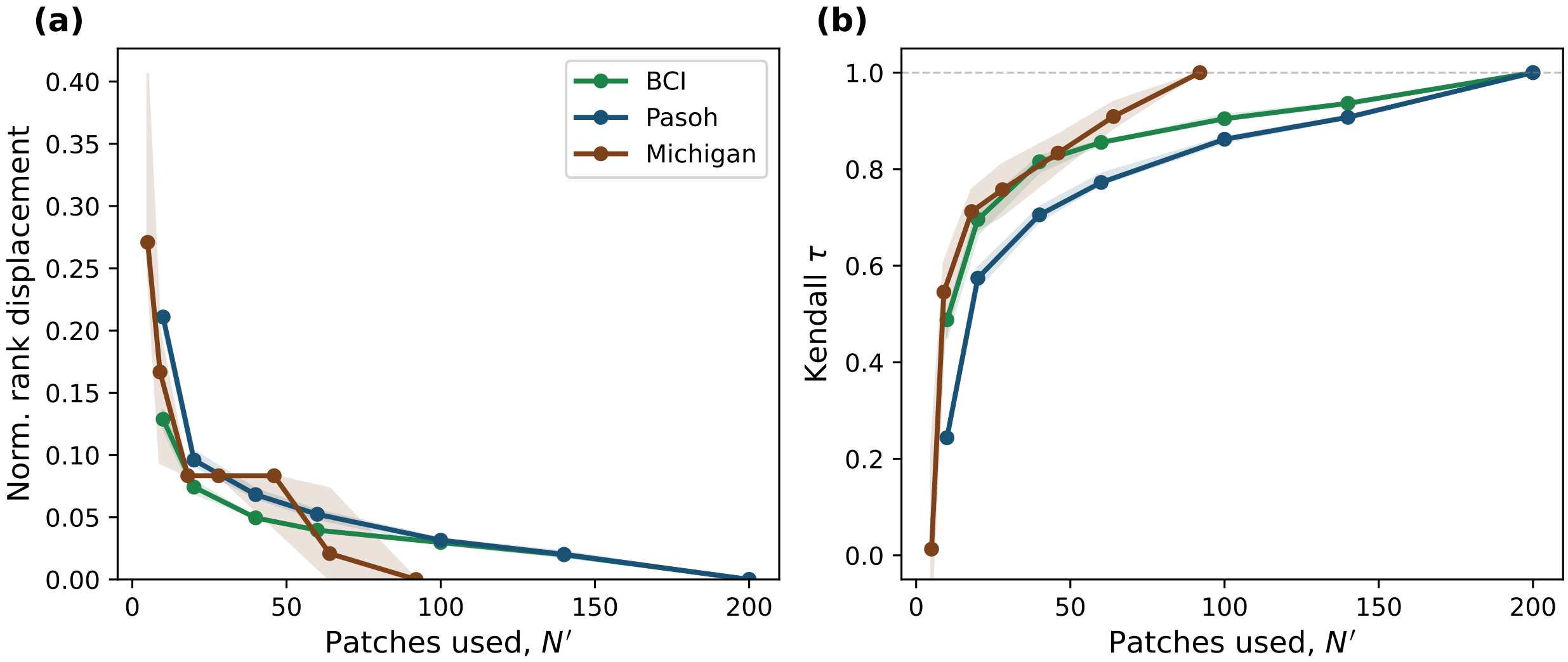

Supplementary Figure 14: **Vulnerability ranking converges rapidly with census size.** For each dataset at $L = 50\,\mathrm{m}$, the DMFT model was refitted on random subsamples of N' patches ($N'/N \in \{0.05, \ldots, 1.0\}$, $n_{\mathrm{rep}} = 10$ per fraction), and the resulting W_α ranking was compared to the full-census reference. **(a)** Normalised rank displacement $\langle |\mathrm{rank}_{\mathrm{sub}} - \mathrm{rank}_{\mathrm{ref}}| \rangle / S$, measuring the average shift in rank position (values near zero indicate high agreement). **(b)** Kendall rank correlation τ between subsample and full-census rankings; $\tau = 1$ (dashed) indicates perfect agreement. Both metrics converge rapidly with N': rank displacement falls below 0.05 at $N' \approx 60$ patches for BCI and Michigan, and ≈ 100 for Pasoh; $\tau > 0.85$ is reached at similar thresholds. Lines show medians and shaded bands interquartile ranges. BCI and Pasoh have $N = 200$ patches at $L = 50\,\mathrm{m}$; Michigan has $N = 92$. These thresholds match those of the CvM proxy (Supplementary Figure 13), indicating that parameter convergence and ranking stability are governed by the same data requirements.

We quantify ranking agreement using two complementary statistics (Supplementary Figure 14). The *normalised rank displacement* $\langle |\mathrm{rank}_{\mathrm{sub}} - \mathrm{rank}_{\mathrm{ref}}| \rangle / S$ measures the average deviation of each species from its full-data rank: a value of 0.05 means species are displaced by $\sim 5\%$ of the total species list, indicating a nearly unchanged ranking. The *Kendall rank correlation* τ measures overall rank agreement, ranging from -1 (reversed ranking) to $+1$ (perfect agreement); values $\tau > 0.85$ indicate strong monotone agreement between subsample and full-data rankings.

The DMFT vulnerability ranking converges rapidly with census size (Supplementary Figure 14). Median rank displacement **(a)** drops below 0.05 by $N' \approx 60$ patches for BCI and Michigan, and by $N' \approx 100$ patches for Pasoh, indicating that even moderate subsamples suffice to place species close to their full-data rank. The Kendall τ **(b)** confirms this picture: median $\tau > 0.85$ is reached at $N' \approx 60$ patches for BCI ($N'/N = 0.3$) and for Michigan ($N'/N \approx 0.7$), and at $N' \approx 100$ patches for Pasoh ($N'/N = 0.5$); $\tau \to 1$ at the full census by construction.

These thresholds coincide with the patch counts at which the CvM proxy converges (Supplementary Figure 13), indicating that parameter convergence and ranking convergence are governed by the same sample-size requirement.

8 Statistical relation between vulnerability and spatial variability

Supplementary Figures 15 through 18 provide details on the statistical relationships between mean abundance, variability in abundance distribution, and species vulnerability. We performed fits on log-transformed variables to account for the nature of the fractional abundances $p_{\alpha i}$ (the fraction of site i occupied by species

α), which, due to the constraint $\sum_{\alpha=1}^{S} p_{\alpha i} \leq 1$ and the large number of species considered, are generally very close to zero. Since $W_\alpha \leq 0$ by definition, we use $|W_\alpha|^{-1}$ as a more intuitive response variable for the vulnerability regressions. This quantity is positive and thus log-transformable; it also has a natural interpretation as a proximity-to-threshold measure, increasing as a species approaches the critical point $W_\alpha \to 0^-$.

Supplementary Figure 15 demonstrates a clear negative correlation between $|W_\alpha|^{-1}$ and the spatial heterogeneity of abundance (σ_{p_α}): species with higher spatial variability are more buffered (larger $|W_\alpha|$, smaller $|W_\alpha|^{-1}$). This pattern is consistent across datasets and simulations, underscoring a robust relationship between local abundance fluctuations and overall species vulnerability.

Supplementary Figure 16 extends this analysis by examining the link between $|W_\alpha|^{-1}$ and mean stem abundance ($\langle n_\alpha \rangle$). Again, we observe a negative correlation, suggesting that species with lower average abundances tend to be more vulnerable. This is an intuitive finding. However, we note that the regression coefficient is smaller than in the previous regressions in both datasets and simulations.

While Supplementary Figure 15 shows that spatial heterogeneity has a stronger correlation with vulnerability than mean abundance, Supplementary Figure 17 reveals a strong positive correlation between the two regressors, σ_{p_α} and $\langle n_\alpha \rangle$. This raises the question of whether the observed negative correlation between $|W_\alpha|^{-1}$ and σ_{p_α} might be an indirect consequence of their mutual correlation with mean stem abundance.

Supplementary Figure 18 addresses this potential confounding effect by performing a partial correlation analysis. For BCI and Pasoh, the partial correlation is strongly negative and highly significant (see table in Supplementary Figure 18), confirming that the association between $|W_\alpha|^{-1}$ and σ_{p_α} survives after controlling for mean stem abundance $\langle n_\alpha \rangle$. For Michigan, the partial correlation is not statistically significant ($n = 12$ species), reflecting insufficient power to detect the effect with a small species sample. Overall, the two large tropical-forest datasets support the interpretation that local spatial variability is an independent driver of species vulnerability beyond what rarity alone predicts.

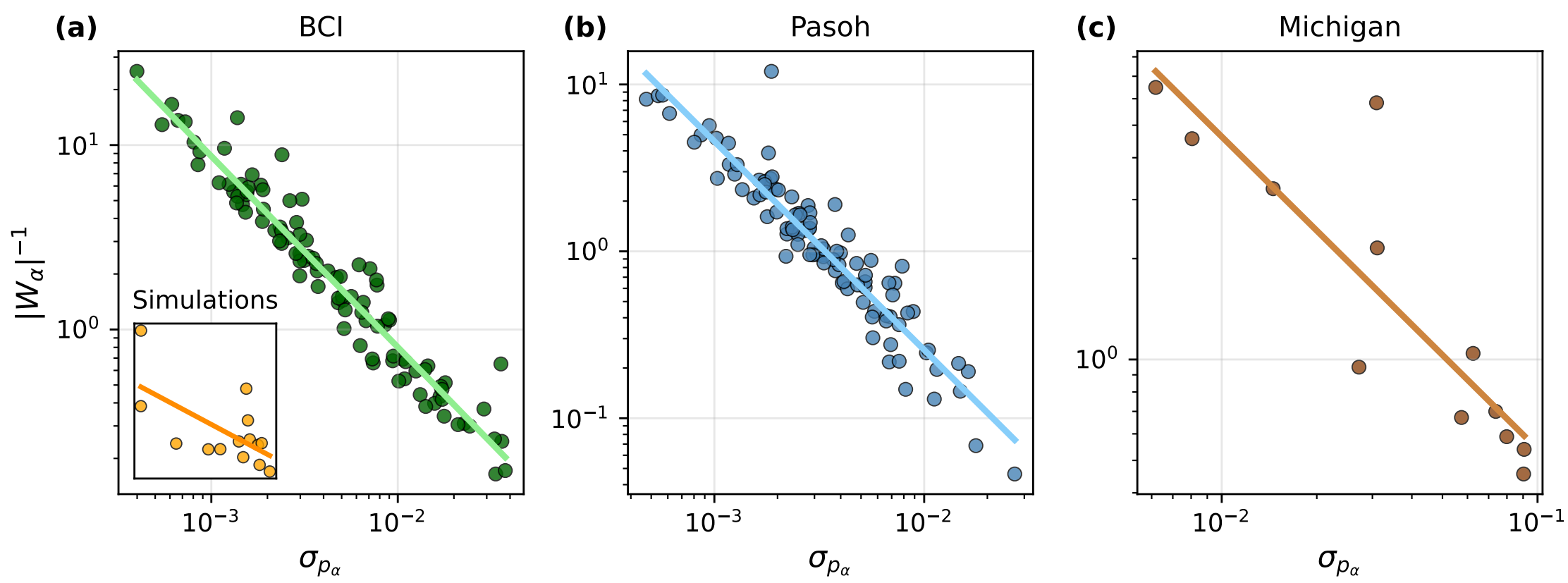

Regression: $\log |W_\alpha|^{-1}$ vs. $\log \sigma_{p_\alpha}$				
	Simulations	BCI	Pasoh	Michigan
Slope	-0.189	-1.036	-1.250	-0.932
95% CI	[-0.334, -0.044]	[-1.089, -0.982]	[-1.336, -1.164]	[-1.287, -0.577]
Slope p-value	0.0151	5.5e-61	1.02e-49	0.000162
R^2	0.401	0.938	0.895	0.774
n	14	100	100	12

Supplementary Figure 15: **Species vulnerability is negatively correlated with spatial heterogeneity.** Log-log regression of $|W_\alpha|^{-1}$ versus spatial heterogeneity in abundance (σ_{p_α}). Since $W_\alpha \leq 0$, the quantity $|W_\alpha|^{-1}$ is positive and log-transformable; larger values indicate higher vulnerability (proximity to the critical threshold $W_\alpha = 0$). Each panel refers to a dataset or numerical simulations, as indicated by the label. The inset shows the simulation results. The table presents the main regression statistics: slope, p-value of the slope, R^2, and number of samples n. A negative slope indicates that more spatially heterogeneous species are less vulnerable.

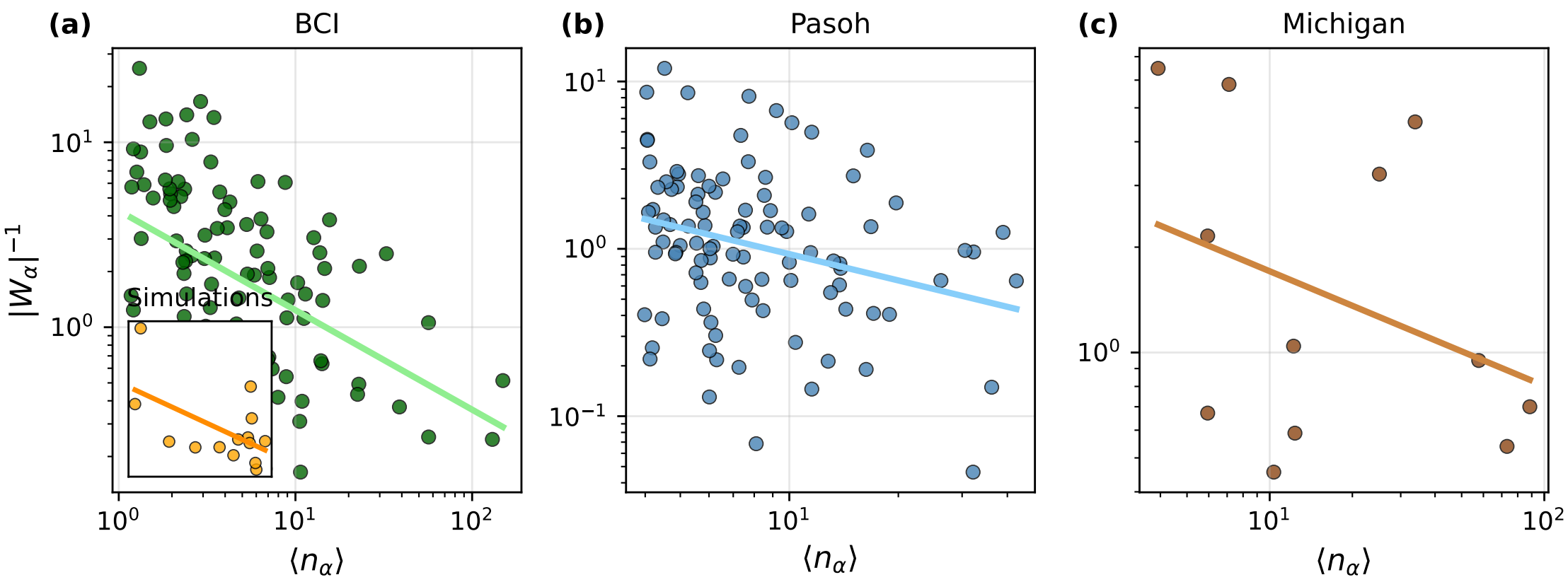

Regression: $\log \lvert W_\alpha \rvert^{-1}$ vs. $\log\langle n_\alpha \rangle$				
	Simulations	BCI	Pasoh	Michigan
Slope	-0.163	-0.538	-0.524	-0.327
95% CI	[-0.316, -0.010]	[-0.735, -0.340]	[-0.879, -0.168]	[-0.940, +0.286]
Slope p-value	0.0383	4.72e-07	0.00433	0.261
R^2	0.311	0.229	0.080	0.124
n	14	100	100	12

Supplementary Figure 16: **Species vulnerability is negatively correlated with mean stem abundance.** Same layout as Supplementary Figure 15 but for $|W_\alpha|^{-1}$ versus mean stem abundance $\langle n_\alpha \rangle$. A negative slope indicates that more abundant species are less vulnerable.

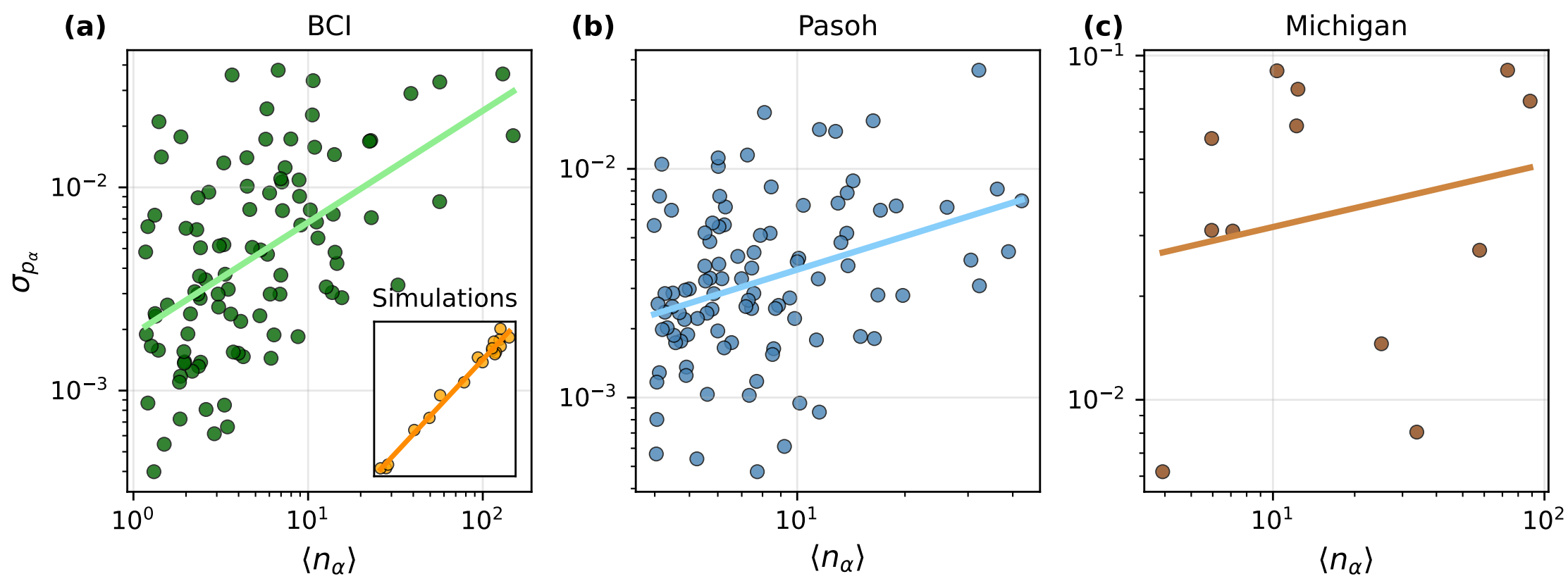

Regression: $\log \sigma_{p_\alpha}$ vs. $\log\langle n_\alpha \rangle$				
	Simulations	BCI	Pasoh	Michigan
Slope	+0.978	+0.549	+0.487	+0.182
95% CI	[+0.915, +1.041]	[+0.369, +0.729]	[+0.224, +0.750]	[-0.422, +0.787]
Slope p-value	4.05e-16	2.46e-08	0.000389	0.517
R^2	0.985	0.273	0.121	0.043
n	18	100	100	12

Supplementary Figure 17: **Spatial abundance variability is positively correlated with mean stem abundance.** Same as Supplementary Figure 15 and 16 but for σ_{p_α} versus mean stem abundance, $\langle n_\alpha \rangle$.

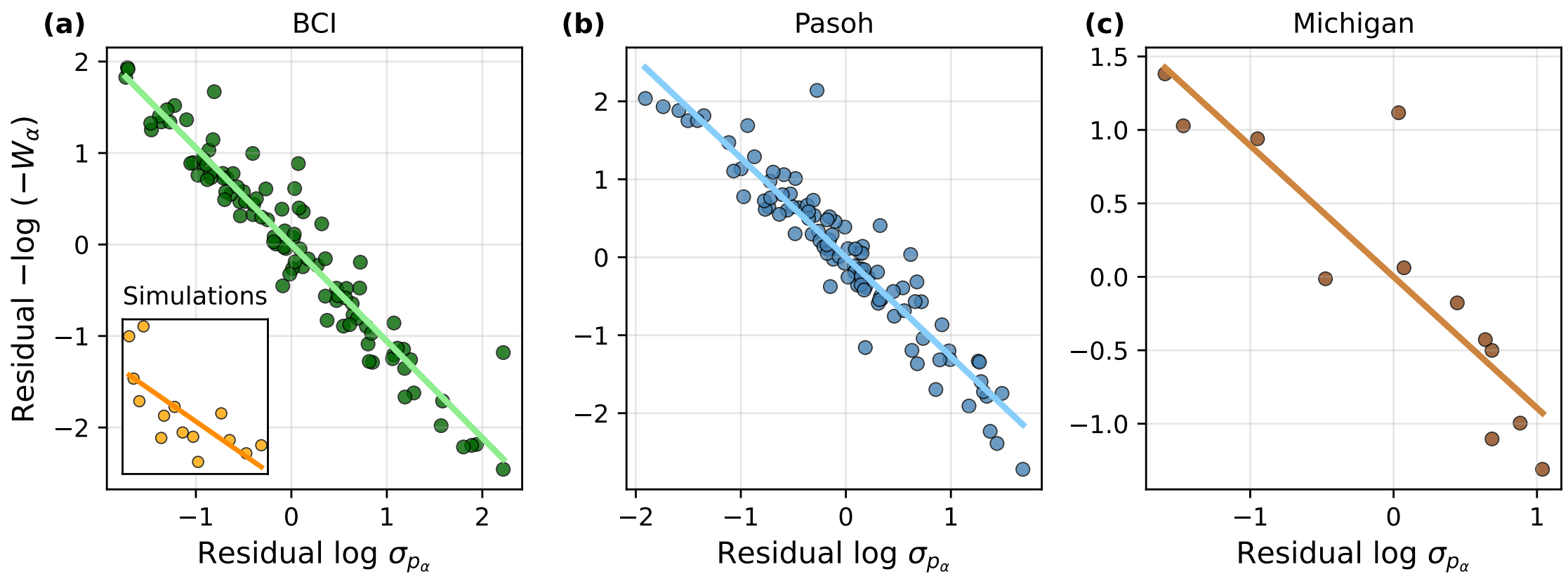

Partial regression: residual $-\log(-W_\alpha)$ vs. residual $\log \sigma_{p_\alpha}$				
	Simulations	BCI	Pasoh	Michigan
Slope	-1.323	-1.057	-1.274	-0.893
95% CI	[-2.123, -0.524]	[-1.119, -0.995]	[-1.365, -1.183]	[-1.231, -0.555]
Slope p-value	0.0036	8.43e-56	1.94e-48	0.000154
R^2	0.520	0.921	0.888	0.776
n	14	100	100	12

Supplementary Figure 18: **Species vulnerability is negatively correlated with spatial heterogeneity, even accounting for shared correlations through the mean.** Partial correlation (added-variable plot) between the residuals of $\log |W_\alpha|^{-1}$ vs. $\log\langle n_\alpha \rangle$ and the residuals of $\log \sigma_{p_\alpha}$ vs. $\log\langle n_\alpha \rangle$, both in log-space. A negative slope confirms that the association between $|W_\alpha|^{-1}$ and σ_{p_α} survives after controlling for mean stem abundance $\langle n_\alpha \rangle$.

References

[1] F. Simini, T. Anfodillo, M. Carrer, J. R. Banavar, A. Maritan, Self-similarity and scaling in forest communities. *Proceedings of the National Academy of Sciences* **107** (17), 7658–7662 (2010), doi:https://doi.org/10.1073/pnas.1000137107.

[2] I. Martínez Cano, H. C. Muller-Landau, S. J. Wright, S. A. Bohlman, S. W. Pacala, Tropical tree height and crown allometries for the Barro Colorado Nature Monument, Panama: a comparison of alternative hierarchical models incorporating interspecific variation in relation to life history traits. *Biogeosciences* **16** (4), 847–862 (2019), doi:https://doi.org/10.5194/bg-16-847-2019.

[3] J. Terborgh, The Vertical Component of Plant Species Diversity in Temperate and Tropical Forests. *The American Naturalist* **126** (6), 760–776 (1985), doi:https://doi.org/10.1086/284452.

[4] R. D'Andrea, *et al.*, Counting niches: Abundance-by-trait patterns reveal niche partitioning in a Neotropical forest. *Ecology* **101** (6), e03019 (2020), doi:https://doi.org/10.1002/ecy.3019.

[5] L. S. Comita, S. Aguilar, R. Pérez, S. Lao, S. P. Hubbell, Patterns of woody plant species abundance and diversity in the seedling layer of a tropical forest. *Journal of Vegetation Science* **18** (2), 163–174 (2007), doi:10.1658/1100-9233(2007)18163:powpsa2.0.co;2.

[6] A. Lindén, S. Mäntyniemi, Using the negative binomial distribution to model overdispersion in ecological count data. *Ecology* **92** (7), 1414–1421 (2011), doi:10.1890/10-1831.1.

[7] R. B. O'Hara, D. J. Kotze, Do not log-transform count data. *Methods in Ecology and Evolution* **1** (2), 118–122 (2010), doi:10.1111/j.2041-210X.2010.00021.x.

[8] X. A. Harrison, Using observation-level random effects to model overdispersion in count data in ecology and evolution. *PeerJ* **2**, e616 (2014), doi:10.7717/peerj.616.

[9] O. H. Diserud, S. Engen, A General and Dynamic Species Abundance Model, Embracing the Lognormal and the Gamma Models. *The American Naturalist* **155** (4), 497–511 (2000), doi:10.1086/303339.

[10] S. P. Hubbell, *The Unified Neutral Theory of Biodiversity and Biogeography*, vol. 32 of *Monographs in Population Biology* (Princeton University Press) (2001), doi:10.1515/9781400837526.

[11] I. Volkov, J. R. Banavar, S. P. Hubbell, A. Maritan, Neutral theory and relative species abundance in ecology. *Nature* **424** (6952), 1035–1037 (2003), doi:10.1038/nature01883.
