# Supplementary material for: Dispersal diversity buffers species vulnerability to local extinction
Source: Nat Ecol Evol. 2026 Oct 9;10(11):2060–73. doi: 10.1038/s41559-026-03195-y (PMC13736521; doi:10.1038/s41559-026-03195-y)
Supplement: Supplementary file 3 [file 41559_2026_3195_MOESM3_ESM.pdf]

nature portfolio

Peer Review File

Dispersal diversity buffers species vulnerability to local extinction

Corresponding Author: Dr Davide Bernardi

Version 0:

Decision Letter:

23rd December 2025

*Please ensure you delete the link to your author homepage in this e-mail if you wish to forward it to your co-authors.

Dear Dr Bernardi,

Thank you for your patience while we sought reviewer feedback on your Article, "Rare but Resilient: Dispersal diversity buffers vulnerability in rare species". Unfortunately we were unable to obtain the report of one of the reviewers who originally agreed to review you manuscript, which has led to some delay. My apologies for not keeping you updated sooner on this. However, I can confirm that it has has now been seen by 2 reviewers. You will see from their comments copied below that while they find your work of considerable potential interest, they have raised quite substantial concerns that must be addressed. In light of these comments, we cannot accept the manuscript for publication, but would be very interested in considering a revised version that addresses these serious concerns.

We hope you will find the reviewers' comments useful as you decide how to proceed. If you wish to submit a substantially revised manuscript, please bear in mind that we will be reluctant to approach the reviewers again in the absence of major revisions.

In particular, Reviewer #1 feels that the VAAD approach needs to be outlined much more clearly, and compared to existing approaches; while Reviewer #2 feels that the research needs to be much better situated in modern theory, and facilitation incorporated into the model, which they feel is currently downplayed.

If you choose to revise your manuscript taking into account all reviewer and editor comments, please highlight all changes in the manuscript text file [OPTIONAL: in Microsoft Word format].

We are committed to providing a fair and constructive peer-review process. Please do not hesitate to contact us if there are specific requests from the reviewers that you believe are technically impossible or unlikely to yield a meaningful outcome.

If revising your manuscript:

* Include a “Response to reviewers” document detailing, point-by-point, how you addressed each referee comment. If no action was taken to address a point, you must provide a compelling argument. This response will be sent back to the referees along with the revised manuscript.

* If you have not done so already we suggest that you begin to revise your manuscript so that it conforms to our Article format instructions at http://www.nature.com/natecolevol/info/final-submission. Refer also to any guidelines provided in this letter.

* Include a revised version of any required reporting checklist. It will be available to referees (and, potentially, statisticians) to aid in their evaluation if the manuscript goes back for peer review. A revised checklist is essential for re-review of the paper.

Please use the link below to submit a revised paper:

Link Redacted

Note: This URL links to your confidential home page and associated information about manuscripts you may have submitted, or that you are reviewing for us. If you wish to forward this email to co-authors, please delete the link to your homepage.

If you wish to submit a suitably revised manuscript we would hope to receive it within 6 months. If you cannot send it within this time, please let us know. We will be happy to consider your revision so long as nothing similar has been accepted for publication at Nature Ecology & Evolution or published elsewhere.

Nature Ecology & Evolution is committed to improving transparency in authorship. As part of our efforts in this direction, we are now requesting that all authors identified as ‘corresponding author’ on published papers create and link their Open Researcher and Contributor Identifier (ORCID) with their account on the Manuscript Tracking System (MTS), prior to acceptance. This applies to primary research papers only. ORCID helps the scientific community achieve unambiguous attribution of all scholarly contributions. You can create and link your ORCID from the home page of the MTS by clicking on ‘Modify my Springer Nature account’. For more information please visit please visit www.springernature.com/orcid.

Please do not hesitate to contact me if you have any questions or would like to discuss the required revisions further.

Thank you for the opportunity to review your work.

[redacted]

Reviewer expertise:

Reviewer #1: Theoretical community ecology

Reviewer #2: Theoretical community ecology

Reviewers’ comments:

Reviewer #1 (Remarks to the Author):

This model introduces new theoretical models for species dispersal and competition, alongside novel ways to quantify the success of these models. The approach is tested at two tropical forest plots, and fits to data for both the novel distributions, and also the prediction of whether all species _would_ be predicted to coexist, are impressive.

There is something new and important here that is working well, but my two major comments are as follows, and both relate to a deeper understanding of what the model is getting right (and doing better than alternative models), as well as a clearer understanding what type of data is being used to parameterize the species-specific distributions.

(1) To my knowledge the VAAD (the vacancy-adjusted abundance distribution) is introduced here---or if it is in other papers by the authors my understanding is that this must be fairly recently introduced. The success of the model is tested in part by the matches between predicted and observed VAADs, and these are impressive. However, it took me a while to grasp both what this distribution was, and also its significance (and I am still not sure of the latter).

In short, the VAAD depends critically on how empty a local patch is relative to how many individuals it COULD support. Thus even if in two patches a given species has the same relative abundance, if one patch is half full, while the other is maxed out, these two will contribute differently to the VAAD for that species. This differs from most approaches that wouild be based on relative abundance, independent of how full or empty a patch is. So this is an important idfference, that I think should really be brought front and center, and not relegated to one among many quantities defined in a box. The authors perhaps should put a clearer explanation in the main text when it is first mentioned.

That being said, I am still trying to get my head around what this vacancy-adjustment means and how it should be intepreted. There is a lot of interesting recent work at BCI and in other forests on gap dynamics. Some of this is referenced here, but perhaps more literature could be added. These gap dynamics highlight the differences in the way species with different life history strategies (e.g. pioneers vs shade tolerant) respond to gaps---specifically that pioneers will grow quickly to fill gaps. So....the vacancy-adjustment seems to have a life history implication, such that some types of species (in terms of their life history strategies) may have quite different vacancy-adjusted abundances vs non-vacancy adjusted, while for others these may be much more similar But this was not quite spelled out and so I was not sure how to go further than that.

SO there is clearly something interesting there, and I am asking the authors to spell that out, and put it in context of other work related to gaps, life history variation across tropical trees, and ways to quantify each of these.

That being said, I had two major question marks here, both relating to the definition of "vacancy". Once we are thinking about life history differences, and the speed at which a species will fill in gaps, etc, we are inevitably talking about stage or size-structured populations. But then, that means that not all abundances in a sublot are comparable. Some subplots may have many small, young trees, while others may have fewer, odler, larger trees. But they may have the same amount of space actually physically "vacant". So may question here is whether abundance is even the correct currency to capture what the authors are trying to do, or whether they may want to take into account weighting this with basal area or something similar.

Related, but slightly different, as mentioned above the vacancies are defined relative to the maximum number of individuals observed in a subplot. There is then the issue above wrt how big are the individuals in different subplots. But there is another issue. Forest Dynamics plots are chosen to be relatively homogeneous, but there could still be fundamental differences in productivity between different subplots due to soil, topography, or some other unknown factor. So this makes me question in a different way just how easily we can know the true amount of vacancies in a given subplot, which is criticlaly important for defining the VAAD.

(2) The VAAD fits were impressive, but there were also several other quantities that seemed to match the authors' model very well. This is a species-specific model, so in principle there are a large number of parameters, unlike (for example) a neutral model. As far as I can tell, many of these parameters are fixed by the data, though what was unclear to me was exactly what aspects of the data are being used to parametrize the models, and how independent is this data used for fitting the models from the data being used to test the model outcomes. Relatedly, are any model parameters left as truly free parameters, that are then tuned to fit the data? Both of these points could be made clearer.

(3) It may not have been the goal of the authors, but I wonder if they have used their model to make predictions for temporal fluctuations, or other kinds of temporal predictions.

(4) Finally, to put the results in context, I would like to see a comparison with alternative models that could potentially predict the same quantities. For example, this might be a neutral model, or a model with species-specific stage-structure or gap dynamics, or something else that is plausibly related to the authors' model. Seeing how much better this model performs, and also (imprortantly) comparing the data overhead of exactly how much data is needed to parametrize those alternative models versus this one, would help put the results in context.

Reviewer #2 (Remarks to the Author):

Overall, I really enjoyed this paper and think it is a cool model. There are a couple of major issues, however, that I feel need to be resolved, but I think all, except possibly one (facilitation) can be dealt with in revision of the text.

1. Overall, I do not think you have sufficiently or accurately placed your work in the context of the relevant modern literature. You set up the premise of this paper by citing classic papers, ignoring the large body of literature that has been trying to add biological realism to both coexistence and spatial process modelling in recent years. This needs to be resolved. These are the concepts that you suggest you are the first to think about with this paper, that have definitely been examined, if using different approaches in the literature: coexistence as a probability or feasibility domain, spatially explicit coexistence, spatially explicit extinction risk, spatially explicit models of community diversity and abundance patterns that account for species traits and vital rates. What is new in this paper is your approach. I know you have a limit on references, but I think you can easily get rid of some of the less relevant in lieu of the more relevant ones I suggest here or that you find upon more careful research of the recent literature. Please have a look at these references and revise your introduction to make it more accurate about you adding to a growing literature seeking to add biological realism to models of community diversity and extinction probabilities.
a. D. Garcıa-Callejas, I. Bartomeus, and O. Godoy. The spatial configuration of biotic interactions shapes coexistence-area relationships in an annual plant community. Nature Communications 12.1 (2021), p. 6192.
b. Ellner, S. P., Snyder, R. E., Adler, P. B., & Hooker, G. (2019). An expanded modern coexistence theory for empirical applications. Ecology letters, 22(1), 3-18
c. Grilli, J., Adorisio, M., Suweis, S., Barabás, G., Banavar, J. R., Allesina, S., & Maritan, A. (2017). Feasibility and coexistence of large ecological communities. Nature communications, 8(1), 14389.
d. Grainger, T. N., & Gilbert, B. (2016). Dispersal and diversity in experimental metacommunities: linking theory and practice. Oikos, 125(9), 1213-1223. (This one is particularly relevant as background for your study)

From the spatial perspective you have also over-simplified the existing literature – there is a lot of effort to add biological realism in joint-species distribution models (For example: Harris, D. J. (2015). Generating realistic assemblages with a joint species distribution model. Methods in Ecology and Evolution, 6(4), 465. There are lots others)
e. Keil, P., Wiegand, T., Tóth, A. B., McGlinn, D. J., & Chase, J. M. (2021 . Measurement and analysis of interspecific spatial associations as a facet of biodiversity. Ecological Monographs, 91(3), e01452.
f. Wiegand, T., Grabarnik, P., & Stoyan, D. (2016). Envelope tests for spatial point patterns with and without simulation. Ecosphere, 7(6), e01365.

2. One of the big changes in how coexistence is being modelled these days is a shift away from overly simplistic direct competition models to those that allow for complex multispecies interactions and facilitation. As far as I can tell you have done neigther of these things. This is a really cool model but to me the exclusion of facilitation is a problem, and one that does possibly impact your results. In particular, I would like to know if the inclusion of facilitation would improve your model fit at the smallest spatial scales. These are the scales at which I would expect to see strong facilitation so it makes sense these are the scales at which your model fits least well. I can't tell from your methods if you have omitted facilitation for mathematical reasons) – most Lotka-Volterra based models break if you all facilitation, or if it is because you decided it doesn't matter but you need to try. Facilitation is extremely common in tropical forests, so I don't accept the argument that this is a minor and uncommon process and thus can be ignored, especially as there is a pattern that seems like it could result from that omission. As I comment on below, I really cannot tell how you incorporated competition into your model but the exponential plant population growth model in (M. Mayfield and D. Stouffer, 2017. 2017 Higher-order interactions capture

unexplained complexity in diverse communities. Nature Ecology and Evolution 1(3):006 DOI: 10.1038/s41559-016-0062 is one way to do this, though it may not be relevant for your model – I can't tell. at very least, explain how its inclusion is likely to change your results. Here are some relevant papers on facilitation and why it is so important to include.

1. G. Losapio et al. "Network motifs involving both competition and facilitation predict diversity in alpine plant communities. In: Proceedings of the National Academy of Sciences 118.6 (2021), e2005759118.
2. B. Pichon et al. The interplay of facilitation drives the emergence of multistability in dryland plant communities. In: Ecology (2024), e4369
3. E. J. McIntire and A. Fajard . Facilitation as a ubiquitous driver of biodiversity. New phytologist 201.2 (2014), pp. 403–416.
4. L. Buche et al. A continuum from positive to negative interactions drives plant species' performance in a diverse community. Ecology Letters 28.1 (2025), e70059.
5. M. Bimler, M. Mayfield, and A. James, 2025. Facilitation thinking for coexistence theory. Ecology Letters 28 (6), e70150.

3. I think your model is very interesting, but the idea that trait ratios are important for determining the fate of species in communities is not new – again, I want to see a discussion (brief) of how your model adds to and moves beyond these past studies in this research area that have also used trait differences to explain communities.
1. D. Tilman. The resource-ratio hypothesis of plant succession. The American Naturalist (1985), pp. 827–852
2. M. Mayfield and J. Levine. 2010 Opposing effects of competitive exclusion on the phylogenetic structure of communities. Ecology Letters. 13:1085-1093

4. Your general modelling approach seems to align with other recent models of coexistence that look at coexistence as a probability distribution or as a feasibility domain. Please address in your paper how your model fits within this approach to coexistence modelling – advances it or just increases its usefulness, acknowledge it all the same. I think yours is different but you need to acknowledge that you are not the first group to think about coexistence this way.
1. J. W. Spaak and F. De Laender. Intuitive and broadly applicable definitions of niche and fitness differences. Ecology Letters 23.7 (2020), pp. 1117–1128.
2. C. Bowler, C. Weiss-Lehman, I. Towers, M. Mayfield, and L G Shoemaker 2022. Determining the probability of coexistence: Adding demographic variation to modern coexistence modelling. Ecology Letters 25(7): 1618-1628

5. For my taste, you have not explained your framework well enough to understand what it means biologically. Particularly, the first part where you add in the "biology" it is unclear how you modelled, measured or general "get" the vital rate and interaction coefficients. You might argue that it doesn't matter because the outcome works but models of these vital rates are notoriously basic and reliant on a major assumptions that tend to work in certain types of ecosystems (totally high diversity rainforests) and not others. After reading the methods, I still don't know what biology your model is based on. I came away in the end thinking this is a neutral model and ended with the question, what does this do for us that Hubbell's neutral model does not? Further, the test systems you use are hyper diverse rainforests, one of the few systems that Hubbell's neutral model works in as well. I would be more convinced that this provides useful results for a wide range of systems if you had tested two different types of plant systems - even a temperate forest. What to do about this? Well I want to see the methods explain what biology is in here and how you added species specific biology in. I would love you to use a system other than a rainforest as an empirical test – the rainforests are fine, don't get me wrong, but with an ecological literature that has clearly shown the rainforest diversity behaves differently from the diversity in other systems, this test alone is great but not good enough to convince me that this model will always come to the "right answer." Also, I am unconvinced that many empirical ecologists will use this method unless you can explain how you parameterised the vital rates and competition in your model.

Minor comments
Title: I don't think the title aligns with your paper. Rare species seem a minor part of this study, why is that highlighted in the title?

Line 26 – I would change "diverse ecosystems" to a "range of different ecosystems" for clarity, the original phrasing can mean two things so might confuse some readers whether you mean any diverse ecosystem or a diverse range of ecosystems.

Lines 36-40 - though you have referenced a lot of papers here, they are all focusing on more traditional paradigms of coexistence. You mention briefly that simplifications are required but you don't cite any of the recent work attempting to increase the complexity of coexistence and diversity model. I see this as problematic, especially as you set up the argument that most models are too simplistic but you have yet to acknowledge those that have attempted to add in complexity (a few suggested references in my major comment section).

Lines 43-44 – the discussion of the Red List seems odd and out of place here. I guess you are trying to link your paper to conservation but I think it is not necessary and this seems like a clumsy straw man – the Red List is notoriously over simplified (by necessity) but definitely does not represent the cutting edge of species distribution modelling. Maybe you come move this to the discussion as a potential application of this model?

Lines 46 – 48 as per your list of papers on the coexistence literature, I feel you have focused on very traditional approaches (see a few suggestions in the major comment section).

Line 61 – please define "vulnerability" clearly here – I am sure you are going to get into it, but so much of your paper depends on this term and how you define it, I think it is really important you are clear and upfront about what this means from

the very start.

Line 63 – why no facilitation? I would think the presence of facilitation, which is a spatially structured process (Bimler et al. 2018) would make a big different to vulnerability.

Table 1 is awesome, I love a glossary and appreciate the inclusion of this table. My only complaint is your Table legend is too brief – it should be clear what is in the table without reading the whole text.

Line 73 – watch for terms with double means in the context of ecology. I would not use "viability" on this line as its meaning is ambiguous. Reading the rest of the sentence, I don't know what you mean by viability – viability of meta-communities?

Line 77 – again – do you really mean evolution? Or do you mean the development or assembly? I would not use the word evolution unless you mean it biologically.

Line 90 – how is this species specific without data? I am sure you explain this in the methods (well not really as I have now read the methods) but could you add a sentence explaining the general approach?

Figure 1 – your observations seem to get much less accurate at smaller L's. I would like to see if the inclusion of facilitation explains this – it is these smaller scales where you would expect this effect to be strongest.

OK really hard to understand what the VAA does without reading the methods, I know word limits are tough but you need to explain this a bit more here.

Lines 126-128 – Does your method add up each species competitive balance? Or does it allow for non-linear relationships to emerge? One of the biggest problems with current community models is they assume that communities are the sum of the ecologies of individual species – how does your method move beyond that? This is unclear

Figure 2 – What is the point of Panel F? How do these plots connect to A-E, not clear from your legend. Is it just the point that you can have different dispersal kernals and the same abundance? I am not convinced this is a great way to make this point, especially since the abundances are not actually that similar. If you want to keep these, maybe a solution would be to draw an arrow linking the point in E that represents these two species to make the connection clear (or with symbols like in Figure 3 – I like the clear link between panel E and F in Figure 3 more than in Figure 2?) Panel E – the spread seems to shrink as abundances increase - I know is this not your point but why is that?

Line 209 - I am not completely convinced this is true – at very small spatial scales you see a deviation – it is these small spatial scales at which individuals interact so I think this deviation is really-important, not something to be brushed aside. I want to see you spend more time exploring that deviation.

Line 229 – again, I don't think these abundances are that similar. Either explain why these abundances are close enough for your purposes (somewhere, not necessarily here) OR use a different word – high relative abundances for instance?

Line 234 – Switch to first person: "We present a novel theoretical framework…." You are flip flopping from first to third person, which makes it harder to read. This sentence in general needs some work – how about: We present and test a novel theoretical framework with data from two species-rich tropical forest systems.

Line 235 – After this sentence add a sentence that states how this adds to the existing, growing modern literature on biologically realistic coexistence and extinction predictions.

Lines 253-257 – this is a nice thought, but I suspect the actuals associated with doing this would be quite difficult, so perhaps rephrase here to make it clear that this is a far distant extension, not something you could just pick this framework up and do tomorrow.

Line 259 – why are you suddenly bringing up evolution? I would think the literature on the spatial dependency of coexistence and (if you want) the evolution of coexistence would be a more relevant point to make here. Yours study does not tell us anything about evolutionary processes. Either cut this or change it to match the rest of your paragraph. Here are two papers – one showing the spatial dependency of coexistence and the other the evolution of coexistence (though, again, I am not convinced that is relevant).
1. Bimler, M. D., Stouffer, D. B., Lai, H. R., Mayfield, M. M. 2010. Accurate predictions of coexistence in natural systems require the inclusion of facilitative interactions and environmental dependence. Journal of Ecology 106(5): 1839-1852.
2. Pastore, A. I., Bimler, M. D., Barabás, G. Mayfield, M. M., Miller, T. E. 2021. Towards a theoretical understanding of changes in niche overlap and competitive difference . When can coexistence evolve? Nature Ecology and Evolution 5(3): 330-337.

Lines 277-278 – again, you have really under referenced this concern, I know you have reference limits but you can definitely swap out some from your introduction with those that I recommend throughout my review to make your paper more clearly align with the relevant literature.
1. Comins, H. N., & Noble, I. R. (1985). Dispersal, variability, and transient niches: species coexistence in a uniformly variable environment. The American Naturalist, 126(5), 706-723.
2. Lavorel, S., O'Neill, R. V., & Gardner, R. H. (1994). Spatio-temporal dispersal strategies and annual plant species

coexistence in a structured landscape. Oikos, 75-88.

Line 283 – this is not a sufficient mention of facilitation, and though mutualisms may be rare, facilitation is not.

Methods:
Lines 461-47 I don't feel there is enough detail here for most readers to understand what you are doing. For instance:
"The model tracks the time evolution of the fraction of site $i = 1 \ldots N$ occupied by species α, denoted $p\alpha i$, driven by local birth/death processes, colonization, and interspecific competitio . Being defined as a fraction, $0 \leq$ Í$S\alpha$ =1 $p\alpha i \leq 1$ for all sites, which also implies $0 \leq p\alpha i \leq 1$ for all i and α."

I can see that S = the number of species. But what is the input data for α? I can see that pαi is a function of birth/death processes, colonisation and interspecific competition but you don't explain what those functions are. In the main text you say you don't need a lot of empirical data, but what do you need? The only part of this that I can follow is the spatial element.

Though I can deduce that how you measured birth and death to some extent, there are a wide range of commonly used competition models, Beverton-Holt, Linear Lotka Volterra, Ricker…. What model are you using? Any of these? Or are you using a different approach entirely?

Next, what IS K? you say it is a species-specific kernal, but where does this come from? Is it generated from data? Is it randomly assigned from a distribution? Explain how not just what each of these model variable/functions is.

You need to explain this well enough that the reader understands the underlying models without interrogating your code or reading whole other papers.

Lines 478-480 – that is all great, but this referencing of other papers is not sufficient to explain your model, especially given that the point of this paper is to present a model that has tangible applications for empirical ecologists.

Line 481-482 – Again, you are giving us little glimpses of your framework but not the meat. Classical competition models don't do a very good job of explaining real plant communities so I am not particularly convinced that this is a good thing (see my request that you incorporate facilitation).

Line 495 – just to clarify – these are species specific but randomly assigned (no biological information), correct?

Line 512 – OK, great but how? Can you do a train and test sort of analysis here? Or insert real data to compare to your randomly assigned variables?

Lines 542-546 – Am I correct that this basically explains how you would go about the suggested next step on lines 253-257? If so, I would link this in the text to respond to my suggestion for additional explanation in the main text.

Line 580 - I still don't like the use of the word "evolving" to explain your model progression – please change throughout.

Reviewer #3 (Remarks on code availability):

I am sorry I don't have time to do this and as noted, some of the modelling aspects of this paper are beyond me so better to ask someone with better expertise in the physics modelling they used.

Version 1:

Decision Letter:

14th June 2026

Dear Dr. Bernardi,

Thank you for submitting your revised manuscript "Rare but Resilient: Dispersal diversity buffers species vulnerability" (NATECOLEVOL-25082966A). It has now been seen again by the original reviewers and their comments are below. The reviewers find that the paper has improved in revision, and therefore we'll be happy in principle to publish it in Nature Ecology & Evolution, pending minor revisions to satisfy the reviewers' final requests and to comply with our editorial and formatting guidelines.

If the current version of your manuscript is in a PDF format, please email us a copy of the file in an editable format (Microsoft Word or LaTex)-- we can not proceed with PDFs at this stage.

If you have not done so already, please ensure that you also email us a completed copy of the Reporting summary :

Reporting summary: <a href="https://www.nature.com/documents/nr-reporting-

summary.pdf">https://www.nature.com/documents/nr-reporting-summary.pdf

We are now performing detailed checks on your paper and will send you a checklist detailing our editorial and formatting requirements in a couple of weeks. Please do not upload the final materials and make any revisions until you receive this additional information from us.

Thank you again for your interest in Nature Ecology & Evolution. Please do not hesitate to contact me if you have any questions.

[redacted]

Reviewer #1 (Remarks to the Author):

I thank the authors for a comprehensive response to my comments and questions from the earlier draft. I think that the model and new way of computing diversity via the VAA could be highly impactful. The data overhead required to parametrize the model could be prohibitive in some sites, but that doesn't mean that where we have those data, the model isn't effective and impressive.

I have one additional comment. The authors clarified their approach to computing carrying capacity, M, nicely. And it is reassuring that M scales linearly with the choice of patch area across each of the three sites. However, I did not see this scaling with L reported in Section 4 of the Supplementary information, as stated in the response letter (this section deals with the question of how heterogeneous might be the carrying capacity across each of the sites).

Apologies if I did miss it somewhere else, but I think it would be useful to report the actual carrying capacities M across each location. When the currency of stem abundance is used, a nice check would be that M exceeds the most dense of the patches. When the currency of crown area is used, it should be that M scales roughly as n_layer*L^2, where n_layer is the expected number of vertical layers in each site. If this number is unreasonably large then that would be worth noting, and there is a growing literature on quantifying vertical layers that this coefficient could make contact with (e.g. Terborgh 1985 Am Nat, or more recently D'Andrea et al (2020) Ecology).

Reviewer #2 (Remarks to the Author):

Thank you for the thorough revision of your manuscript. I think the changes you have made make this a much stronger contribution, with clarifying text and analyses making your study more accessible to a wider audience. I am particularly pleased to see the addition of the temperate forest dataset and the train and test analyses, I think these strengthen your case for the generalisable value of this model a lot. Overall, I am very happy with this revised manuscript. I have made a few tiny suggestions on how to clarify the text in a couple of places - particularly in the abstract and introduction. The only thing I am still not satisfied with is your title. I still don't think it reflects the main message of your model and study. I have suggested a couple of alternative options. I don't expect you to use my suggestions but I do ask that you think about what title will attract the audience you are after. The focus on rarity still seems misplaced. My concern here is that you won't get as many people reading your paper with this title as if you used a title that highlighted the novelty of your model and the utility of it for capturing ecological processes effectively with regular and commonly available tree census data.

INTERNAL USE ONLY AIP modules required:["Life Science"]

Open Access This Peer Review File is licensed under a Creative Commons Attribution 4.0 International License, which permits use, sharing, adaptation, distribution and reproduction in any medium or format, as long as you give appropriate credit to the original author(s) and the source, provide a link to the Creative Commons license, and indicate if changes were made.
In cases where reviewers are anonymous, credit should be given to 'Anonymous Referee' and the source.
The images or other third party material in this Peer Review File are included in the article's Creative Commons license, unless indicated otherwise in a credit line to the material. If material is not included in the article's Creative Commons license and your intended use is not permitted by statutory regulation or exceeds the permitted use, you will need to obtain permission directly from the copyright holder.
To view a copy of this license, visit https://creativecommons.org/licenses/by/4.0/

Response to Reviewers

Rare but Resilient: Dispersal diversity buffers species vulnerability

Manuscript NATECOLEVOL-25082966-T

D. Bernardi, G. Nicoletti, P. Padmanabha, S. Suweis, S. Azaele, S. Levin, A. Rinaldo, A. Maritan

The Authors would like to thank the Editor and the Reviewers for their careful evaluation of our manuscript, in particular for their comments, which we found insightful and constructive. We have addressed all comments in detail below. In response, we significantly revised the manuscript to clarify the details of the model and its biological interpretation, and to broaden its connection to the existing literature. We also carried out extensive new analyses, including formal model comparisons, robustness checks, and the addition of a temperate forest dataset (the Michigan Big Woods forest plot), to address the methodological concerns raised by the reviewers.

These revisions and the expanded analyses strengthen the manuscript while supporting its earlier main conclusions. We provide both a marked-up version of the manuscript, in which changes are highlighted, and a clean version. Owing to the scope of the additional analyses, the Supplementary Information has been substantially expanded and should be regarded as a fully revised document.

We trust that our revisions have significantly improved the manuscript and hope that it is now suitable for publication.

Response to Reviewer 1

Reviewer:
This model introduces new theoretical models for species dispersal and competition, alongside novel ways to quantify the success of these models. The approach is tested at two tropical forest plots, and fits to data for both the novel distributions, and also the prediction of whether all species would be predicted to coexist, are impressive.

There is something new and important here that is working well, but my two major comments are as follows, and both relate to a deeper understanding of what the model is getting right (and doing better than alternative models), as well as a clearer understanding what type of data is being used to parameterize the species-specific distributions.

We are grateful to this reviewer for their appreciation of our work. Their comments and suggestions have been instrumental in our revision, helping us to significantly strengthen our results and conclusions, as we detail below.

Reviewer:
(1) To my knowledge the VAAD (the vacancy-adjusted abundance distribution) is introduced here—or if it is in other papers by the authors my understanding is that this must be fairly recently introduced. The success of the model is tested in part by the matches between predicted and observed VAADs, and these are impressive. However, it took me a while to grasp both what this distribution was, and also its significance (and I am still not sure of the latter).

In short, the VAAD depends critically on how empty a local patch is relative to how many individuals it COULD support. Thus even if in two patches a given species has the same relative abundance, if one patch is half full, while the other is maxed out, these two will contribute differently to the VAAD for that species. This differs from most approaches that would be based on relative abundance, independent of how full or empty a patch is. So this is an important difference, that I think should really be brought front and center, and not relegated to one among many quantities defined in a box. The authors perhaps should put a clearer explanation in the main text when it is first mentioned.

We thank this reviewer for the insightful comments. The vacancy-adjusted abundance (VAA) is indeed introduced in this work for the first time, and we agree that both its definition and ecological significance were not sufficiently emphasized in the original manuscript.

Following this suggestion, we have revised the main text to provide a clearer and more intuitive explanation of the VAA concept at its first introduction. We now dedicate significantly more space to it. In particular, we

now explicitly mention that VAA differs from standard relative abundance by normalizing species occupancy by the locally available free space, so that identical abundances in patches with different saturation levels contribute differently. The revised text reads:

> Main text, l. 189
>
> The link between model and data is provided by a metric that emerges from the analytical solution of the model using generalized dynamical mean field theory (DMFT) [42]: the *vacancy-adjusted abundance* (VAA). The VAA of species α at a given site is defined as the fraction of space it occupies divided by the locally available (unoccupied) space. This normalization accounts for local space saturation, making the VAA dependent on how empty a local site is relative to its capacity. Therefore, a species with the same relative space-use in different subplots contributes differently to the VAA depending on how much each patch is occupied: a subplot with very low free space amplifies the VAA for species occupying this spot, and it reduces it in subplots that have larger free space. Although the VAA arises naturally from the DMFT calculation (Methods), it admits a clear ecological interpretation: by upweighting space-limited patches, it rescales local relative space occupation to reflect the effective colonization effort.

To further emphasize the VAA's central role, we have also revised Figure 1 to highlight the VAA as the key observable in linking spatial data to the inferred model parameters and subsequent analysis. This conceptual link is now explicitly described in the accompanying text:

> Main text, l. 200
>
> For each species, the parameters of the VAA distribution quantify its dispersal heterogeneity (δ_α^{-1} in table 1) and an effective dispersal strength ($\bar{\beta}_\alpha^{-1}$ in table 1), which depends on the surrounding community. Fitting empirical VAA distributions therefore enables inference of species-specific dispersal strategies without requiring direct measurements of dispersal or interaction parameters. Because the parameters of the theoretical VAAD are linked to those of the interaction-dispersal kernel through an implicit integral equation, fitting the theory to data enables inference of this kernel, which encodes species-specific spatial strategies. In this way, the VAA distribution provides a direct link between observable space-use patterns and the underlying interaction-colonization structure, while remaining a model-independent quantity that can be estimated from spatial abundance data. The overall conceptual pipeline is illustrated in Fig. 1.

Reviewer:
That being said, I am still trying to get my head around what this vacancy-adjustment means and how it should be intepreted. There is a lot of interesting recent work at BCI and in other forests on gap dynamics. Some of this is referenced here, but perhaps more literature could be added. These gap dynamics highlight the differences in the way species with different life history strategies (e.g. pioneers vs shade tolerant) respond to gaps—specifically that pioneers will grow quickly to fill gaps. So... the vacancy-adjustment seems to have a life history implication, such that some types of species (in terms of their life history strategies) may have quite different vacancy-adjusted abundances vs non-vacancy adjusted, while for others these may be much more similar. But this was not quite spelled out and so I was not sure how to go further than that.

So there is clearly something interesting there, and I am asking the authors to spell that out, and put it in context of other work related to gaps, life history variation across tropical trees, and ways to quantify each of these.

We thank this reviewer for the insightful perspective put forth, which prompted us to make the connection between the VAA and the species' life-history strategies more explicit. In our VAA framework, canopy gaps – i.e., patches endowed with a large amount of unoccupied space – amplify the contribution of species that successfully colonize them. This leads to a direct link between VAA heterogeneity and the evolution of ecological strategies. Species that rapidly exploit gaps (e.g., pioneers) tend to produce highly heterogeneous, right-skewed VAA distributions, while shade-tolerant species that colonize more uniformly produce more homogeneous VAA patterns.

To test this interpretation, we used the guild classification for BCI species proposed by Comita et al. (2007), which groups species into shade-tolerant, gap-exploiting, and intermediate categories. We find that the inferred VAA parameters support this picture. In particular, gap specialists have significantly larger δ_α^{-1}, consistent with more heterogeneous spatial distributions.

This result is now presented in Fig. 2, and a detailed statistical analysis is provided in the Supplementary Information (Section 3). We now write:

> Main text, l. 260
>
> Crucially, besides achieving a better fit to the VAA distributions, our model also yields two species-specific parameters that carry clear ecological information: dispersal heterogeneity and effective dispersal strength (table 1). Based on the meaning of the VAA, we hypothesize that VAA heterogeneity also reflects differences in life-history strategies. To test this, we compared parameter values across functional guilds in the BCI dataset (shade-tolerant,

> gap-exploiting, and intermediate; classification from ref. [49]), focusing on a grouped comparison of gap-exploiting and intermediate species versus shade-tolerant species. We found no statistically significant difference between guilds in the effective dispersal strength (Fig. 2E; see caption for details on the statistical test). In contrast, gap-exploiting and intermediate species exhibit significantly larger dispersal heterogeneity than shade-tolerant species (Fig. 2F). This difference between gap exploiters and shade-tolerant species remains significant when treating all three guilds separately (SI, Sec. 4). These results are consistent with the role of canopy gap dynamics in promoting episodic local dominance of light-demanding species [50, 51], and with recent work highlighting the role of life-history strategies in shaping species responses to environmental variability and enabling coexistence [17, 52].

Reviewer:
That being said, I had two major question marks here, both relating to the definition of "vacancy". Once we are thinking about life history differences, and the speed at which a species will fill in gaps, etc, we are inevitably talking about stage or size-structured populations. But then, that means that not all abundances in a sublot are comparable. Some subplots may have many small, young trees, while others may have fewer, odler, larger trees. But they may have the same amount of space actually physically "vacant". So my question here is whether abundance is even the correct currency to capture what the authors are trying to do, or whether they may want to take into account weighting this with basal area or something similar.

This is an important and well-taken point. The reviewer is correct that treating all individuals equally, regardless of size, is a simplification, as individuals differ substantially in the amount of physical space they occupy.

To address this comment, we introduced a DBH-weighted metric accounting for the individual sizes by exploiting the allometric scaling relation between trunk diameter and crown area, which has drawn compelling theoretical and empirical support (Simini et al. *PNAS* 2010; Martinez-Cano et al. *Biogeosciences* 2019).

We have added the following clarification in the main text where the VAA results are presented

> Main, l. 219
>
> We computed the VAA empirical distributions from data, as described in Methods. Briefly, we partition each plot into square subplots of side length $L = 50$ m, a spatial aggregation scale that balances sufficient sampling within subplots with the need to retain spatial resolution (Supplementary Information, sec. 1, for details of optimal scale selection); we then estimate the space used by each species in each subplot by accounting for each tree's size, following the established allometric scaling relation between trunk and crown size [37, 38]; next, we infer the per-subplot effective total space capacity using a global fitting procedure. Finally, we construct the empirical VAA histogram as per its definition (see table 1).

and then in the methods:

> Methods, l. 1058
>
> For a given spatial scale L, we estimate the effective space within patch i occupied by species α, denoted $a_{\alpha i}$, using two alternative metrics. The simpler *stem-count* metric sets $a_{\alpha i} = n_{\alpha i}$, the number of individuals, implicitly assuming equal space occupancy. The *DBH-weighted* metric instead defines $a_{\alpha i} = \sum_j d_{\alpha i,j}^{4/3}$, where $d_{\alpha i,j}$ is the diameter at breast height (DBH, in cm) of the j-th individual of species α in subplot i. The exponent 4/3 follows from the allometric scaling between trunk diameter and projected crown area, which is well established both theoretically and empirically [37, 38], so that $a_{\alpha i}$ approximates the effective canopy space occupied by species α. We verified that both metrics yield consistent estimates of the effective per-patch carrying capacity (see below). Based on a quantitative goodness-of-fit comparison across spatial scales and metrics (SI, Sec. 1.1), we adopt the DBH-weighted metric as the primary abundance measure and $L = 50$ m as the standard spatial scale for all main-text analyses. For model comparison, however, we use the stem-count metric. Incorporating DBH-weighting in the null models would require additional assumptions on the sampling of individual sizes and introduce further arbitrariness, while also increasing the number of parameters. Using stem counts therefore ensures a consistent and fair comparison across models.

We then systematically compared the original count-based metric and the DBH-weighted metric across all datasets, species, and spatial scales. We find that the DBH-weighted metric consistently improves fit quality, as quantified by both Cramér–von Mises and Kolmogorov–Smirnov statistics. Based on this analysis, we adopted the DBH-weighted metric as the primary measure throughout the revised manuscript, and updated all main-text figures accordingly. The full comparison of metrics and spatial scales is reported in the Supplementary Information (Section 1).

Reviewer:
Related, but slightly different, as mentioned above the vacancies are defined relative to the maximum number of individuals observed in a subplot. There is then the issue above wrt how big are the individuals in different subplots. But there is another issue. Forest Dynamics plots are chosen to be relatively homogeneous, but there could still be fundamental differences in productivity between different subplots due to soil, topography,

or some other unknown factor. So this makes me question in a different way just how easily we can know the true amount of vacancies in a given subplot, which is criticlaly important for defining the VAAD.

We agree with this reviewer that the maximum capacity of a patch is an important (and not directly observable) parameter of our framework, which may, in principle, vary due to environmental heterogeneity. In our framework the effective carrying capacity, M, represents the total space available within a subplot. It is not fixed a priori, but rather treated as a parameter, and as such, inferred directly from the data as part of the calibration process. We acknowledge that this aspect was not presented in a sufficiently clear manner in the original manuscript. We have clarified this point in the main text as:

> MAIN, L. 224
>
> [...] Next, we infer the per-subplot effective total available space using a global fitting procedure (see Methods for details). [...]

and in more detail in the Methods

> METHODS, L. 1079
>
> We define the per-patch space use as $p_{\alpha i} = a_{\alpha i}/M$, where M is a free parameter representing the total carrying capacity of each subplot, assumed to be spatially homogeneous (we investigated the robustness of this assumption in the SI, sec.4). To determine M, we scan a coarse grid of candidate values, starting from $M_0 = \max_i \{\sum_\beta a_{\beta i}\} + 1$, the minimum value ensuring a positive denominator in the VAA, and incrementing upward. We then refine the search around the best coarse candidate using a finer grid. For each candidate M, we compute the VAA for all species and patches using
>
> $$x_{\alpha i} = \frac{p_{\alpha i}}{1 - \sum_\beta p_{\beta i}}, \quad (1)$$
>
> and fit, independently for each species, the empirical cumulative distribution of non-zero VAA values to the theoretical Gamma distribution, eq. (13), via least squares. The optimal M is selected by minimizing the total CDF mean-squared error across all selected species. This procedure is applied for both the stem-count and DBH-weighted metrics.

As mentioned in the quoted excerpt, we performed additional analyses reported in the Supplemental Material (sec. 4). We find that the inferred M scales linearly with patch area ($M \propto L^2$), as expected for a space-based quantity, and that the corresponding density M/L^2 is approximately constant across spatial scales within each dataset, while differing between forest types in a biologically consistent manner. To address the reviewer's concern about patch-to-patch heterogeneity, we performed an additional analysis in which each patch j is assigned its own effective capacity M_j, inferred through a regularized optimization procedure that penalizes deviations from a global value. We find that allowing for heterogeneous M_j leads to only minor improvements in goodness-of-fit, indicating that a single effective capacity M provides an adequate coarse-grained description of the data. These results support the interpretation of M as an effective, spatially averaged quantity that captures the dominant constraints on local occupancy, while finer-scale environmental variability is absorbed into the inferred statistical variability of the interaction kernel.

Reviewer:
(2) The VAAD fits were impressive, but there were also several other quantities that seemed to match the authors' model very well. This is a species-specific model, so in principle there are a large number of parameters, unlike (for example) a neutral model. As far as I can tell, many of these parameters are fixed by the data, though what was unclear to me was exactly what aspects of the data are being used to parametrize the models, and how independent is this data used for fitting the models from the data being used to test the model outcomes. Relatedly, are any model parameters left as truly free parameters, that are then tuned to fit the data? Both of these points could be made clearer.

We thank this reviewer for pointing out the two related concerns. We address them separately.

(i) All parameters are fitted from data. We clarify that all model parameters are inferred from the spatial abundance data within the model framework, without the use of external biological measurements or ad hoc tuning parameters. Specifically, for each species, the parameters δ_α and $\bar{\beta}_\alpha$ are obtained by fitting the analytical VAA distribution to the empirical VAA histogram. The global carrying capacity M is then determined self-consistently within the same framework.

We acknowledge that this inference pathway was not sufficiently explicit in the original manuscript. Thus, we have clarified it: in the revised manuscript, we mention this briefly in the main text when we introduce the kernel

> MAIN, L. 162
>
> [...] The parameters of this distribution are species-specific, meaning that each species has its own set of

> parameters, and quantitatively capture diverse colonization strategies. These parameters are not prescribed a priori, but inferred directly from observed spatial abundance data. [...]

and in more detail in the Methods section

> METHODS, L. 825
>
> The kernel matrix $\mathbf{K}_\alpha$ is an *effective* species-specific interaction–colonization kernel. It quantifies both how species interact and how they disperse between patches. The kernel is not measured directly from field data, nor do we attempt to reconstruct each individual kernel entry $K_{\alpha,ij}$ separately. Doing so would require detailed knowledge of local demographic rates, dispersal between all pairs of patches, and interactions in every local environment, which is generally infeasible in species-rich and spatially heterogeneous systems. Instead, we assume that, for each species, the entries of $\mathbf{K}_\alpha$ are drawn from a statistical distribution $\mathcal{Q}_N^{(\alpha)}$ characterized by a small number of species-specific parameters. These parameters capture the variability in interactions and dispersal strategies among species. Although the kernel elements are random, we are able to infer the parameters of $\mathcal{Q}_N^{(\alpha)}$ indirectly from spatial abundance patterns for each species, by exploiting an explicit solution of the model.

We then describe the actual fitting procedure explicitly in the Methods:

> METHODS, L. 1094
>
> **Species-specific dispersal parameters**
>
> After determining the optimal value of M, we fitted the VAA distributions of each species at all levels of coarse-graining using Eq. 13. This allowed us to obtain the values of δ_α and $\bar{\beta}_\alpha$. A detailed GOF analysis for all > 500 species at all spatial scales is presented in the Supplemental Material, together with visualization for 212 species ($100 + 100 + 12$ from the three datasets).

(ii) Independence of fitting and evaluation data: spatial cross-validation. To address the concern about the independence of fitting and evaluation data, we performed a spatial cross-validation analysis. For each dataset, we generated 20 random 50/50 splits of the set of patches, fitted the model parameters on one subset, and evaluated the predicted mean abundances on the held-out subset. The results presented in the revised Fig. 2B are explicitly presented as a train-test analysis.

> MAIN, L. 238
>
> Then, we investigated whether our model generalizes to unseen data. Specifically, we used the parameters inferred on half of the forest plot (with different random partitions) to predict the mean fraction of space occupied by each species in the remaining half. This out-of-sample validation of our theory resulted in a remarkable agreement for all three datasets (Fig. 2B), showing that our analytical predictions are well-aligned with empirical observations.

The out-of-sample validation is explained in more detail in the new Methods subsection "Out-of-sample predictive accuracy" at l. 1108 and in the new Sec. 2 of the Supplemental Information, where the train-test analysis is extended to the fit parameters, showing that the model predictions generalize beyond the specific data used for fitting also in this respect.

We have also addressed the point about the number of parameters below, specifically in our response to point (4), the model comparison.

> ***Reviewer:***
> *(3) It may not have been the goal of the authors, but I wonder if they have used their model to make predictions for temporal fluctuations, or other kinds of temporal predictions.*

Good point. We agree that extending the framework to make temporal predictions, such as changes in species abundances among censuses, would be highly valuable. However, this would require going beyond the quasi-stationary approximation, for instance by incorporating finite-time dynamics or fluctuations around the spatial fixed point. Both directions are scientifically sound, and we think they are important for the community. However, they represent a substantial extension of the present theory and are outside the scope of this study. We have added a remark on this matter as an outlook in the Discussion:

> DISCUSSION, L. 553
>
> [...] Understanding the factors that drive these variations in transient behavior is an important direction for further investigation [65, 66].
> Closely related to this, at very low abundances, demographic fluctuations are expected to become increasingly important. Recent work has highlighted the need to introduce probabilistic frameworks into coexistence theory [10]. In this context, important directions for future work include incorporating stochastic effects and accounting for temporal fluctuations [17, 52] to move beyond deterministic proximity to exclusion, and exploring whether these can be quantitatively linked to the spatial buffering effect described here.
> A further limitation [...]

Reviewer:
(4) Finally, to put the results in context, I would like to see a comparison with alternative models that could potentially predict the same quantities. For example, this might be a neutral model, or a model with species-specific stage-structure or gap dynamics, or something else that is plausibly related to the authors' model. Seeing how much better this model performs, and also (imprortantly) comparing the data overhead of exactly how much data is needed to parametrize those alternative models versus this one, would help put the results in context.

We thank this reviewer for the important suggestion. To place our results in context, we have performed an extensive comparison with alternative models, focusing on a hierarchy of models that serve as standard neutral (or else non-neutral) baselines for spatial abundance data. Specifically, we have fitted three Negative Binomial (NegBin) variants: (i) a fully neutral model (3 parameters), (ii) a shared-dispersion model ($S + 2$ parameters, with S equal to the number of species), and (iii) a per-species NegBin with the same number of parameters as our model ($2S + 1$). This allows us to present a direct comparison among models with matched flexibility.

Notably, across all datasets and spatial scales, our model consistently outperforms all NegBin variants. The fully neutral model performs substantially worse, confirming that species-specific structure is essential. It is also relevant that even the per-species NegBin – despite having the same number of parameters – fails to match the fit quality of our model. This suggests that the improvement is not due simply to increased flexibility, but rather reflects a more appropriate functional form.

A comprehensive account of the results, including details about the statistical methods used to quantify each model's performance, is shown in the supplemental information (sec. 6). A concise summary of these new results is presented in the revised main text (figs.2C and 2D). For brevity, in the main text, we focus on the two extreme variants of the null model, the fully neutral or the fully species-specific. The accompanying text reads as follows:

> MAIN, L. 243
>
> As a final check, we compared our framework against ecological null models in which species are independent and overdispersed, leading naturally to a Negative Binomial description of abundances [47]. We considered two variants (Methods): a neutral model parameterized at the community level, closely related to neutral theories of biodiversity that reproduce several large-scale ecological patterns in species-rich systems [48], and a species-specific model fitted independently for each species, matching the total number of parameters of our framework. We evaluated model performance by fitting the VAA distributions of all species across the three datasets (see Methods). For each dataset, the absolute goodness-of-fit of our model is consistent with the reference level expected for a correctly specified model (Fig. 2C, dashed line). In contrast, the species-specific model provides an adequate fit only for the Michigan dataset, while the neutral model exhibits substantially larger errors across all datasets, suggesting model misspecification. In a relative goodness-of-fit comparison, our framework consistently outperforms the neutral model across all datasets (positive KS log-ratio in Fig. 2D). Relative to the species-specific model, it yields a marked improvement in Pasoh and Michigan, and a comparable, slightly better performance in BCI. Full details are provided in the SI (Sec. 6), where an intermediate variant with partially shared parameters is also analyzed.

To address the reviewer's question regarding data requirements, we performed a subsampling analysis in which we refitted all models using increasing fractions of the available patches. We find that our model achieves statistically consistent fits (as quantified by the Cramér–von Mises criterion) once a sufficient number of patches is available, while the NegBin models plateau at suboptimal fit quality regardless of sample size. This indicates that the improved performance of our model reflects reduced model misspecification rather than increased data demands.

These results are presented in full in the Supplementary Information (Section 7). Here, we also have a dedicated subsection where give an estimate of approximate practical data requirements necessary for a reliable parametrization of the model to facilitate future applications of the framework. We have added also this remark in the main text briefly summarizing this point and referring to the SI:

> MAIN, L. 257
>
> [...] Additional analyses of data requirements (SI, Sec. 7) show that reliable parameter estimation is achievable even for smaller datasets and provide lower-bound estimates on data requirements.

Response to Reviewer 2

Reviewer:
Overall, I really enjoyed this paper and think it is a cool model. There are a couple of major issues, however, that I feel need to be resolved, but I think all, except possibly one (facilitation) can be dealt with in revision of the text.

We thank this reviewer for their positive assessment of our work. We are grateful for the detailed, line-

by-line comments, which have been instrumental in our revision. A point-by-point response follows below.

> ***Reviewer:***
> *1. Overall, I do not think you have sufficiently or accurately placed your work in the context of the relevant modern literature. You set up the premise of this paper by citing classic papers, ignoring the large body of literature that has been trying to add biological realism to both coexistence and spatial process modelling in recent years. This needs to be resolved. These are the concepts that you suggest you are the first to think about with this paper, that have definitely been examined, if using different approaches in the literature: coexistence as a probability or feasibility domain, spatially explicit coexistence, spatially explicit extinction risk, spatially explicit models of community diversity and abundance patterns that account for species traits and vital rates. What is new in this paper is your approach. I know you have a limit on references, but I think you can easily get rid of some of the less relevant in lieu of the more relevant ones I suggest here or that you find upon more careful research of the recent literature. Please have a look at these references and revise your introduction to make it more accurate about you adding to a growing literature seeking to add biological realism to models of community diversity and extinction probabilities. a. D. Garcıa-Callejas, I. Bartomeus, and O. Godoy. The spatial configuration of biotic interactions shapes coexistence-area relationships in an annual plant community. Nature Communications 12.1 (2021), p. 6192. b. Ellner, S. P., Snyder, R. E., Adler, P. B., & Hooker, G. (2019). An expanded modern coexistence theory for empirical applications. Ecology letters, 22(1), 3-18 c. Grilli, J., Adorisio, M., Suweis, S., Barabás, G., Banavar, J. R., Allesina, S., & Maritan, A. (2017). Feasibility and coexistence of large ecological communities. Nature communications, 8(1), 14389. d. Grainger, T. N., & Gilbert, B. (2016). Dispersal and diversity in experimental metacommunities: linking theory and practice. Oikos, 125(9), 1213-1223. (This one is particularly relevant as background for your study)*
>
> *From the spatial perspective you have also over-simplified the existing literature – there is a lot of effort to add biological realism in joint-species distribution models (For example: Harris, D. J. (2015). Generating realistic assemblages with a joint species distribution model. Methods in Ecology and Evolution, 6(4), 465. There are lots others) e. Keil, P., Wiegand, T., Tóth, A. B., McGlinn, D. J., & Chase, J. M. (2021 . Measurement and analysis of interspecific spatial associations as a facet of biodiversity. Ecological Monographs, 91(3), e01452. f. Wiegand, T., Grabarnik, P., & Stoyan, D. (2016). Envelope tests for spatial point patterns with and without simulation. Ecosphere, 7(6), e01365.*

We thank this reviewer for the useful suggestions. We came to agree that the original manuscript did not properly acknowledge relevant contributions, especially from recent literature. We have thus substantially revised the introduction to better reflect this broader context as suggested by this Reviewer. We now explicitly refer to recent advances in coexistence theory, spatially explicit modeling, and joint species distribution approaches, including all references suggested by the reviewer. The revised introduction now ensures a more balanced positioning of our work within the current ecological literature:

> Introduction, l. 60
>
> [. . .] Ecological theories have made substantial progress on different aspects of this problem. Coexistence theory details broad mechanisms by which species persist together [5, 6, 7, 8], with recent work extending these ideas to empirical measurement [9], demographic uncertainty [10], and community-level characterizations of the feasibility regions [11, 12]. Metapopulation [13] and metacommunity [14] frameworks have shown how spatial structure and dispersal shape diversity [15], while broader theoretical syntheses have connected biodiversity to ecosystem stability [16], life-history variation [17], and macroecological patterns [18]. Yet, these advances have largely proceeded in parallel: analytical tractability for species-rich communities has been achieved mainly in non-spatial settings [19, 11], while spatially explicit models remain difficult to scale to many interacting species.
>
> Spatial heterogeneity shapes coexistence in multiple ways: through stabilizing mechanisms such as the storage effect and dispersal limitation [20, 21], by giving rise to interspecific spatial associations [22], and by modulating coexistence–area relationships through the configuration of species interactions [23]. Empirical tools for quantifying these patterns include spatial point pattern analysis [24] and joint species distribution models [25]. Recent work has begun to integrate spatial patterns with interaction-based descriptions of communities [26, 27, 28]. Yet, integrating spatial structure into mechanistic models of interacting communities remains challenging. Analytical results typically become intractable, while numerical simulations depend sensitively on model assumptions and parameterization, and offer limited scalability [29].
>
> On the empirical side, data-driven methods (such as species distribution models [30], occupancy scaling methods [31, 32], Population Viability Analysis [33], or Matrix Population Models [34]) combine statistical inference with some mechanistic elements, but often focus on single-species dynamics and typically require detailed demographic and environmental data [35, 36]. Hence, there is a need for frameworks that combine spatial structure with multispecies interactions in a way that is analytically tractable and compatible with the coarse-grained data available for most species.
>
> Here, we . . .

Reviewer:
2. One of the big changes in how coexistence is being modelled these days is a shift away from overly simplistic direct competition models to those that allow for complex multispecies interactions and facilitation. As far as I can tell you have done neigther of these things. This is a really cool model but to me the exclusion of facilitation is a problem, and one that does possibly impact your results. In particular, I would like to know if the inclusion of facilitation would improve your model fit at the smallest spatial scales. These are the scales at which I would expect to see strong facilitation so it makes sense these are the scales at which your model fits least well. I can't tell from your methods if you have omitted facilitation for mathematical reasons) – most Lotka-Volterra based models break if you all facilitation, or if it is because you decided it doesn't matter but you need to try. Facilitation is extremely common in tropical forests, so I don't accept the argument that this is a minor and uncommon process and thus can be ignored, especially as there is a pattern that seems like it could result from that omission. As I comment on below, I really cannot tell how you incorporated competition into your model but the exponential plant population growth model in (M. Mayfield and D. Stouffer, 2017. 2017 Higher-order interactions capture unexplained complexity in diverse communities. Nature Ecology and Evolution 1(3):006 DOI: 10.1038/s41559-016-0062) is one way to do this, though it may not be relevant for your model – I can't tell. at very least, explain how its inclusion is likely to change your results. Here are some relevant papers on facilitation and why it is so important to include.
1. G. Losapio et al. "Network motifs involving both competition and facilitation predict diversity in alpine plant communities. In: Proceedings of the National Academy of Sciences 118.6 (2021), e2005759118. 2. B. Pichon et al. The interplay of facilitation drives the emergence of multistability in dryland plant communities. In: Ecology (2024), e4369 3. E. J. McIntire and A. Fajard . Facilitation as a ubiquitous driver of biodiversity. New phytologist 201.2 (2014), pp. 403–416. 4. L. Buche et al. A continuum from positive to negative interactions drives plant species' performance in a diverse community. Ecology Letters 28.1 (2025), e70059. 5. M. Bimler, M. Mayfield, and A. James, 2025. Facilitation thinking for coexistence theory. Ecology Letters 28 (6), e70150.

We thank this reviewer for raising an important point, and for highlighting the role of facilitation in ecological communities. We agree that facilitative interactions can be a key driver of coexistence and that it is important to address their relevance in the context of our work.

In the revised version of the manuscript, we have clarified that, in our framework, the interaction–dispersal kernel should be interpreted as an effective, coarse-grained representation of local ecological processes. Since we infer the parameters of the kernel directly from data, through the fits of the vacancy-adjusted abundance (VAA) distributions, we are not able to disentangle the different contributions to the effective interactions represented by the kernel. In particular, it does not explicitly separate competitive and facilitative interactions, but instead captures their net effect on space occupation after averaging over local environmental variability and species co-occurrence. Hence, the references to "competition for space" should be understood in terms of the shared constraint of finite space, which cannot be simultaneously occupied by multiple individuals. Thus, the effective interactions in our model – inferred from data – do not necessarily account for competition alone. We have realized that the previous version of the manuscript did not make this point clear. This interpretation is now further clarified and strengthened in the revised manuscript. The revised text now reads:

> MAIN, L. 106
>
> We study a general metacommunity model describing the temporal dynamics of species abundances in a habitat composed of N colonizable sites, each providing a finite amount of available space or a limiting resource. These sites may correspond to distinct patches, although our framework does not strictly require explicit physical boundaries. The model incorporates basic processes such as death, reproduction, dispersal, colonization, and competition for space. Their combined effect is captured by an interaction-dispersal kernel matrix, $\boldsymbol{K}$, whose elements represent a coarse-grained description of how species interact and spread across patches. However, they do not explicitly separate local competitive and facilitative interactions, but capture instead their net effect on dispersal efficacy.

> TABLE 1, ENTRY ABOUT THE INTERACTION-DISPERSAL KERNEL
>
> **Interaction-dispersal kernel.** Species-specific matrix describing interaction and dispersal processes of species α. The kernel matrix $\mathbf{K}_\alpha$ bundles into a single quantity local birth and growth rates, colonization-mediated dispersal, and local interactions of species α with other species, such as competition and facilitation (in the form of effective competitive suppression). Its spatial distribution is assumed to be a species-specific Gamma distribution. It is not directly measurable, but its parameters are inferred from the data by fitting the analytical theory for the VAA to the empirical VAA distribution.

Furthermore, following a suggestion by Reviewer 1, in the revised manuscript we quantify the fraction of space occupied by each species in each subplot accounting for the size of each individual tree, using DBH-weighted abundances. This provides a proxy for the actual physical space occupied by individuals, strengthening the interpretation that our metrics capture the net outcome of both competitive and facilitative processes

implicitly, as a local increment or reduction of the space that is available for colonization.

Let us remark that we believe that this coarse-grained description is a strength of the approach, as it allows us to infer species-specific effective interaction parameters directly from spatial data. At this level of description, however, this unfortunately prevents us from disentangling distinct mechanisms of local competition and facilitation. Nevertheless, since we realized the importance of this point, we tested whether we could improve the model's performance by incorporating an explicit facilitation term via a tractable community-level proxy: a per-species parameter $\Omega_\alpha \in [0, 1]$ that rescales the effective available space. In practice, Ω_α captures facilitation as a further reduction of competition (besides the one already implicitly encoded in the kernel). A non-zero Ω_α would indicate a net facilitative effect that, crucially, is not already captured by the inferred kernel. That is, a value of Ω_α close to zero indicates that some form of facilitation is already included in the effective parameters that characterize the kernel and that are inferred from data. We estimated Ω_α using a regularized optimization procedure and found $\Omega_\alpha \approx 0$ across species and datasets. The resulting improvements in goodness-of-fit are small, indicating that the baseline model already captures the dominant interaction patterns, including facilitation, present in the data. These results are presented in detail in a new dedicated section of the Supplementary Information (Sec. 5), and succintly mentioned in the main text

> MAIN TEXT, L. 227
>
> Fig. 2A compares the theoretical VAA predictions, fitted to the empirical distributions, for three representative species per dataset. We observe excellent agreement, with the model accurately capturing the diversity of distributional shapes across species. These differences are naturally accounted for by species-specific dispersal strategies (Methods). Goodness-of-fit analyses for all analyzed species, along with explicit visualizations for 212 species, are reported in the Supplementary Information (SI).
>
> Next, we performed a series of model robustness checks. First, because the net effect of space limitation is competitive, we tested whether explicitly incorporating facilitation [46, 8] – beyond the competition buffering implicitly encoded in the inferred parameters of $\mathbf{K}$ – could improve model performance. This extension resulted in only marginal changes in model fit across the three forest datasets (SI, sec. 5). This suggests that, at the spatial and temporal scales considered here, the dominant effects of facilitation may already be effectively captured by the inferred kernel parameters.

Regarding the deviations observed at small spatial scales, we tested whether the inclusion of this facilitation proxy improved model performance at small scales, but found no substantial improvement. Instead, these deviations are consistent with known limitations of the dynamical mean-field approximation: at small L, patches contain few individuals, violating the "typical-patch" assumption, and discreteness leads to many zero-count patches for rare species. These effects diminish at larger spatial scales, where the model provides robust fits. Indeed, the additional analysis of spatial-scale dependence reported in sec. 1.1 of the supplemental material led us to select $L = 50$ m as the optimal scale. This choice is supported by quantitative goodness-of-fit criteria, which confirm that the model loses accuracy at very small spatial scales.

Finally, in the Discussion, we highlight that modeling explicit local facilitation is indeed an important future direction and outline how it could be incorporated in future extensions, acknowledging the literature on facilitation suggested by the reviewer. We write

> DISCUSSION, L. 561
>
> A further limitation is that distinct mechanisms of local competition and facilitation cannot be disentangled in our framework. At the level of description provided by our model, both contribute by amplifying or buffering the inferred kernel parameters. As recent works have highlighted the fundamental role of facilitation in driving coexistence [46, 67, 56, 8], extending the framework to explicitly resolve facilitative interactions either through pairwise or trait-mediated effects [68, 69] remains an important direction for future work. This extension is particularly relevant in systems where such interactions are expected to play a stronger or more structured role, and would likely require going beyond the current analytically tractable formulation of the model.

Reviewer:
3. I think your model is very interesting, but the idea that trait ratios are important for determining the fate of species in communities is not new – again, I want to see a discussion (brief) of how your model adds to and moves beyond these past studies in this research area that have also used trait differences to explain communities. 1. D. Tilman. The resource-ratio hypothesis of plant succession. The American Naturalist (1985), pp. 827–852 2. M. Mayfield and J. Levine. 2010 Opposing effects of competitive exclusion on the phylogenetic structure of communities. Ecology Letters. 13:1085-1093

We thank this reviewer for highlighting the connection to trait-based and resource-ratio approaches, which indeed have a long and important history in coexistence theory. Our framework is conceptually related to these approaches, but differs in several key respects: First, our competitive balance is not constructed from independently measured biological traits (such as resource uptake rates or demographic parameters), but is

inferred directly from spatial abundance patterns via the analytical solution of the spatial model. This makes the approach applicable to species-rich communities where comprehensive trait data are often unavailable. Second, spatial structure and interspecific interactions are intrinsically coupled in our framework. Because species compete for a shared, spatially distributed resource (space), the competitive balance of a given species depends on the full community composition, rather than being defined in isolation. Third, our framework goes beyond predicting coexistence outcomes by providing a quantitative measure of proximity to exclusion (vulnerability), allowing us to assess how close each species is to its exclusion boundary.

We have clarified the above connections and made relevant distinctions explicit in the revised Discussion:

> Discussion, l. 518
>
> The idea that trait-derived quantities govern competitive outcomes has a long theoretical history, from resource-ratio theory [54] to modern trait-based coexistence frameworks [55, 56]. Our approach extends this lineage by inferring the relevant trade-off directly from spatial abundance data, without requiring prior trait measurements. In this sense, our measure of competitive balance can be viewed as a community-wide generalization of the competition-colonization trade-off [57, 58, 59, 40, 60]. Although community-level manifestations of this trade-off have been reported [61, 62, 21], their implications for coexistence in heterogeneous landscapes have remained unclear [63]. Here, we make this link explicit and show how such trade-offs shape species' risk of competitive exclusion. Frameworks that characterize coexistence as a feasibility domain in parameter space have enabled empirical applications, but rely on estimates of interaction coefficients or trait-based proxies [12]. In species-rich systems, such information is typically unavailable, and incomplete knowledge of niche dimensions can substantially affect the inference of interaction structure [64]. By contrast, the analytical solution of our spatial model integrates dispersal and interaction effects at the community level, requiring only spatial census data for its application. Our vulnerability index therefore provides a flexible, context-dependent measure that accounts for the community composition. That is, a species with low vulnerability in one metacommunity may be at risk in another, depending on both species composition and spatial structure.

Reviewer:
4. Your general modelling approach seems to align with other recent models of coexistence that look at coexistence as a probability distribution or as a feasibility domain. Please address in your paper how your model fits within this approach to coexistence modelling – advances it or just increases its usefulness, acknowledge it all the same. I think yours is different but you need to acknowledge that you are not the first group to think about coexistence this way. 1. J. W. Spaak and F. De Laender. Intuitive and broadly applicable definitions of niche and fitness differences. Ecology Letters 23.7 (2020), pp. 1117–1128. 2. C. Bowler, C. Weiss-Lehman, I. Towers, M. Mayfield, and L G Shoemaker 2022. Determining the probability of coexistence: Adding demographic variation to modern coexistence modelling. Ecology Letters 25(7): 1618-1628

We thank this reviewer for pointing out the connection to feasibility-based and probabilistic approaches to coexistence. Our framework is related in spirit, in that it moves beyond a binary notion of coexistence and instead quantifies how close each species is to exclusion. In feasibility-based approaches, coexistence is typically characterized in terms of a feasible region in parameter space, while probabilistic extensions incorporate demographic variability and uncertainty.

We have now clarified the above connections in the revised manuscript. These approaches are first credited in the introduction (see excerpt quoted in response to point 1 above). Then, the connections to our work are better discussed in the revised discussion:

> Discussion, l. 525
>
> Frameworks that characterize coexistence as a feasibility domain in parameter space have enabled empirical applications, but rely on estimates of interaction coefficients or trait-based proxies [12]. In species-rich systems, such information is typically unavailable, and incomplete knowledge of niche dimensions can substantially affect the inference of interaction structure [64]. By contrast, the analytical solution of our spatial model integrates dispersal and interaction effects at the community level, requiring only spatial census data for its application. Our vulnerability index therefore provides a flexible, context-dependent measure that accounts for the community composition. That is, a species with low vulnerability in one metacommunity may be at risk in another, depending on both species composition and spatial structure.

We also acknowledge the connection to probabilistic coexistence frameworks:

> Discussion, l. 553
>
> [...] Understanding the factors that drive these variations in transient behavior is an important direction for further investigation [65, 66]. Closely related to this, at very low abundances, demographic fluctuations are expected to become increasingly important. Recent work has highlighted the need to introduce probabilistic frameworks into coexistence theory [10]. In this context, important directions for future work [...]

Together, these additions clarify how our approach complements existing coexistence frameworks by providing

an empirical, spatially explicit measure of proximity to exclusion that can be applied to species-rich systems where detailed parameterization is not feasible.

> ***Reviewer:***
> *5. For my taste, you have not explained your framework well enough to understand what it means biologically. Particularly, the first part where you add in the "biology" it is unclear how you modelled, measured or general "get" the vital rate and interaction coefficients. You might argue that it doesn't matter because the outcome works but models of these vital rates are notoriously basic and reliant on a major assumptions that tend to work in certain types of ecosystems (totally high diversity rainforests) and not others. After reading the methods, I still don't know what biology your model is based on. I came away in the end thinking this is a neutral model and ended with the question, what does this do for us that Hubbell's neutral model does not? Further, the test systems you use are hyper diverse rainforests, one of the few systems that Hubbell's neutral model works in as well. I would be more convinced that this provides useful results for a wide range of systems if you had tested two different types of plant systems - even a temperate forest. What to do about this? Well I want to see the methods explain what biology is in here and how you added species specific biology in. I would love you to use a system other than a rainforest as an empirical test – the rainforests are fine, don't get me wrong, but with an ecological literature that has clearly shown the rainforest diversity behaves differently from the diversity in other systems, this test alone is great but not good enough to convince me that this model will always come to the "right answer." Also, I am unconvinced that many empirical ecologists will use this method unless you can explain how you parameterised the vital rates and competition in your model.*

We thank the reviewer for this important and detailed comment. We realized that the biological interpretation of the model and its empirical applicability were not sufficiently clear in the original manuscript. We have revised the manuscript accordingly and addressed the main points below.

(i) Biological content of the model and its distinction from neutral theory. The model describes species sharing a spatially distributed limiting resource (canopy space), with dynamics driven by local growth, mortality, dispersal, and local interactions. These processes are coupled across species through the constraint $(1 - \sum_\beta p_{\beta i})$, which represents the finite space available within each patch. This interspecific coupling is a key difference from neutral models, where species are assumed to be demographically equivalent and do not interact beyond stochastic replacement.

Species-specific biology enters through the interaction–dispersal kernel $\mathbf{K}_\alpha$, which captures the combined effects of local demographic rates, dispersal, and interactions (in principle, both competitive and facilitative interactions, as the parameters describing the kernel should be thought of as effective parameters). Rather than attempting to measure these components separately, the model provides a coarse-grained description in which their net effect is encoded in a species-specific kernel governing spatial colonization. The statistical properties of $\mathbf{K}_\alpha$ are determined two parameters that vary across species and capture biologically meaningful differences in spatial strategies. In the revised manuscript, we now explicitly emphasize this.

From an empirical perspective, our framework is designed to operate directly on spatial census data. The inference procedure consists of: (i) discretizing the landscape into patches, (ii) computing the vacancy-adjusted abundance (VAA) across patches, (iii) fitting the analytical VAA distribution to infer species parameters, and (iv) determining the shared carrying capacity M self-consistently. This workflow does not require independent measurements of dispersal kernels, interaction coefficients, or vital rates, making the approach applicable to species-rich systems where such data are typically unavailable.

These aspects are now better clarified in multiple parts of the revised manuscript. We now write, when we first introduce the model:

> MAIN, L. 106
>
> We study a general metacommunity model describing the temporal dynamics of species abundances in a habitat composed of N colonizable sites, each providing a finite amount of available space or a limiting resource. These sites may correspond to distinct patches, although our framework does not strictly require explicit physical boundaries. The model incorporates basic processes such as death, reproduction, dispersal, colonization, and competition for space. Their combined effect is captured by an interaction-dispersal kernel matrix, $\boldsymbol{K}$, whose elements represent a coarse-grained description of how species interact and spread across patches. However, they do not explicitly separate local competitive and facilitative interactions, but capture instead their net effect on dispersal efficacy.
> The mathematical structure of the model aligns with the metacommunity framework [13], widely used to study species survival in fragmented landscapes [39, 40, 41]. However, measuring interaction kernels or the corresponding dispersal networks in real ecosystems remains challenging. In light of this experimental inaccessibility, we posit that the intrinsic variability in habitats, species traits, and interspecific interactions in large-scale natural ecosystems will lead to entries in the kernel that are effectively random. We model this randomness by drawing kernel elements from a Gamma distribution (Methods). The parameters of this distribution are species-specific, meaning that each species has its own parameter pair, that quantitatively capture diverse colonization strategies. These parameters are not prescribed a priori, but inferred directly from observed spatial abundance data.

> The link between model and data is provided by a metric that emerges from the analytical solution of the model using generalized dynamical mean field theory (DMFT) [42]: the *vacancy-adjusted abundance* (VAA). The VAA of species α at a given site is defined as the fraction of space it occupies divided by the locally available (unoccupied) space. This normalization accounts for local space saturation, making the VAA dependent on how empty a local site is relative to its capacity. Therefore, a species with the same relative space-use in different subplots contributes differently to the VAA depending on how much each patch is occupied: a subplot with very low free space amplifies the VAA for species occupying this spot, and it reduces it in subplots that have larger free space. Although the VAA arises naturally from the DMFT calculation (Methods), it admits a clear ecological interpretation: by upweighting space-limited patches, it rescales local relative space occupation to reflect the effective colonization effort.
>
> For each species, the parameters of the VAA distribution quantify its dispersal heterogeneity (δ_α^{-1} in table 1) and an effective dispersal strength ($\bar{\beta}_\alpha^{-1}$ in table 1), which depends on the surrounding community. Fitting empirical VAA distributions therefore enables inference of species-specific dispersal strategies without requiring direct measurements of dispersal or interaction parameters. Because the parameters of the theoretical VAAD are linked to those of the interaction-dispersal kernel through an implicit integral equation, fitting the theory to data enables inference of this kernel, which encodes species-specific spatial strategies. In this way, the VAA distribution provides a direct link between observable space-use patterns and the underlying interaction-colonization structure, while remaining a model-independent quantity that can be estimated from spatial abundance data. The overall conceptual pipeline is illustrated in Fig. 1.

This text is accompanied by the fully redesigned fig. 1, which now exclusively focuses on the relationship between the model, the VAA, and the procedure used to parametrize the kernel from the spatial data.

The fully revised Table 1 is also much more explicit in addressing these points. Furthermore, the revised Methods now contain a very detailed explanation addressing these points:

> Methods, l. 825
>
> The kernel matrix $\mathbf{K}_\alpha$ is an *effective* species-specific interaction–colonization kernel. It quantifies both how species interact and how they disperse between patches. The kernel is not measured directly from field data, nor do we attempt to reconstruct each individual kernel entry $K_{\alpha,ij}$ separately. Doing so would require detailed knowledge of local demographic rates, dispersal between all pairs of patches, and interactions in every local environment, which is generally infeasible in species-rich and spatially heterogeneous systems. Instead, we assume that, for each species, the entries of $\mathbf{K}_\alpha$ are drawn from a statistical distribution $\mathcal{Q}_N^{(\alpha)}$ characterized by a small number of species-specific parameters. These parameters capture the variability in interactions and dispersal strategies among species. Although the kernel elements are random, we are able to infer the parameters of $\mathcal{Q}_N^{(\alpha)}$ indirectly from spatial abundance patterns for each species, by exploiting an explicit solution of the model.

For brevity, we do not report here the "Forest data analysis" section of the Methods (at l. 1054), which we have extensively edited to make the explanation of how the model is parametrized more accessible.

Two additional results introduced during revision further support the biological interpretation of the model. First, the new guild analysis discussed in the response to reviewer 1 and presented in the new fig. 2 shows systematic differences in δ_α between ecological guilds at BCI, with gap species exhibiting more aggregated spatial distributions than shade-tolerant species.

> Main, l. 260
>
> Crucially, besides achieving a better fit to the VAA distributions, our model also yields two species-specific parameters that carry clear ecological information: dispersal heterogeneity and effective dispersal strength (table 1). Based on the meaning of the VAA, we hypothesize that VAA heterogeneity also reflects differences in life-history strategies. To test this, we compared parameter values across functional guilds in the BCI dataset (shade-tolerant, gap-exploiting, and intermediate; classification from ref. [49]), focusing on a grouped comparison of gap-exploiting and intermediate species versus shade-tolerant species. We found no statistically significant difference between guilds in the effective dispersal strength (Fig. 2E; see caption for details on the statistical test). In contrast, gap-exploiting and intermediate species exhibit significantly larger dispersal heterogeneity than shade-tolerant species (Fig. 2F). This difference between gap exploiters and shade-tolerant species remains significant when treating all three guilds separately (SI, Sec. 4). These results are consistent with the role of canopy gap dynamics in promoting episodic local dominance of light-demanding species [50, 51], and with recent work highlighting the role of life-history strategies in shaping species responses to environmental variability and enabling coexistence [17, 52].

Second, we introduced a comparison with several ecological spatial null models, among which a neutral null model. Our framework consistently outperformed the neutral model, demonstrating that it can capture and exploit species-specific differences. These results are extensively presented in the supplemental material (sec. 6) and succinctly in the main text:

> Main,l. 243
>
> [...] we compared our framework against ecological null models in which species are independent and overdispersed, leading naturally to a Negative Binomial description of abundances [47]. We considered two variants (Meth-

> ods): a neutral model parameterized at the community level, closely related to neutral theories of biodiversity that reproduce several large-scale ecological patterns in species-rich systems [48], and a species-specific model fitted independently for each species, matching the total number of parameters of our framework. We evaluated model performance by fitting the VAA distributions of all species across the three datasets (see Methods). For each dataset, the absolute goodness-of-fit of our model is consistent with the reference level expected for a correctly specified model (Fig. 2C, dashed line). In contrast, the species-specific model provides an adequate fit only for the Michigan dataset, while the neutral model exhibits substantially larger errors across all datasets, suggesting model misspecification. In a relative goodness-of-fit comparison, our framework consistently outperforms the neutral model across all datasets (positive KS log-ratio in Fig. 2D). Relative to the species-specific model, it yields a marked improvement in Pasoh and Michigan, and a comparable, slightly better performance in BCI. Full details are provided in the SI (Sec. 6), where an intermediate variant with partially shared parameters is also analyzed. Additional analyses of data requirements (SI, Sec. 7) show that reliable parameter estimation is achievable even for smaller datasets and provide lower-bound estimates on data requirements.

(ii) Validation beyond tropical rainforests. To address the concern about ecosystem specificity, we have extended our analysis to include a temperate forest dataset. The Michigan Big Woods dataset, a 23-ha temperate deciduous forest plot in the United States, is now included in all analyses, and these new results are reported throughout the manuscript.

Despite the lower diversity of this ecosystem compared to the two tropical forests, the model provides good fits and consistently outperforms the neutral null model (fig. 2). Parameter estimation remains robust with relatively modest data requirements ($\sim$ 30–46 patches, corresponding to $\sim$ 7–12 ha), as detailed in the new section of the Supplemental Information (Sec. 7). Statistical relationships that depend on the number of species remain significant but have reduced statistical power. Fit quality, out-of-sample predictive accuracy, and improvement over the neutral null remain robust for also the temperate forest, confirming that our framework is not limited to hyper-diverse communities.

Minor comments

> ***Reviewer:***
> *Title: I don't think the title aligns with your paper. Rare species seem a minor part of this study, why is that highlighted in the title?*

We thank the reviewer for this suggestion. We agree that the original title overemphasized rare species relative to the broader scope of the study. We have therefore revised the title to: *"Rare but resilient: dispersal diversity buffers species vulnerability"*. This revised title reflects more accurately that the identified mechanism applies across species.

> ***Reviewer:***
> *Line 26 – I would change "diverse ecosystems" to a "range of different ecosystems" for clarity, the original phrasing can mean two things so might confuse some readers whether you mean any diverse ecosystem or a diverse range of ecosystems.*

We agree. We have replaced "diverse ecosystems" with "a range of ecosystems" in the abstract to avoid ambiguity.

> ***Reviewer:***
> *Lines 36-40 - though you have referenced a lot of papers here, they are all focusing on more traditional paradigms of coexistence. You mention briefly that simplifications are required but you don't cite any of the recent work attempting to increase the complexity of coexistence and diversity model. I see this as problematic, especially as you set up the argument that most models are too simplistic but you have yet to acknowledge those that have attempted to add in complexity (a few suggested references in my major comment section).*

Addressed in our response to Major Comment 1. We have revised the Introduction to incorporate recent work on biologically realistic coexistence and spatial models, including the references suggested by the reviewer, and have replaced several less relevant references accordingly.

> ***Reviewer:***
> *Lines 43-44 – the discussion of the Red List seems odd and out of place here. I guess you are trying to link your paper to conservation but I think it is not necessary and this seems like a clumsy straw man – the Red List is notoriously over simplified (by necessity) but definitely does not represent the cutting edge of species distribution modelling. Maybe you come move this to the discussion as a potential application of this model?*

We agree with the reviewer that the original framing around the IUCN Red List was not well aligned with the main focus of the manuscript. We have therefore removed the explicit reference and revised the paragraph to

provide a more neutral and general overview of existing approaches.

> ***Reviewer:***
> *Lines 46–48 as per your list of papers on the coexistence literature, I feel you have focused on very traditional approaches (see a few suggestions in the major comment section).*

Addressed together with Lines 36–40 (see response to Major Comment 1).

> ***Reviewer:***
> *Line 61 – please define "vulnerability" clearly here – I am sure you are going to get into it, but so much of your paper depends on this term and how you define it, I think it is really important you are clear and upfront about what this means from the very start.*

We agree that this concept should be clearly introduced at first use. We have revised the introductory sentence to define it directly:

> Introduction, l. 84
>
> Here, we introduce an analytical framework to estimate each species' proximity to its competitive-exclusion threshold within spatially structured metacommunities.

The term "vulnerability" is then used consistently with this meaning and defined formally in Table 1 and the Methods.

> ***Reviewer:***
> *Line 63 – why no facilitation? I would think the presence of facilitation, which is a spatially structured process (Bimler et al. 2018) would make a big different to vulnerability.*

We agree and we clarified this in our answer to point 2. Here, we revised the wording to avoid any implication that facilitation is explicitly excluded from the framework, suggesting that only competitive interactions are explicitly modeled:

> Introduction, l. 85
>
> Our approach employs a parsimonious ecological model explicitly incorporating basic processes taking place in a heterogeneous spatial environment. Leveraging analytical methods from the physics of disordered systems, we derive. . . (table 1).

> ***Reviewer:***
> *Table 1 is awesome, I love a glossary and appreciate the inclusion of this table. My only complaint is your Table legend is too brief – it should be clear what is in the table without reading the whole text.*

We have expanded the Table 1 to make it self-contained, including expanded plain-language descriptions and the inference pathway for each quantity.

> ***Reviewer:***
> *Line 73 – watch for terms with double means in the context of ecology. I would not use "viability" on this line as its meaning is ambiguous.*

We have replaced "viability" with "long-term persistence" (and related phrasing) throughout to avoid ambiguity in an ecological context.

> ***Reviewer:***
> *Line 77 – again – do you really mean evolution? Or do you mean the development or assembly? I would not use the word evolution unless you mean it biologically.*

We had used "evolution" in the dynamical-systems sense (temporal change over time). We agree that this is confusing in an ecology context. We have replaced "temporal evolution" with "temporal dynamics" and "evolving" with "progressing to stationarity" (or equivalent phrasing) throughout, to avoid confusion with the biological meaning of evolution.

> ***Reviewer:***
> *Line 90 – how is this species specific without data? I am sure you explain this in the methods (well not really as I have now read the methods) but could you add a sentence explaining the general approach?*

We thank the reviewer for helping us clarify this. From a mathematical perspective, the parameters are species-specific because each species has its own set of parameters, i.e., the parameters are not shared between species but inferred for every single one. We have clarified this point by explicitly stating how species-specific

parameters are obtained from the data. We now write:

> Main, l. 162
>
> The parameters of this distribution are species-specific, meaning that each species has its own parameter pair, that quantitatively capture diverse colonization strategies. These parameters are not prescribed a priori, but inferred directly from observed spatial abundance data.

The new fig. 1 is also showing that the kernel parameters are inferred from data and the revised Methods section has been substantially revised to explain the inference procedure in detail. The passages quoted in our response to the Major Comment 5 also further address this point.

Reviewer:
Figure 1 – your observations seem to get much less accurate at smaller L's. I would like to see if the inclusion of facilitation explains this – it is these smaller scales where you would expect this effect to be strongest.

We agree that the deviations at small spatial scales are important to understand. We have therefore carried out a systematic analysis of scale-dependent model performance (Supplementary Fig. S2), which shows that the fit quality deteriorates at very small scales ($L = 10$–20 m), where patches contain few individuals and the assumptions underlying the analytical solution are less accurate. As discussed in our response to Major Point 2, this deterioration is also consistent with increased finite-data fluctuations, as smaller subplots provide fewer observations per patch. Based on this analysis, we now focus on $L = 50$ m as the optimal spatial scale and present results at this scale in the main text.

Regarding the general role of facilitation in our model, we discussed this in our response to Major Point 2 above. In our explicit test of whether incorporating an effective facilitation term improves the fit, as mentioned there, we found no strong improvement specifically at smaller scales (see also the response to Major Comment 2 and Supplementary Information, Sec. 5).

Reviewer:
OK really hard to understand what the VAA does without reading the methods, I know word limits are tough but you need to explain this a bit more here.

We agree that the VAA concept needed a clearer introduction in the main text. We have therefore expanded its description at first use and revised Figure 1 to include a graphical schematic illustrating its construction. Table 1 now provides a self-contained definition.

Reviewer:
Lines 126-128 – Does your method add up each species competitive balance? Or does it allow for non-linear relationships to emerge? One of the biggest problems with current community models is they assume that communities are the sum of the ecologies of individual species – how does your method move beyond that?

We thank the reviewer for allowing us to clarify this fundamental point. The competitive balance is not additive; our calculations (the DMFT solution of the model) show explicitly that each species' competitive balance is a collective property of the system. Quantitatively, it is defined through a self-consistent relation that couples all species via a shared community-level constraint, which is a highly nonlinear and nontrivial relation.

We have clarified this point in the revised manuscript. We now explicitly state that:

> Main, l. 321
>
> Our calculations provide an explicit integral equation that connects the competitive balance of each species to the VAA distribution parameters of all species within the community (see Tab. 1). The structure of this equation has two important consequences: first, competitive balances are not simply additive, but coupled non-linearly through a shared term that makes community-level coexistence emerge as a collective property rather than a sum of individual contributions; second, it enables the inference of all competitive balances from data. Intuitively, competitive balance reflects how community-wide processes influence each species-specific ratio between factors that increase abundance and those that decrease it. A species can persist if its competitive balance is both greater than one, indicating that it could at least sustain itself in the absence of competition, and not too far below an *equal balance condition*, a hypothetical scenario in which all species share identical balance values.

The corresponding entry in the revised table 1 also makes this point explicit.

Reviewer:
Figure 2 – What is the point of Panel F? How do these plots connect to A-E, not clear from your legend. Is it just the point that you can have different dispersal kernels and the same abundance? I am not convinced this is a great way to make this point, especially since the abundances are not actually that similar. If you

want to keep these, maybe a solution would be to draw an arrow linking the point in E that represents these two species to make the connection clear (or with symbols like in Figure 3 – I like the clear link between panel E and F in Figure 3 more than in Figure 2?). Panel E – the spread seems to shrink as abundances increase – I know this is not your point but why is that?

We thank the reviewer for this suggestion. We agree that panel F was not strictly needed to make our point and was not clearly linked to the other panel, so we decided to remove it.

Regarding panel E, the apparent reduction in parameter spread at higher abundances arises from the coupled constraints imposed by the model structure. The effective dispersal strength depends on both abundance and the inverse rate parameter, which partially compensate at low abundance. At higher values, the inverse rate parameter saturates, causing the increasing trend in $\bar{\beta}_\alpha^{-1}$, the effective dispersal strength. Simultaneously, the local space constraint implies that achieving very high abundance requires increasingly homogeneous spatial distributions, constraining the heterogeneity parameter δ_α^{-1} to lower values. The combination of these effects leads to the observed reduction in spread. As this mechanism is secondary to the main results and requires a relatively detailed explanation, we have chosen not to include it in the main text to preserve clarity, but to move it to the Supplemental Material, sec. 4, where it appears as the new fig. S8.

Reviewer:
Line 209 - I am not completely convinced this is true – at very small spatial scales you see a deviation – it is these small spatial scales at which individuals interact so I think this deviation is really important, not something to be brushed aside. I want to see you spend more time exploring that deviation.

The reviewer is right to point this out. In fact, the additional extensive statistical analyses quoted and discussed in response to previous points above revealed that more care was needed in selecting the primary spatial scale, so we thank the reviewer for pushing us to explore the origin of the deviations. As we explained above, we have dedicated a new Supplementary Section (S2, Supplementary Fig. S2) to a quantitative analysis of scale-dependent fit quality. We removed this sentence because it referred to a panel in the old figure that we do not show anymore in the main text.

Reviewer:
Line 229 – again, I don't think these abundances are that similar. Either explain why these abundances are close enough for your purposes (somewhere, not necessarily here) OR use a different word – high relative abundances for instance?

We have edited the text and the caption of the figure (now Figure 4). We now consider the following two species at BCI, which have a much closer match in terms total abundance: *Prioria copaifera* and *Pterocarpus rohrii*, which have a total stem count counts of 1348 vs. 1380. We state in the manuscript that these species have "comparable total abundance" and quantify the difference (≈ 2% difference in the number of total individuals).

Our point here is mostly to illustrate the results of Figure 4E in terms of spatial patterns: that the partial correlation between species vulnerability and the spatial heterogeneity is negative, meaning that our model predicts that even if two species had similar average abundances but different spatial variability, the one with more heterogeneous dispersal would tend to be a superior competitor. Therefore, in Figure 4F, we chose two species showing this qualitative effect, which is quantified for all species in Figure 4E. Besides using "comparable abundance" in the main text, we have rewritten the relevant part of the main text as follows:

> Main, l. 491
>
> This indicates that even if two species were to have the same average abundances but different spatial variability, the one with more heterogeneous dispersal would tend to be a superior competitor. [...] This effect is visualized for two example species from the BCI dataset that have comparable total abundance (≈ 2% difference) [...]

Reviewer:
Line 234 – Switch to first person: "We present a novel theoretical framework. . . " You are flip flopping from first to third person, which makes it harder to read.

We have rephrased the first two sentences of the Discussion to avoid this flip-flop.

Reviewer:
Line 235 – After this sentence add a sentence that states how this adds to the existing, growing modern literature on biologically realistic coexistence and extinction predictions.

We have edited the Discussion stating how our approach is positioned with respect to the modern literature, including all references suggested in the major points above.

Reviewer:
Lines 253-257 – this is a nice thought, but I suspect the actuals associated with doing this would be quite difficult, so perhaps rephrase here to make it clear that this is a far distant extension, not something you could just pick this framework up and do tomorrow.

We have rephrased this paragraph to clarify that these applications represent conceptual extensions of the framework rather than immediately practical use cases. We now write:

> Discussion, l. 539
>
> More generally, the framework could be extended to investigate hypothetical ecological disturbances such as the selective suppression of a single species, tracking how the vulnerability of the remaining species is altered and whether cascading exclusions analogous to a domino effect would emerge. These applications suggest how the framework may be used to probe the resilience and fragility of ecological networks under perturbations.

Reviewer:
Line 259 – why are you suddenly bringing up evolution? I would think the literature on the spatial dependency of coexistence and (if you want) the evolution of coexistence would be a more relevant point to make here. Your study does not tell us anything about evolutionary processes.

We have replaced "temporal evolution of biodiversity" with "temporal dynamics of biodiversity" throughout to avoid confusion with the biological meaning of evolution.

Reviewer:
Lines 277-278 – again, you have really under referenced this concern...
1. Comins, H. N., & Noble, I. R. (1985). Dispersal, variability, and transient niches: species coexistence in a uniformly variable environment. The American Naturalist, 126(5), 706-723.
2. Lavorel, S., O'Neill, R. V., & Gardner, R. H. (1994). Spatio-temporal dispersal strategies and annual plant species coexistence in a structured landscape. Oikos, 75-88.

We thank the reviewer for these references. Both are relevant and we added them.

Reviewer:
Line 283 – this is not a sufficient mention of facilitation, and though mutualisms may be rare, facilitation is not.

Addressed in detail in our response to Major Comment 2. We have revised the manuscript to clarify the role of facilitation within our framework, including its effective treatment through the coarse-grained interaction–dispersal kernel and the results of additional analyses. We have also added a dedicated paragraph in the Discussion (reported above) to better situate our approach within the facilitation literature and to outline possible extensions in this direction.

Reviewer:
Methods (lines 461-495): I don't feel there is enough detail here for most readers to understand what you are doing... What IS K? you say it is a species-specific kernel, but where does this come from? Is it generated from data? Is it randomly assigned from a distribution?

We thank the reviewer for pointing this out. We agree that the nature of the kernel $\mathbf{K}_\alpha$ was not sufficiently clear in the original manuscript. We have revised the first section of the Methods to introduce the model with more details, and to explicitly clarify that $\mathbf{K}_\alpha$ is an effective interaction–colonization kernel that is not measured directly nor reconstructed entry by entry. Instead, we assume that the kernel for each species is described by a distribution with species-specific parameters (i.e., each species has its own distribution of the kernel). We are able to infer its parameters indirectly from observed spatial abundance patterns using the analytical results of the model. We have added a paragraph in the Methods and further clarified this in the main text to improve accessibility of this technical section to a broader readership. We now write

> Methods, l. 825
>
> The kernel matrix $\mathbf{K}_\alpha$ is an *effective* species-specific interaction–colonization kernel. It quantifies both how species interact and how they disperse between patches. The kernel is not measured directly from field data, nor do we attempt to reconstruct each individual kernel entry $K_{\alpha,ij}$ separately. Doing so would require detailed knowledge of local demographic rates, dispersal between all pairs of patches, and interactions in every local environment, which is generally infeasible in species-rich and spatially heterogeneous systems. Instead, we assume that, for each species, the entries of $\mathbf{K}_\alpha$ are drawn from a statistical distribution $\mathcal{Q}_N^{(\alpha)}$ characterized by a small number of species-specific parameters. These parameters capture the variability in interactions and dispersal strategies among species. Although the kernel elements are random, we are able to infer the parameters of $\mathcal{Q}_N^{(\alpha)}$ indirectly from spatial

> abundance patterns for each species, by exploiting an explicit solution of the model.

Reviewer:
Lines 478-480 – that is all great, but this referencing of other papers is not sufficient to explain your model, especially given that the point of this paper is to present a model that has tangible applications for empirical ecologists.

Addressed together with the Methods revision above. We have expanded the presentation of the model to provide a more self-contained and accessible explanation, rather than relying primarily on references to previous work.

Reviewer:
Line 481-482 – Classical competition models don't do a very good job of explaining real plant communities...

We agree. Indeed our model goes well beyond these approaches. With this sentence, we were only noticing a formal analogy at the level of the mathematical structure of some terms the equations *in a specific limiting case* (a full mean field treatment), not to suggest that classical competition models provide an adequate description of real plant communities, or that our model is equivalent to these classical treatments. We have rephrased the relevant sentence to make this distinction clear and to avoid any implication that our approach relies on a predefined competition hierarchy. We have also stressed – as we did in the main text – that the competition term here is a lumped effect, in the sense that it should be understood as a the net effect between actual competition and reduction of competition through facilitation mechanisms, as our inference procedure does not disentangle these two processes. We write:

> Methods, l. 860
>
> We also note in passing that recasting our model in terms of a spatial mean-field analysis would yield a mathematical structure that has some resemblance to that of classical hierarchical competition models [70]. In our model, however, we do not assume a competition hierarchy *a priori*. Instead, coexistence trade-offs emerge from the interplay between model dynamics and quenched randomness. To uncover such emergent trade-offs, a mean-field analysis is not adequate. Instead, we resort to a dynamical mean-field approach, as explained below.

Reviewer:
Line 495 – just to clarify – these are species-specific but randomly assigned (no biological information), correct?

Not entirely. It is true that the entries of the kernel are treated as random variables, as this allows us to describe spatial heterogeneity statistically and to solve the model using the dynamical mean field theory. However, the parameters of this distribution are species-specific and are not random nor assigned arbitrarily: they are inferred (indirectly through the VAA) from observed spatial abundance patterns using the analytical results of the model. In this sense, the randomness reflects unresolved local variability, while the inferred parameters capture species-specific biological differences in an effective manner. We have clarified this point in the Methods through multiple targeted edits to avoid possible confusion. One specific sentence relating to this point is:

> Methods, l. 830
>
> [...] we assume that, for each species, the entries of $\mathbf{K}_\alpha$ are drawn from a statistical distribution $\mathcal{Q}_N^{(\alpha)}$ characterized by a small number of species-specific parameters. These parameters capture variability in interactions and dispersal strategies among species. Although the kernel elements are random, we are able to infer the parameters of $\mathcal{Q}_N^{(\alpha)}$ indirectly from spatial abundance patterns for each species, by exploiting an explicit solution of the model.

Reviewer:
Line 512 – OK, great but how? Can you do a train and test sort of analysis here?

We thank the reviewer for this suggestion. We now include in the main text an explicit out-of-sample spatial cross-validation (Figure 2B). For each dataset, the forest is randomly split into two equally sized halves; model parameters are fitted on one half, and the predicted mean patch occupancy $\langle p_\alpha \rangle$ is evaluated on the held-out half. The predictions closely match the observations. We also perform parameter cross-validation. Details are provided in the revised Methods and in the Supplementary Information.

Reviewer:
Lines 542-546 – Am I correct that this basically explains how you would go about the suggested next step on lines 253-257? If so, I would link this in the text.

We thank the reviewer for this insightful suggestion. The referenced Methods subsection indeed characterizes the transition boundaries in parameter space that determine species exclusion, and thus provides, in principle, a route to assess the effect of removing species from the community. However, this approach becomes computationally intractable in species-rich systems, as the number of possible configurations grows factorially with the number of species. This limitation motivates the introduction of the vulnerability index in the subsequent subsection, which provides a scalable alternative by quantifying proximity to exclusion without requiring enumeration of all configurations. We have added a brief remark in the manuscript to clarify this connection:

> Methods, l. 984
>
> We note that, in principle, recomputing these transition manifolds iteratively could be used to predict how the removal or introduction of species affects community stability, as discussed in the outlook (Discussion). However, the number of such boundaries grows combinatorially with the number of species S, rendering this approach impractical in species-rich ecosystems and motivating the use of the vulnerability index as a scalable alternative.

Reviewer:
Line 580 - I still don't like the use of the word "evolving" to explain your model progression – please change throughout.

We have replaced the term "evolving" (used in the numerical-simulation context to describe a dynamical system progressing through time) with "progressing to stationarity" (or equivalent phrasing) throughout, to avoid confusion with its biological meaning.

References

[1] Barnosky, A. D., Matzke, N., Tomiya, S., Wogan, G. O. U., Swartz, B., Quental, T. B., Marshall, C., McGuire, J. L., Lindsey, E. L., Maguire, K. C., Mersey, B. & Ferrer, E. A. Has the earth's sixth mass extinction already arrived? *Nature* **471**, 51–57 (2011).

[2] Cowie, R. H., Bouchet, P. & Fontaine, B. The sixth mass extinction: fact, fiction or speculation? *Biological Reviews* **97**, 640–663 (2022). URL `https://onlinelibrary.wiley.com/doi/abs/10.1111/brv.12816`.

[3] Levin, S. A. Multiple scales and the maintenance of biodiversity. *Ecosystems* **3**, 498–506 (2000).

[4] Tilman, D., Clark, M., Williams, D. R., Kimmel, K., Polasky, S. & Packer, C. Future threats to biodiversity and pathways to their prevention. *Nature* **546**, 73–81 (2017).

[5] Chesson, P. Mechanisms of maintenance of species diversity. *Annual review of Ecology and Systematics* **31**, 343–366 (2000).

[6] Saavedra, S., Stouffer, D. B., Uzzi, B. & Bascompte, J. Strong contributors to network persistence are the most vulnerable to extinction. *Nature* **478**, 233–235 (2011).

[7] Levine, J. M., Bascompte, J., Adler, P. B. & Allesina, S. Beyond pairwise mechanisms of species coexistence in complex communities. *Nature* **546**, 56–64 (2017).

[8] James, A. R. M., Mayfield, M. M. & Bimler, M. D. Facilitation thinking for coexistence theory. *Ecology Letters* **28**, e70150 (2025).

[9] Ellner, S. P., Snyder, R. E., Adler, P. B. & Hooker, G. An expanded modern coexistence theory for empirical applications. *Ecology Letters* **22**, 3–18 (2019).

[10] Bowler, C. H., Weiss-Lehman, C., Towers, I. R., Mayfield, M. M. & Shoemaker, L. G. Accounting for demographic uncertainty increases predictions for species coexistence: A case study with annual plants. *Ecology Letters* **25**, 1618–1628 (2022).

[11] Grilli, J., Adorisio, M., Suweis, S., Barabás, G., Banavar, J. R., Allesina, S. & Maritan, A. Feasibility and coexistence of large ecological communities. *Nature Communications* **8**, 14389 (2017). URL `https://doi.org/10.1038/ncomms14389`.

[12] Spaak, J. W. & De Laender, F. Intuitive and broadly applicable definitions of niche and fitness differences. *Ecology Letters* **23**, 1117–1128 (2020).

[13] Hanski, I. Metapopulation dynamics. *Nature* **396**, 41–49 (1998).

[14] Leibold, M. A., Holyoak, M., Mouquet, N., Amarasekare, P., Chase, J. M., Hoopes, M. F., Holt, R. D., Shurin, J. B., Law, R., Tilman, D. *et al.* The metacommunity concept: a framework for multi-scale community ecology. *Ecology letters* **7**, 601–613 (2004).

[15] Grainger, T. N. & Gilbert, B. Dispersal and diversity in experimental metacommunities: linking theory and practice. *Oikos* **125**, 1213–1223 (2016).

[16] Loreau, M. & De Mazancourt, C. Biodiversity and ecosystem stability: a synthesis of underlying mechanisms. *Ecology letters* **16**, 106–115 (2013).

[17] Jops, K. & O'Dwyer, J. P. Life history complementarity and the maintenance of biodiversity. *Nature* **618**, 986–991 (2023).

[18] Harte, J. & Newman, E. A. Maximum information entropy: a foundation for ecological theory. *Trends in Ecology & Evolution* **29**, 384–389 (2014).

[19] Allesina, S. & Tang, S. Stability criteria for complex ecosystems. *Nature* **483**, 205–208 (2012).

[20] Levin, S. A. The problem of pattern and scale in ecology. *Ecology* **73**, 1943–1967 (1992). URL https://esajournals.onlinelibrary.wiley.com/doi/abs/10.2307/1941447.

[21] Hart, S. P., Usinowicz, J. & Levine, J. M. The spatial scales of species coexistence. *Nature Ecology & Evolution* **1**, 1066–1073 (2017).

[22] Keil, P., Wiegand, T., Tóth, A. B., McGlinn, D. J. & Chase, J. M. Measurement and analysis of interspecific spatial associations as a facet of biodiversity. *Ecological Monographs* **91**, e01452 (2021).

[23] García-Callejas, D., Bartomeus, I. & Godoy, O. The spatial configuration of biotic interactions shapes coexistence-area relationships in an annual plant community. *Nature Communications* **12**, 6192 (2021).

[24] Wiegand, T., Grabarnik, P. & Stoyan, D. Envelope tests for spatial point patterns with and without simulation. *Ecosphere* **7**, e01365 (2016).

[25] Harris, D. J. Generating realistic assemblages with a joint species distribution model. *Methods in Ecology and Evolution* **6**, 465–473 (2015).

[26] Wiegand, T., Wang, X., Anderson-Teixeira, K. J., Bourg, N. A., Cao, M., Ci, X., Davies, S. J., Hao, Z., Howe, R. W., Kress, W. J., Lian, J., Li, J., Lin, L., Lin, Y., Ma, K., McShea, W., Mi, X., Su, S.-H., Sun, I.-F., Wolf, A., Ye, W. & Huth, A. Consequences of spatial patterns for coexistence in species-rich plant communities. *Nature Ecology & Evolution* **5**, 965–973 (2021).

[27] Kalyuzhny, M., Lake, J. K., Wright, S. J. & Ostling, A. M. Pervasive within-species spatial repulsion among adult tropical trees. *Science* **381**, 563–568 (2023).

[28] Wiegand, T. *et al.* Latitudinal scaling of aggregation with abundance and coexistence in forests. *Nature* **640**, 967–973 (2025).

[29] Pilowsky, J. A., Colwell, R. K., Rahbek, C. & Fordham, D. A. Process-explicit models reveal the structure and dynamics of biodiversity patterns. *Science Advances* **8**, eabj2271 (2022). URL https://www.science.org/doi/abs/10.1126/sciadv.abj2271.

[30] Elith, J. & Leathwick, J. R. Species distribution models: Ecological explanation and prediction across space and time. *Annual Review of Ecology, Evolution, and Systematics* **40**, 677–697 (2009). URL https://www.annualreviews.org/content/journals/10.1146/annurev.ecolsys.110308.120159.

[31] Kunin, W. E. Extrapolating species abundance across spatial scales. *Science* **281**, 1513–1515 (1998). URL https://www.science.org/doi/abs/10.1126/science.281.5382.1513.

[32] Wilson, R. J., Thomas, C. D., Fox, R., Roy, D. B. & Kunin, W. E. Spatial patterns in species distributions reveal biodiversity change. *Nature* **432**, 393–396 (2004).

[33] Shaffer, M. L. Minimum population sizes for species conservation. *BioScience* **31**, 131–134 (1981). URL https://doi.org/10.2307/1308256.

[34] Vindenes, Y., Le Coeur, C., Caswell, H. *et al.* Introduction to matrix population models. *Demographic methods across the tree of life* 163–179 (2021).

[35] Chaudhary, V. & Oli, M. K. A critical appraisal of population viability analysis. *Conservation Biology* **34**, 26–40 (2020). URL https://conbio.onlinelibrary.wiley.com/doi/abs/10.1111/cobi.13414.

[36] Simmonds, E. G. & Jones, O. R. Uncertainty propagation in matrix population models: Gaps, importance and guidelines. *Methods in Ecology and Evolution* **15**, 427–438 (2024). URL https://besjournals.onlinelibrary.wiley.com/doi/abs/10.1111/2041-210X.14276.

[37] Simini, F., Anfodillo, T., Carrer, M., Banavar, J. R. & Maritan, A. Self-similarity and scaling in forest communities. *Proceedings of the National Academy of Sciences* **107**, 7658–7662 (2010).

[38] Martínez Cano, I., Muller-Landau, H. C., Wright, S. J., Bohlman, S. A. & Pacala, S. W. Tropical tree height and crown allometries for the barro colorado nature monument, panama: a comparison of alternative hierarchical models incorporating interspecific variation in relation to life history traits. *Biogeosciences* **16**, 847–862 (2019).

[39] Hanski, I. & Ovaskainen, O. The metapopulation capacity of a fragmented landscape. *Nature* **404**, 755–758 (2000).

[40] Mouquet, N. & Loreau, M. Coexistence in metacommunities: the regional similarity hypothesis. *The American Naturalist* **159**, 420–426 (2002).

[41] Padmanabha, P., Nicoletti, G., Bernardi, D., Suweis, S., Azaele, S., Rinaldo, A. & Maritan, A. Landscape and environmental heterogeneity support coexistence in competitive metacommunities. *Proceedings of the National Academy of Sciences* **121** (2024).

[42] Azaele, S. & Maritan, A. Generalized dynamical mean field theory for non-gaussian interactions. *Physical Review Letters* **133**, 127401 (2024).

[43] Condit, R., Pérez, R., Aguilar, S., Lao, S., Foster, R. & Hubbell, S. Complete data from the barro colorado 50-ha plot: 423617 trees, 35 years (2019).

[44] Manokaran, N., Seng, Q., Ashton, P., LaFrankie, J., Nur Supardi, N., Ahmad, W. & Okuda, T. Pasoh forest dynamics plot, peninsular malaysia. tropical forest diversity and dynamism: findings from a large-scale plot network (2004).

[45] Allen, D., Vandermeer, J., Dick, C. W., Perfecto, I. & Burnham, R. J. Michigan big woods research plot data (2019).

[46] McIntire, E. J. B. & Fajardo, A. Facilitation as a ubiquitous driver of biodiversity. *New Phytologist* **201**, 403–416 (2014).

[47] Lindén, A. & Mäntyniemi, S. Using the negative binomial distribution to model overdispersion in ecological count data. *Ecology* **92**, 1414–1421 (2011).

[48] Volkov, I., Banavar, J. R., Hubbell, S. P. & Maritan, A. Neutral theory and relative species abundance in ecology. *Nature* **424**, 1035–1037 (2003).

[49] Comita, L. S., Aguilar, S., Pérez, R., Lao, S. & Hubbell, S. P. Patterns of woody plant species abundance and diversity in the seedling layer of a tropical forest. *Journal of Vegetation Science* **18**, 163–174 (2007).

[50] Brokaw, N. & Busing, R. T. Niche versus chance and tree diversity in forest gaps. *Trends in Ecology & Evolution* **15**, 183–188 (2000).

[51] Kupers, S. J., Wirth, C., Engelbrecht, B. M. J., Hernández, A., Condit, R., Wright, S. J. & Rüger, N. Performance of tropical forest seedlings under shade and drought: an interspecific trade-off in demographic responses. *Scientific Reports* **9** (2019).

[52] Jops, K., Dalling, J. W. & O'Dwyer, J. P. Life history is a key driver of temporal fluctuations in tropical tree abundances. *Proceedings of the National Academy of Sciences* **122**, e2422348122 (2025). URL https://www.pnas.org/doi/abs/10.1073/pnas.2422348122.

[53] Nicoletti, G., Padmanabha, P., Azaele, S., Suweis, S., Rinaldo, A. & Maritan, A. Emergent encoding of dispersal network topologies in spatial metapopulation models. *Proceedings of the National Academy of Sciences* **120**, e2311548120 (2023). URL https://www.pnas.org/doi/abs/10.1073/pnas.2311548120.

[54] Tilman, D. The resource-ratio hypothesis of plant succession. *The American Naturalist* **125**, 827–852 (1985). URL https://doi.org/10.1086/284382.

[55] Mayfield, M. M. & Levine, J. M. Opposing effects of competitive exclusion on the phylogenetic structure of communities. *Ecology Letters* **13**, 1085–1093 (2010). URL `https://doi.org/10.1111/j.1461-0248.2010.01509.x`.

[56] Bimler, M. D., Stouffer, D. B., Lai, H. R. & Mayfield, M. M. Accurate predictions of coexistence in natural systems require the inclusion of facilitative interactions and environmental dependency. *Journal of Ecology* **106**, 1839–1852 (2018).

[57] Levins, R. & Culver, D. Regional coexistence of species and competition between rare species. *Proceedings of the National Academy of Sciences* **68**, 1246–1248 (1971).

[58] Levin, S. A. Dispersion and population interactions. *The American Naturalist* **108**, 207–228 (1974). URL `https://doi.org/10.1086/282900`.

[59] Durrett, R. & Levin, S. Spatial aspects of interspecific competition. *Theoretical Population Biology* **53**, 30–43 (1998). URL `https://www.sciencedirect.com/science/article/pii/S0040580997913381`.

[60] Miller, Z. R., Clenet, M., Libera, K. D., Massol, F. & Allesina, S. Coexistence of many species under a random competition–colonization trade-off. *Proceedings of the National Academy of Sciences* **121**, e2314215121 (2024). URL `https://www.pnas.org/doi/abs/10.1073/pnas.2314215121`.

[61] Levine, J. M. & Rees, M. Coexistence and relative abundance in annual plant assemblages: the roles of competition and colonization. *The American Naturalist* **160**, 452–467 (2002).

[62] Clark, J. S., LaDeau, S. & Ibanez, I. Fecundity of trees and the colonization–competition hypothesis. *Ecological monographs* **74**, 415–442 (2004).

[63] Vellend, M. *9. Empirical Evidence: Ecological Drift and Dispersal*, 138–157 (Princeton University Press, Princeton, 2017). URL `https://doi.org/10.1515/9781400883790-010`.

[64] D'Andrea, R., Ostling, A. & O'Dwyer, J. P. Translucent windows: how uncertainty in competitive interactions impacts detection of community pattern. *Ecology Letters* **21**, 826–835 (2018).

[65] Comins, H. & Noble, I. Dispersal, variability, and transient niches: species coexistence in a uniformly variable environment. *The American Naturalist* **126**, 706–723 (1985).

[66] Lavorel, S., O'Neill, R. V. & Gardner, R. H. Spatio-temporal dispersal strategies and annual plant species coexistence in a structured landscape. *Oikos* 75–88 (1994).

[67] Losapio, G., Schöb, C., Staniczenko, P. P. A., Carrara, F., Palamara, G. M., De Moraes, C. M., Mescher, M. C., Brooker, R. W., Butterfield, B. J., Callaway, R. M., Cavieres, L. A., Kikvidze, Z., Lortie, C. J., Michalet, R., Pugnaire, F. I. & Bascompte, J. Network motifs involving both competition and facilitation predict biodiversity in alpine plant communities. *Proceedings of the National Academy of Sciences* **118**, e2005759118 (2021).

[68] Pichon, B., Gounand, I., Donnet, S. & Kéfi, S. The interplay of facilitation and competition drives the emergence of multistability in dryland plant communities. *Ecology* **105**, e4369 (2024).

[69] Buche, L., Shoemaker, L. G., Hallett, L. M., Bartomeus, I., Vesk, P., Weiss-Lehman, C., Mayfield, M. & Godoy, O. A continuum from positive to negative interactions drives plant species' performance in a diverse community. *Ecology Letters* **28**, e70059 (2025).

[70] Tilman, D. Competition and biodiversity in spatially structured habitats. *Ecology* **75**, 2–16 (1994). URL `https://esajournals.onlinelibrary.wiley.com/doi/abs/10.2307/1939377`.

[71] Tovo, A., Suweis, S., Formentin, M., Favretti, M., Volkov, I., Banavar, J. R., Azaele, S. & Maritan, A. Upscaling species richness and abundances in tropical forests. *Science Advances* **3**, e1701438 (2017).

Response to Reviewers (minor revision)

Dispersal diversity buffers species vulnerability to local extinction

Manuscript NATECOLEVOL-25082966A

D. Bernardi, G. Nicoletti, P. Padmanabha, S. Suweis, S. Azaele, S. Levin, A. Rinaldo, A. Maritan

We thank both Reviewers for their positive assessment of the revised manuscript and for the additional comments. Here, we address Reviewer 1's remaining comment on the reporting of the fitted carrying capacities, as well as Reviewer 2's suggestions concerning the title and other textual improvements.

Response to Reviewer 1

> ***Reviewer:***
> *I thank the authors for a comprehensive response to my comments and questions from the earlier draft. I think that the model and new way of computing diversity via the VAA could be highly impactful. The data overhead required to parametrize the model could be prohibitive in some sites, but that doesn't mean that where we have those data, the model isn't effective and impressive.*
> *I have one additional comment. The authors clarified their approach to computing carrying capacity, M, nicely. And it is reassuring that M scales linearly with the choice of patch area across each of the three sites. However, I did not see this scaling with L reported in Section 4 of the Supplementary information, as stated in the response letter (this section deals with the question of how heterogeneous might be the carrying capacity across each of the sites).*
> *Apologies if I did miss it somewhere else, but I think it would be useful to report the actual carrying capacities M across each location. When the currency of stem abundance is used, a nice check would be that M exceeds the most dense of the patches. When the currency of crown area is used, it should be that M scales roughly as n_layer * L^2, where n_layer is the expected number of vertical layers in each site. If this number is unreasonably large then that would be worth noting, and there is a growing literature on quantifying vertical layers that this coefficient could make contact with (e.g. Terborgh 1985 Am Nat, or more recently D'Andrea et al (2020) Ecology).*

We thank the reviewer for this comment, and we apologize for the incorrect reference in our previous response letter: the $M \propto L^2$ scaling result (the M/L^2 density shown in the inset of fig. S1, referenced from the main text) is presented in Supplementary Information Sec. 1 ("Vacancy-adjusted abundance fitting and metric selection"), not Sec. 4.

Following the reviewer's suggestion, we performed additional consistency checks on the carrying capacity estimated from both metrics. Consequently, we added a new table to SI Sec. 1 (Table S1) reporting, for each dataset at $L = 50$ m, the fitted carrying capacities M_c (DBH-weighted/crown metric) and M_s (stem-count metric), together with the two physical consistency checks suggested by the reviewer.

- For the stem-count metric, M_s exceeds the stem count of the most densely occupied patch in all three datasets, as required for a physically valid VAA denominator.
- For the crown metric, we converted the fitted capacity density M_c/L^2 into an absolute canopy-area density using the community-level crown-area allometry $C \approx 0.66\, D^{1.34}$ taken from Martínez-Cano et al. 2019, *Biogeosciences*. From this, we obtained an estimated number of stacked canopy layers, n_{layer}. The result comes out in good agreement with the classical division of humid forests into about four vertical strata (shrub, understory, midstory, canopy).

These additional checks are presented in detail in the revised SI text (Sec. 1), which now reads:

> SI, Sec. 1
> For completeness, we also report the fitted carrying capacities themselves and an absolute physical consistency check. Table S1 lists M_c and M_s at $L = 50$ m for all three datasets, together with the ratio M_s to the stem count of the most densely occupied patch: in all three datasets M_s exceeds this maximum, as required for a physically

> valid denominator in the VAA. To convert the DBH-weighted capacity density M_c/L^2 into an absolute canopy-area density, we use the community-level crown-area allometry $C \approx 0.66\, D^{1.34}$ (C in m^2, D in cm) reported for BCI by [1]. Dividing the resulting crown-area density by the patch ground area L^2 gives an estimated number of stacked canopy layers n_{layer} (Table S1), which comes out to 3-5 across all three datasets. This is in good agreement with the classical division of humid forests into about four vertical strata (shrub, understory, midstory, canopy), reported by [2] and confirmed quantitatively from height-based clustering of BCI tree species by [3]. Note that the n_{layer} estimates for Pasoh and Michigan (marked † in Table S1) are obtained by reusing the BCI-specific a_0. These two estimates should therefore be read as more qualitative than the BCI one, though the fact that they still come out physically reasonable (matching the same four-layer stratification) is reassuring.

A compact reference to this new finding was also added to the main text:

> METHODS L. 665
>
> [...] We verified that both metrics yield consistent estimates of the effective per-patch carrying capacity, and that converting the DBH-weighted carrying capacity to actual canopy area using allometric scaling coefficients for BCI [1] yields an estimated number of canopy layers consistent with those reported for humid forests [2, 3] (see SI, sec. 1). [...]

Response to Reviewer 2

> ***Reviewer:***
> *Thank you for the thorough revision of your manuscript. I think the changes you have made make this a much stronger contribution, with clarifying text and analyses making your study more accessible to a wider audience. I am particularly pleased to see the addition of the temperate forest dataset and the train and test analyses, I think these strengthen your case for the generalisable value of this model a lot. Overall, I am very happy with this revised manuscript. I have made a few tiny suggestions on how to clarify the text in a couple of places - particularly in the abstract and introduction. The only thing I am still not satisfied with is your title. I still don't think it reflects the main message of your model and study. I have suggested a couple of alternative options. I don't expect you to use my suggestions but I do ask that you think about what title will attract the audience you are after. The focus on rarity still seems misplaced. My concern here is that you won't get as many people reading your paper with this title as if you used a title that highlighted the novelty of your model and the utility of it for capturing ecological processes effectively with regular and commonly available tree census data.*

We thank the reviewer for their positive assessment of the revised manuscript, and for these final, targeted suggestions.

Title We thank the reviewer for pressing us on this point, and we agree. We have adopted the reviewer's first suggested alternative, changing the title from "Rare but Resilient: Dispersal diversity buffers species vulnerability" to

> TITLE
>
> Dispersal diversity buffers species vulnerability to local extinction

Abstract and introduction wording

> ***Reviewer:***
> *[Abstract] "...this metric quantifies a focal species' vulnerability beyond its abundance by incorporating the diversity of dispersal strategies..." — this doesn't make sense.*

We have reworded this sentence to improve its clarity, and such that "vulnerability" is now explicitly tied to competitive exclusion at first use (see also points below):

> ABSTRACT
>
> This metric quantifies a focal species' vulnerability to competitive exclusion beyond what is captured by its abundance alone by incorporating the diversity of dispersal strategies and the structure of interspecific interactions within the community.

> ***Reviewer:***
> *[Abstract] "Our results reveal that greater heterogeneity in dispersal strategies reduces vulnerability for a given abundance." — vulnerability to what?*

In the revised text, we have specified that the vulnerability is to competitive exclusion. The sentence now reads:

> ABSTRACT
>
> Our results reveal that greater heterogeneity in dispersal strategies reduces vulnerability to competitive exclusion for a given abundance.

Reviewer:
[Introduction] "... These metrics define species vulnerability as their proximity to the competitive-exclusion threshold..." — in the abstract, consider using "vulnerability to competitive exclusion".

We accepted this suggestion and changed the abstract accordingly (see point above).

Reviewer:
[Introduction] "... and that can be ultimately confronted with empirical data." — I don't think that is the right word, do you mean 'validated'?

We agree, and we have replaced "confronted" with "validated", as the reviewer suggested. The sentence now reads:

> INTRODUCTION
>
> ...theoretical frameworks that link local population dynamics to spatial community structure across scales, and that can be ultimately validated with empirical data.

Reviewer:
[Main text, VAA definition] "... it admits a clear ecological interpretation..." — admits is the wrong word, maybe 'represents'?

We agree, and we have replaced "admits" with the simpler "has". The sentence now reads:

> MAIN TEXT, VAA DEFINITION
>
> Although the VAA arises naturally from the DMFT calculation (Methods), it has a clear ecological interpretation: by upweighting space-limited patches, it rescales local relative space occupation to reflect the effective colonization effort.

Reviewer:
*[Results, guild comparison] "... tolerant species (p = 0.004; Mann-Whitney U see SI for complete statistical report), indicating that shade-tolerant species display more spatially homogeneous abundance distributions." — can you just explicitly state the ** shows a significant difference for clarity?*

We have added an explicit statement of the significance markers to the Fig. 2 caption:

> FIG. 2 CAPTION
>
> Gap and intermediate species have significantly higher dispersal heterogeneity δ_α^{-1} than shade-tolerant species (F: $U = 969$, $p = 0.004$, ******), indicating that shade-tolerant species display more spatially homogeneous abundance distributions... Significance markers: ns $p > 0.05$, ***** $p \leq 0.05$, ****** $p \leq 0.01$.

References

[1] I. Martínez Cano, H. C. Muller-Landau, S. J. Wright, S. A. Bohlman, S. W. Pacala, Tropical tree height and crown allometries for the Barro Colorado Nature Monument, Panama: a comparison of alternative hierarchical models incorporating interspecific variation in relation to life history traits. *Biogeosciences* **16** (4), 847–862 (2019), doi:https://doi.org/10.5194/bg-16-847-2019.

[2] J. Terborgh, The Vertical Component of Plant Species Diversity in Temperate and Tropical Forests. *The American Naturalist* **126** (6), 760–776 (1985), doi:https://doi.org/10.1086/284452.

[3] R. D'Andrea, *et al.*, Counting niches: Abundance-by-trait patterns reveal niche partitioning in a Neotropical forest. *Ecology* **101** (6), e03019 (2020), doi:https://doi.org/10.1002/ecy.3019.
